# Discovery of Prognostic Markers for Early-Stage High-Grade Serous Ovarian Cancer by Maldi-Imaging

**DOI:** 10.3390/cancers12082000

**Published:** 2020-07-22

**Authors:** Hagen Kulbe, Oliver Klein, Zhiyang Wu, Eliane T. Taube, Wanja Kassuhn, David Horst, Silvia Darb-Esfahani, Paul Jank, Salem Abobaker, Frauke Ringel, Andreas du Bois, Florian Heitz, Jalid Sehouli, Elena I. Braicu

**Affiliations:** 1 Tumorbank Ovarian Cancer Network, Charité – Universitätsmedizin Berlin, corporate member of Freie Universität Berlin, Humboldt-Universität zu Berlin, and Berlin Institute of Health, 10117 Berlin, Germany; hagen.kulbe@charite.de (H.K.); wanja.kassuhn@charite.de (W.K.); salem-nuri.abobaker@charite.de (S.A.); frauke.ringel@charite.de (F.R.); Jalid.Sehouli@charite.de (J.S.); 2 Department of Gynecology, European Competence Center for Ovarian Cancer, Charité Universitätsmedizin Berlin, corporate member of Freie Universität Berlin, Humboldt-Universität zu Berlin, and Berlin Institute of Health, 10117 Berlin, Germany; 3 BIH Center for Regenerative Therapies BCRT, Charité – Universitätsmedizin Berlin, 10117 Berlin, Germany; oliver.klein@charite.de (O.K.); zhiyang.wu@charite.de (Z.W.); 4 Institute of Pathology, Charité – Universitätsmedizin Berlin, corporate member of Freie Universität Berlin, Humboldt-Universität zu Berlin, and Berlin Institute of Health, 10117 Berlin, Germany; eliane.taube@charite.de (E.T.T.); david.horst@charite.de (D.H.); paul.jank@uni-marburg.de (P.J.); 5 Institute of Pathology Berlin-Spandau and Berlin-Buch, 10117 Berlin, Germany; s.darb-esfahani@ifp-spandau.de; 6 Institute of Pathology, Philipps-University Marburg, 35032 Marburg, Germany; 7 Evangelische Kliniken Essen-Mitte Klinik für Gynäkologie und gynäkologische Onkologie, 45136 Essen, GermanyF.Heitz@kem-med.com (F.H.)

**Keywords:** ovarian cancer, early-stage HGSOC, prognostic markers, MALDI-IMS

## Abstract

With regard to relapse and survival, early-stage high-grade serous ovarian (HGSOC) patients comprise a heterogeneous group and there is no clear consensus on first-line treatment. Currently, no prognostic markers are available for risk assessment by standard targeted immunohistochemistry and novel approaches are urgently required. Here, we applied MALDI-imaging mass spectrometry (MALDI-IMS), a new method to identify distinct mass profiles including protein signatures on paraffin-embedded tissue sections. In search of prognostic biomarker candidates, we compared proteomic profiles of primary tumor sections from early-stage HGSOC patients with either recurrent (RD) or non-recurrent disease (N = 4; each group) as a proof of concept study. In total, MALDI-IMS analysis resulted in 7537 spectra from the malignant tumor areas. Using receiver operating characteristic (ROC) analysis, 151 peptides were able to discriminate between patients with RD and non-RD (AUC > 0.6 or < 0.4; *p* < 0.01), and 13 of them could be annotated to proteins. Strongest expression levels of specific peptides linked to Keratin type1 and Collagen alpha-2(I) were observed and associated with poor prognosis (AUC > 0.7). These results confirm that in using IMS, we could identify new candidates to predict clinical outcome and treatment extent for patients with early-stage HGSOC.

## 1. Introduction

Epithelial ovarian cancer (EOC) is the leading cause of death within gynecological cancers in the developed countries (http://seer.cancer.gov). Due to the lack of specific symptoms, EOC is often detected at an advanced stage with a five-year survival rate less than 40% [1]. However, 25% of EOC patients are diagnosed in early stage (I-II) as defined by Fédération Internationale de Gynécologie et d’Obstétrique (FIGO), where the disease is often cured by surgery alone, or in combination with platinum-based chemotherapy [2,3]. Even though the prognosis of patients with FIGO stage I-II increases dramatically with treatment, with five-year survival rates between 80–90%, some subgroups of early-stage EOC will relapse and 20–30% of these patients will finally succumb to the disease [4,5,6]. Older age, greater stage, higher grade and malignant cytology are independent prognostic factors for recurrence [7]. Moreover, the prognosis differs between the histological subtypes with high-grade serous ovarian cancer (HGSOC) being the most common one, accounting for 70–80% of ovarian cancer-related deaths.

According to guidelines of the European Society for Medical Oncology (ESMO), bilateral salpingo-oophorectomy, hysterectomy, omentectomy, peritoneal stripping and lymph node sampling are recommended procedures for stage I and II HGSOC patients (https://www.esmo.org/guidelines/gynaecological-cancers/newly-diagnosed-and-relapsed-epithelial-ovarian-carcinoma/esmo-esgo-consensus-conference-recommendations-on-ovarian-cancer) [8,9]. However, fertilization-sparing surgery (FSS) for women of childbearing age could be considered, and be discussed individually [10]. Different criteria for selecting patients have been applied and the debate over FSS in HGSOC is more than controversial as there are limited data on that issue. Preoperative screening methods and comprehensive surgical staging for accurate disease classification are mandatory [11,12]. In this context, one third of presumed stage I ovarian cancers were found to be upstaged by the findings of dissemination in the peritoneal cavity [13]. Patients with high-risk early-stage EOC, defined as stage I, grade 3, stage IC and II, as well as clear cell cancers, will require adjuvant chemotherapy which has been shown to reduce the relapse rate by >60% in stage IC EOC patients [14]. Hence, platinum-based chemotherapy is an important factor in treating these patients with high-risk early-stage EOC with impact on both recurrence-free (RFS) and overall survival (OS). Prognostic markers are needed to stratify patients into low- and high-risk groups in order to select patients who will benefit from chemotherapy. The term EOC refers to at least four different histological subtypes which is an important issue to take into account in the risk assessment of clinical progression. The most aggressive histotype is HGSOC. Nevertheless, the optimal clinical management is still a controversial debate and patients with early-stage high-grade serous EOC might be over-treated which could potentially result in complications after radical surgical management and an increase in toxicity of chemotherapy [15,16]. Hence, it is of utmost importance to identify novel diagnostic markers for this patient cohort in order to improve the risk assessment of tumor recurrence. An optimal evaluation of risk for progression would have the benefit of personalized chemotherapy, and reduced costs and treatment side effects in patients with little risk for progression. Commonly used tissue-based techniques, such as liquid chromatography-based mass spectrometry or gene expression profiling, require large amounts of tissue material. Moreover, these methods do not enable a direct correlation between differentially expressed molecular profiles and the tissue histology [17]. Matrix-assisted laser desorption/ionization (MALDI) imaging mass spectrometry (IMS) has the advantage of combining morphological features with protein expression in tissue. This technique enables spatially resolved tissue assessment via specific molecular signatures (e.g., proteins, peptides, lipids and molecules of cell metabolites) and allows their correlation with alterations in tissue histology [18,19,20] as well as stages of ovarian cancer [21].

This recently developed diagnostic method of imaging mass spectrometry (MALDI-imaging MS) has also been used for the rapid diagnosis and prognosis of patients [22,23,24], and to identify peptide profiles spatially resolved directly on the paraffin-embedded tissue to depict and assign to the histological and clinical pathological subtypes of cancer.

Here, we have applied the method to detect a molecular signature of 13 peptides that predicts tumor recurrence in patients with early-stage HGSOC. According to their specific sequence, these peptides were allocated to a signature of proteins for risk stratification in support of clinical management of patients with early-stage HGSOC.

## 2. Results

### 2.1. Accumulation of Proteomics Data by MALDI-IMS

**Figure 1:**
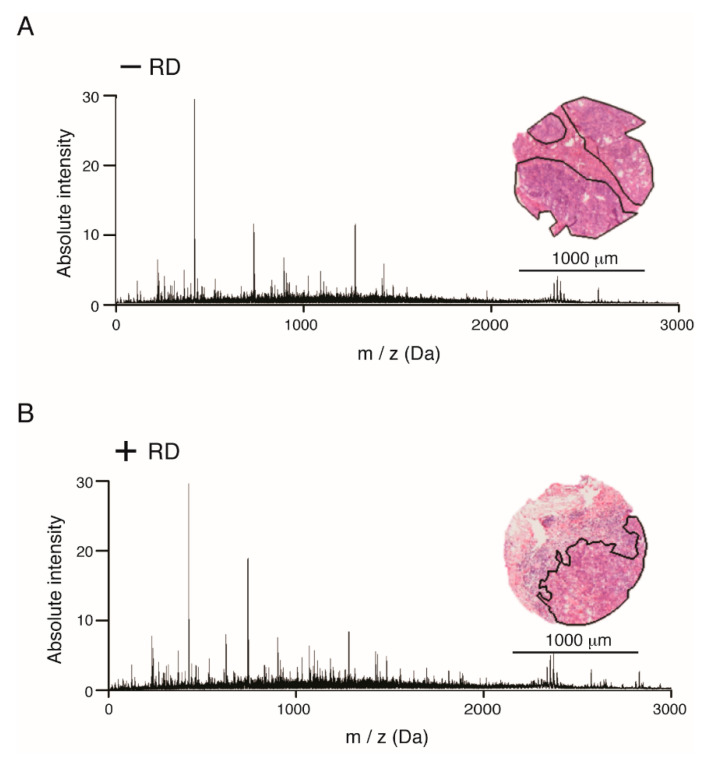
Average spectra of representative MALDI-imaging proteomic profiles of primary tumor sections from either (**A**) patients without or (**B**) with recurrent disease. For each group, examples of H&E images with indicated malignant areas measured are included. In total, 506 *m*/*z* values in a mass range between *m*/*z* 600 and 3000 (signal/noise > 1) were extracted by peak picking from high-grade serous ovarian cancer (HGSOC) at early-stage human tissue. Analyses were performed with 20 biologically independent spots (N = 10 each patient group).

The initial proteomic measurements were simultaneously carried out on primary tumor tissue sections of early-stage HGSOC patients (*n* = 10) with either recurrent disease (RD) or non-recurrent disease (non-RD), respectively. Mass spectra of primary tumor tissue sections of early-stage HGSOC were extracted and statistical data analysis was performed by the SCiLS Lab software. In total, 506 aligned *m*/*z* values in a mass range between *m*/*z* 600 and 3.000 were extracted (Table S1). Average spectra of primary tumor tissue sections of early-stage HGSOC are shown in Figure 1. The unsupervised data analysis of the peptide signatures by probabilistic latent semantic analysis (pLSA) allowed the discrimination of different patient groups via individual mass spectra compound intensity and spatial distribution. Analysis of the peptide signatures by pLSA resulted in the discriminative compounds for HGSOC patients with RD and non-RD. However, a third HGSOC patient group could be identified which showed individual pLSA compounds which did not match to patients with RD nor patients with non-RD (Figure S1).

Reassessment of the two patients in that subclass group by an experienced gynecological pathologist showed that the previous immunohistological expression pattern in one of the patient biopsies was not conclusive. As in high-grade serous ovarian carcinoma, p53 showed a mutated pattern, but unlike typical high-grade serous carcinoma, CD56 and synaptophysin expressions were evenly and strongly present. Moreover, the morphological picture indicates most likely an undifferentiated non-small cell neuroendocrine carcinoma (NSCNEC) of the ovary. The second patient was re-classified as pT2cG3 and hence not a HGSOC patient diagnosed at early stage. Therefore, samples of these two outlier patients were not considered for further analysis.

Since considerable differences in stroma content occur within the sample cohort, malignant compartments were evaluated in each core of early-stage HGSOC patients (N = 8). Mass spectra of malignant areas from both annotated groups (N = 4, each group) were obtained and a statistical comparison was performed using the SCiLS Lab software. In total, 612 *m*/*z* values from a mass range between *m*/*z* 800 and 3.500 (threshold 31.42) were identified by peak-picking and used to compare the tissue sections. Average exemplary spectra are shown for primary tumors of early-stage HGSOC patients with RD and non-RD in Figure 1. In total, MALDI-IMS analysis resulted in 7537 spectra from the entire patient cohort.

### 2.2. Discovery of Discriminative Peptide Signatures

**Figure 2:**
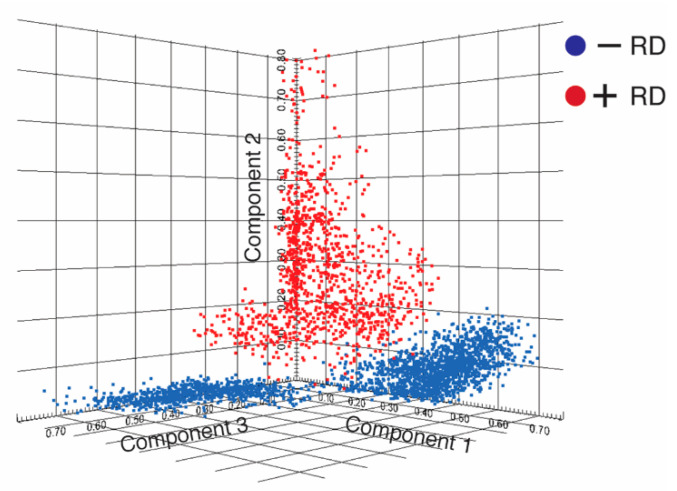
Discrimination of molecular signatures for the groups of HGSOC patients via probabilistic latent semantic analysis (pLSA). Score plots of the first three components from imaging mass spectrometry (IMS) spectra of primary tumors from patients without (−RD, in blue) and with recurrent disease (+RD, in red) are shown.

In order to determine specific molecular signatures in HGSOC patients with RD and non-RD, a pLSA based on the peptide signatures was performed and allowed the direct interpretation of score images and loadings. Here, this unsupervised data analysis of the peptide signature enabled the discrimination of both distinct patient groups (Figure 2).

### 2.3. Determination of Characteristic m/z Values of HGSOC Patients

**Figure 3:**
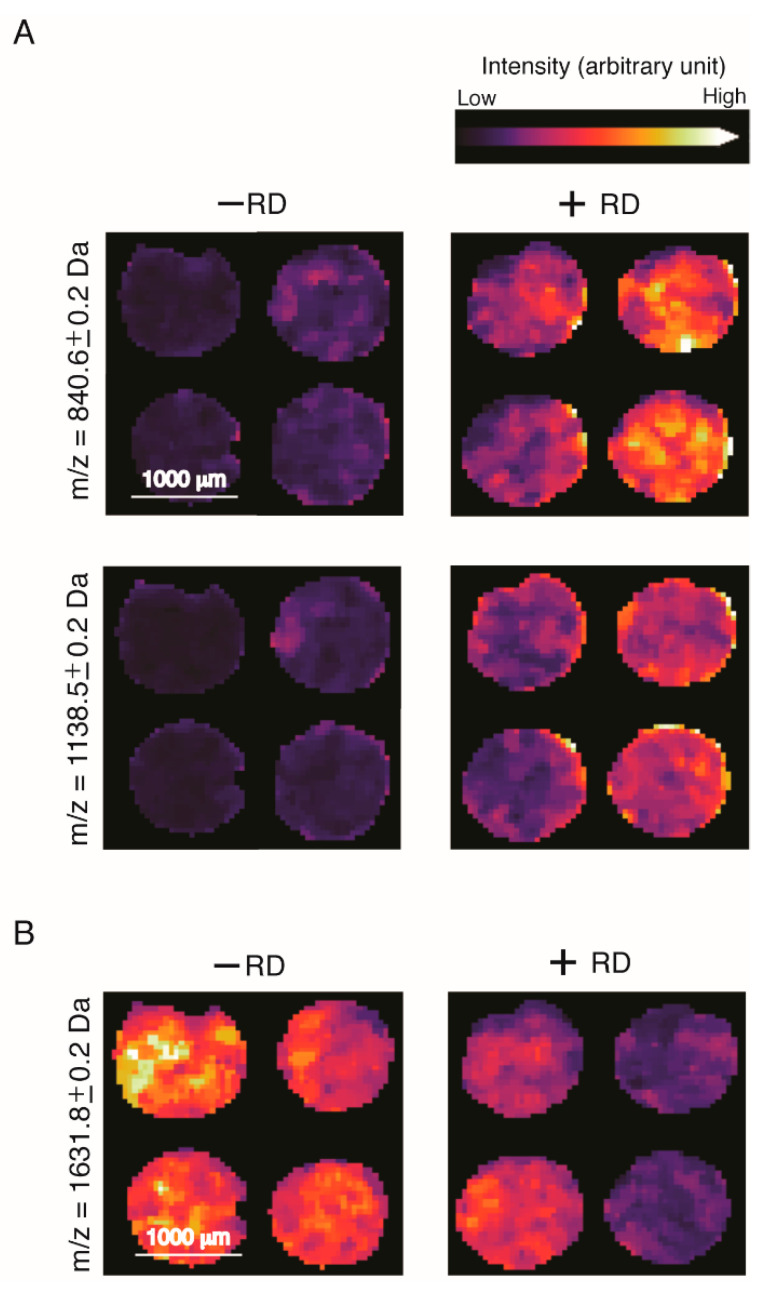
Characteristic peptides for group of patients with recurrence and no recurrence discrimination via individual peak mass spectra intensity and spatial peak distribution. (**A**) The *m*/*z* values 840.6 ± 0.2 and 1138.5 ± 0.2 Da show significantly higher spatial intensities (area under the curve (AUC) > 0.6; *p* < 0.001) in patients with recurrent disease (+RD) compared with without recurrence (−RD). (**B**) In contrast, the 1631.8 ± 0.2 Da peptide, as an example, exhibited significantly higher intensities (AUC < 0.4; *p* < 0.001) in patients with no recurrence.

The malignant compartments of the tumors were assigned and spectra were compared in a pairwise manner to obtain discriminative peptide values (*m*/*z*) using receiver operating characteristic (ROC) analysis. The ROC analysis resulted in 151 peptide values that were able to discriminate between patients with RD and non-RD (AUC > 0.6 or < 0.4; *p* < 0.01; Table S1). A selection is shown in Figure 3.

**Figure 4:**
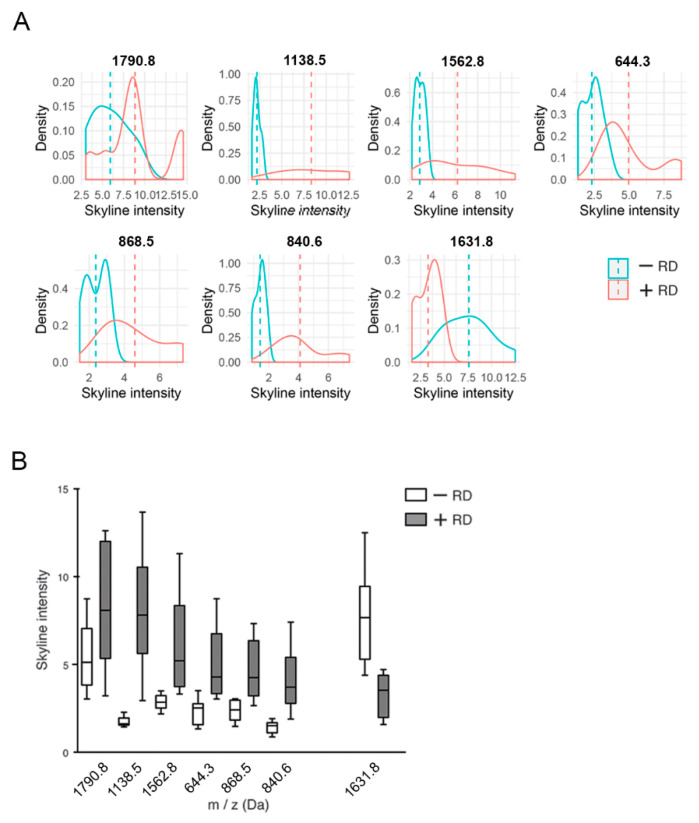
Examples of peptide distribution within groups of patients with (+RD) and without recurrent disease (−RD) by mass spectrometry intensity. (**A**) Density plot of skyline intensities over a continuous interval. Dashed lines indicate the distributions’ mean values. (**B**) Boxplot.

For example, the peptide values 840.6 ± 0.2 Da, 1138.5 ± 0.2 Da and 1631.8 ± 0.2 Da denote high spatial intensity distribution in patients with recurrence of tumors, which can be visualized as a heatmap distribution across the tissue section (Figure 3). The peptide value 1631.8 ± 0.2 was associated with non-RD. The distribution of the most significantly expressed peptides within the groups is shown in Figure 4.

### 2.4. Identification of Differentially Expressed Proteins

To improve the understanding of the disease progress and provide a method for personalized pathology assessment of early-stage HGSOC, specific localized peptide values were investigated and subsequently identified. Identification of these peptide markers provides important insights into the disease mechanism as well as progression. Since a large number of isobaric ions and the presence of so-called chimera spectra adversely affected the identification of *m*/*z* values by MS/MS (direct from tissue section), we performed a corresponding “bottom-up” LC-MS/MS approach (Table S2) with adjacent tissue sections, which enabled the identification of the obtained MALDI-IMS *m*/*z* values.

Out of the MADLI-IMS-derived discriminative *m*/*z* values between RD and non-RD HGSOC, 18 *m*/*z* values could be assigned to 13 proteins (Table 1).

More than one *m*/*z* value with similar discrimination characteristics was identified from Keratin type 1 and Collagen alpha-2(I) and was assigned to the observed *m*/*z* values from the MALDI-IMS experiment, hence correctly recognized (Table 1).

### 2.5. Relatedness between Patients with RD and between Patients without RD

**Figure 5:**
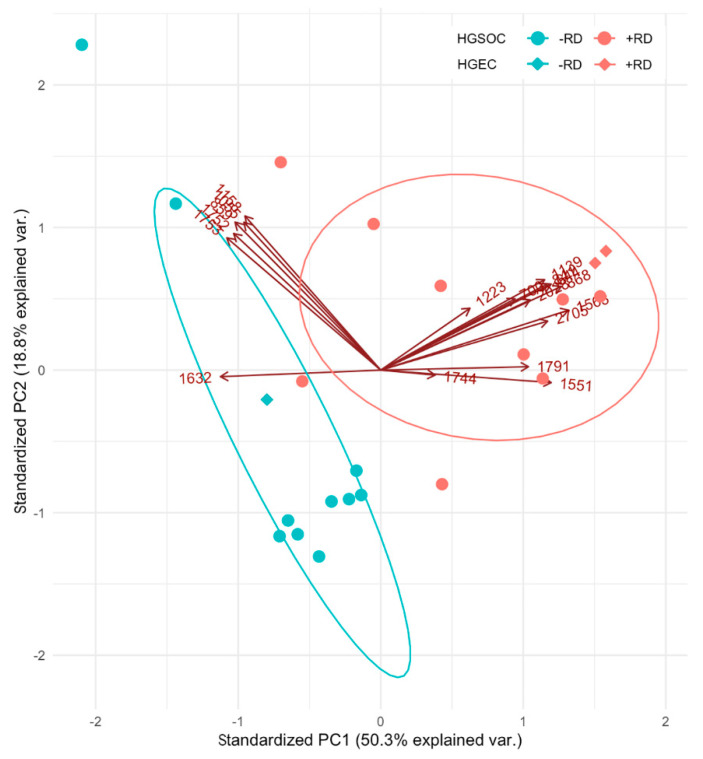
A biplot showing included samples of early-stage HGSOC patients as points. Additionally, three patients with high-grade endometrioid ovarian cancer (HGEC) were included in the analysis and marked with diamonds. Biplot axes indicate the influence of each peptide in the principal component space. The principal component analysis (PCA) shows a discrimination of patients with (+RD) and without recurrent disease (−RD).

The analysis was expanded and the peptide signature (discriminative *m*/*z* values) was applied to three additional early-stage patients with high-grade endometrioid ovarian cancer (HGEC), two of them with RD; one non-RD, and showed comparable peptide intensities in samples of HGSOC patients. A principal component analysis (PCA) was performed overlaying covariate influences onto the principal component space (Figure 5).

PCA confirmed the closer relatedness between patients with RD and between patients without RD. Inclusion of three early-stage HGEC patients showed similar relatedness. The variable markers cluster in two groups indicating correlated variables. The higher correlated group A comprises 1157.7 ± 0.2, 858.6 ± 0.2, 1751.8±0.2, 1753.0 ± 0.2 and 1055.4 ± 0.2 Da. The less correlated group B comprises the remaining peptides with 1631.8 ± 0.2 Da being negatively correlated (Figure 4A,B). The high proportion of variability explained by the two-dimensional principal subspace provides solid grounds for these correlations.

## 3. Discussion

In general, HGSOC patients diagnosed at early-stage have an excellent prognosis and concern arises that some of the early-stage HGSOCs are over-treated. Hence, there has been a debate about the optimal duration and chemotherapy treatment strategy, e.g., Carboplatin only, or combination regimens, four cycles vs. six cycles. However, a subgroup of patients will relapse and need therapies that are more intensive at time of diagnosis. It is therefore of great importance to identify these high-risk patients in order to improve their clinical outcome.

Currently, there are no reliable markers at hand for standard immunohistochemical assessment of this subpopulation. Here, we have used a novel approach using MALDI-IMS technology to screen for a prognostic peptide signature to support the clinical management of these patients. For this purpose, standardized protocols for MALDI-IMS sample preparation have been developed [25,26], which are intended to enable reliable exploration of molecular signatures as biomarkers and has been shown to provide valuable diagnostic and risk assessment capabilities for other diagnostically challenging neoplasms [27]. Our recently published data, showed that IMS can reliably detect the histological subtypes of ovarian cancer [20]. In this presented study, proteomic analysis results 151 discriminative *m*/*z* values between early-stage HGSOC patients with either RD or non-RD. In order to identify MALDI-IMS-derived *m*/*z* values, the “bottom-up”-nano liquid chromatography (nLC)-MS/MS approach was performed on adjacent tissue sections. According to the IMS guidelines [28], the mass difference between MALDI-IMS and LC-MS/MS *m*/*z* values should be less than 0.9 Da and requires the identification of more than one peptide.

Specific peptides linked to Keratin type1, Actin, cytoplasmic 1 and Collagen alpha-2(I) were observed to have the strongest expression levels in primary tumors from early-stage HGSOC patients with RD and indicated greatest prognostic values (AUC > 0.7). A published reference database of MALDI-IMS-derived peptide and protein values in various and in particular for ovarian cancer FFPE tissue [29] was intended as support for the verification of protein identifications. The observed *m*/*z* values 1562.8 ± 0.2 from Collagen alpha-2(I) and 1790.9 ± 0.2 Da from Actin, cytoplasmic 1 were also determined and identified in MALDI-IMS studies of biopsies from lung tumor patients [30]. Through regulation of various signaling pathways in cancer cells, Keratins, the epithelial-predominant members of the intermediate filament superfamily, are involved in a number of processes in tumor progression [31]. KRT9 is one of the most common contaminants in proteomic mass spectrometry analyses, both in ESI and MALDI mass spectrometry methods (see also reference [32]. These contaminations may rarely have their source in the sample material (randomly distributed), but are more often introduced during sample preparation (e.g., contamination from the environment like dust in solvents, buffers or matrix) [32]. However, the difference with MALDI imaging experiments is that the *m*/*z* values can be represented spatially in the tissue, such that contaminations would be evenly distributed over the whole sample material. Therefore, the tissue microarrays (TMAs) are randomized and a control area outside the tissue is measured as a control to exclude such contamination. No peptides (Isotopic pattern) and salt adducts were detected in the control area. Only singles from alpha -Cyano-4-hydroxycinnamic acid matrix clusters could be found with no influence on the data evaluation.

Moreover, both patient groups’ cores were included and randomly distributed on the same cover slip. Therefore, it is unlikely to detect any significant differences. Furthermore, MALDI imaging experiments predominantly address structural proteins, such as ECM molecules, since methodically an enzymatic surface digestion of the tissue sections is performed. Excluding cytoskeleton proteins from the analysis would be premature, especially since KRT9 is a cellular component of the cytoskeleton, cytosol, extracellular region or membrane (see https://www.uniprot.org/uniprot/P35527—subcellular location). Furthermore, a query of the kmplot.com (https://kmplot.com/analysis/) database showed a significant decrease in overall survival (*p* < 0.0052) associated with high expression of KRT9 considering only stage I EOC including HGSOC (*p* < 0.0028) patients (Figure S2). Therefore, our KRT9 MALDI-IMS measurement is unlikely a result of contamination.

The major sources of collagen expression are stromal cells with increased collagen production and disposition in the stromal compartment has been shown to be associated with breast cancer development and progression [33,34]. Nevertheless, it was also demonstrated that expression of collagen by ovarian cancer cells, including Collagen alpha-2(I), could increase drug resistance by inhibiting the penetration of the drug into the cancer tissue as well as increase resistance to apoptosis [35].

The analysis of three additional early-stage HGEC patients (two with RD; one without RD) showed comparable measured peptide intensities to the HGSOC patients. A multivariate regression was not feasible due to an insufficient number of observations [36]. However, reduction in covariates (dimension reduction), such as in a PCA, showed the discriminative capacity of the proposed prognostic marker candidates for patients with early-stage of either HGEC or HGSOC (Figure 5). Peptide markers separated into two distinct groups based on the correlation between them.

Even though the utilized sample size of four patients for each group is not sufficient for clinical validation, the purpose of this proof of concept study is to identify prognostic marker candidates. Consequently, validation of applicability of the proposed prognostic marker candidates, including for endometrioid carcinomas, necessitates subsequent high-sample size follow-up studies.

Moreover, changes in the tumor microenvironment in response to malignant transformation have been neglected in the past and need to be considered as a suitable compartment for biomarker discovery. So far, a major limitation of dissecting the stromal signature has been a lack of suitable methods. IMS is able to provide spatial information of protein signatures in both compartments. Unfortunately, the quality of the adjacent stroma in the majority of cores from the tumor tissue was not suitable for further assessment but should be included and subject of future prognostic biomarker research for early-stage HGSOC patients.

Eventually, a profound understanding of the biology in early-stage HGSOC might result in a redefinition of high-risk early-stage EOC to develop novel therapeutic approaches. However, this will need molecular characterization supported by RNA-Seq and high-resolution proteomics data from micro-dissected malignant and adjacent stroma compartments. Nevertheless, the identification of the subpopulation of patients developing recurrent tumors is an unmet clinical need. Here, we show that MALDI-IMS technology has the potential to make a meaningful impact for risk assessment and, hence, patient outcome.

## 4. Materials and Methods

### 4.1. Clinicopathological Parameters of Patient Cohort

All samples were collected at Charité, Department for Gynecology at surgery after patients gave their informed consent. Sample collection was permitted by the local ethics committee of the Charité Medical University Berlin (AVD-No. 2004-000034) and conducted according to the Declaration of Helsinki. All patients were of white caucasian background and received an accurate staging via laparotomy, including lymph node sampling. Diagnosis of the early-stage of the high-grade serous subtype of EOC was confirmed by an experienced gynecological pathologist. Adjuvant chemotherapy regime was applied to all patients based on carboplatin in combination with paclitaxel. Detailed descriptions of clinicopathological parameters of patients are shown in Table 2.

### 4.2. Procedure of MALDI-Imaging

Tissue microarrays (TMAs) of formalin-fixed paraffin-embedded tissue of patients diagnosed at early-stage HGSOC were designed and prepared at the Institute of Pathology, Charité Medical University Berlin. For MALDI-imaging, a 6 µm section was prepared from a paraffin block on a microtome and transferred onto Indium-Tin-Oxide slides (Bruker Daltonik, Bremen, Germany) through decreasing concentrations of ethanol (modified by Caprioli et al.) [37] and antigen retrieval was performed (modified by Gustafsson et al.) [38]. Trypsin and matrix solutions (α-Cyano-4-hydroxycinnamic acid) were deposited by an automated spraying device (HTX Sprayer). An amount of 550 µL trypsin solution (20µg, 20mM ammonium bicarbonate) was applied onto the section. After tissue incubation (2 h at 50 °C; moist chamber), matrix solution (1 mL 7g/L α-cyano-4-hydroxycinnamic acid in 50% acetonitrile and 1% trifluoroacetic acid) was applied using a HTX Sprayer (75 °C, estimate cycle 1.80).

### 4.3. MALDI Imaging Analysis

Analyses were performed on 10 biologically independent cores of biopsies for each patient group. MALDI-IMS data acquisition was executed in reflector mode, detection range of *m*/*z* 800–3200, 500 laser shots per spot, sampling rate of 1.25 GS/s and raster width of 50 µm on Rapiflex MALDI-TOF/ using flexControl 3.0 and flexImaging 3.0 (Bruker Daltonik). External calibration was performed using a peptide calibration standard (Bruker Daltonik) and spectra processed in flexAnalysis 3.0 (Bruker Daltonik). In order to exclude potential contamination like sodium adducts or peptides, control areas outside the tissue were also analyzed. After MALDI-imaging experiments, the matrix was removed with 70% ethanol and the tissue sections were stained with hematoxylin and eosin (H&E) as histological overview staining [37].

### 4.4. Data Processing

Statistical data analysis was performed using the SCiLS Lab software (Version2015b, SCiLS GmbH, Bremen, Germany). MALDI-IMS raw data were imported into the SCiLS Lab software and converted to the SCiLS Lab file format. Simultaneous preprocessing of all data sets was carried out to ensure better comparability between the sample sets. Imported data were pre-processed by convolution baseline removal (width: 20) and total ion count (TIC) normalization. Segmentation pipelines as published previously were performed for peak-finding and alignment [19,39,40]. Peaks were selected using the orthogonal matching pursuit (OMP) algorithm [41] and top down segmentations were performed by bisecting k-means clustering, ±0.156 Da interval width, mean interval processing and medium smoothing strength [39,40,41]. For convolutional neural networks evaluation, raw data from region spots and *m*/*z* values were exported from SCiLS Lab SW as csv format. Two approaches based on different principles were performed: first, an unsupervised approach, probabilistic latent semantic analysis (pLSA), to discriminate both groups, and another supervised approach, receiver operating characteristic (ROC) analysis, to detect characteristic peptide values. To define common molecular features among the sample sets, unsupervised multivariate classification methods for mass spectra were applied: probabilistic latent semantic analysis (pLSA) was performed as previously described [42,43]. pLSA was performed with five components and the following settings: (i) interval width of ± 0.156 Da, and (ii) individual spectra and deterministic initialization. Receiver operating characteristic analysis (ROC) was used to assess the quality of all *m*/*z* values within specific ROIs to discriminate between recurrent and non-recurrent HGSOC tumor tissue. For this method, the number of spectra in the ROIs of both groups should be approximately the same. If that was not the case, 1500 randomly selected spectra per ROI/group were used. To determine statistical significance, discriminating *m*/*z* values (peaks) with an AUC < 0.35 or > 0.65 were subsequently analyzed using the Wilcoxon rank sum test. *m*/*z* values with delta peak intensities of >0.7 and <0.3 (*p* < 0.001) were assumed as potential markers. Figures were created using the SCiLS Lab software (Bruker, Bremen, Germany) and R packages “ggplot2” and “ggbiplot”.”

### 4.5. Identification of Peptides by “Bottom-Up”-Nhplc Mass Spectrometry

To identify *m*/*z* values, complementary protein identification was performed on adjacent tissue sections by a “bottom-up”-nano liquid chromatography (nLC)-MS/MS approach as published previously [19]. Briefly, tissue digestion (20 µg trypsin, 20 mM ammonium bicarbonate/acetonitrile 9:1) was performed via ImagePrep (Bruker Daltonik). Peptides for nUPLC-MS/MS analysis were extracted directly from adjacent tissue sections into 40 µL of 0.1% triflouroaceticacid (TFA; 15 min incubation at room temperature). Peptides were separated (60% acetonitrile/ in 0.1% formic acid) using an analytical UPLC System (Thermo Dionex Ultimate 3000, Acclaim PepMap RSLC C18 column 75µmx 15 cm; flow rate 200 nL/min, 70 min) and analyzed via Impact II (QTOF-MS, Bruker Daltonik). All raw spectra from the MS/MS measurement were converted to mascot generic files (.mgf) using the ProteinScape software [44]. Mass spectra were analyzed using the Mascot search engine (version 2.4, MatrixScience; UK) searching the UniPort database. The search was performed with the following set of parameters: (i) taxonomy: human; (ii) proteolytic enzyme: trypsin; (iii) peptide tolerance: 10ppm; (iv) maximum of accepted missed cleavages: 1; (v) peptide charge: 2+, 3+, 4+; (vi) variable modification: oxidation (M); (vii) MS/MS tolerance: 0.8Da; and (viii) MOWSE score > 25. Identification of MALDI-IMS *m*/*z* values by using an LC-MS/MS reference list requires the accordance of more than one peptide (mass differences <0.9 Da) to subsequently correctly assign the corresponding protein [45]. Peptides with lowest mass difference to the LC-MS/MS reference list value were assumed as a match.

## 5. Conclusions

Epithelial ovarian cancer (EOC) has the highest mortality rate of the gynecological malignancies worldwide, with HGSOC representing the most common and aggressive histological subtype. Even though HGSOC patients diagnosed at early-stage have an excellent prognosis, a subgroup of patients will relapse and need therapies that are more intensive at time of diagnosis. It is therefore of great importance to identify these high-risk patients in order to improve their clinical outcome. In this proof of concept study, we have applied a novel approach using MALDI-IMS technology to identify a candidate prognostic peptide signature to support the clinical management of these patients. However, there is still a need for a robust validation of our candidate signature based on a higher-size patient cohort that should be addressed in the future. This includes implementing the identified and validated prognostic peptide signature as part of prospective studies in the clinical routine.

## Figures and Tables

**Table 1:** Receiver operating characteristic (ROC) curve analysis reveals a prognostic protein signature for early-stage HGSOC. Significantly differentially expressed proteins in primary tumors of patients with recurrent compared with no-recurrent disease are listed (overexpressed, AUC values > 0.6, and underrepresented < 0.4, *p* < 0.0001).

Centroid [*m*/*z*]	IMS Mr [*m*/*z*] [Da]	Tumor +RD vs −RD (AUC)	LC-MS Mr [Da]	Δ [Da]	Ascension	Protein	HGNC Symbol
2705.026	2704.0181	0.7547	2704.1538	0.1358	K1C9_HUMAN	Keratin, type I cytoskeletal 9	KRT9
1791.698	1790.6901	0.6250	1790.7204	0.0304	K1C9_HUMAN	Keratin, type I cytoskeletal 9	KRT9
644.336	643.3281	0.7470	643.3653	0.0373	ACTB_HUMAN	Actin, cytoplasmic 1	ACTB
840.564	839.5561	0.7407	839.4613	0.0947	CO1A2_HUMAN	Collagen alpha-2(I) chain	COL1A2
868.467	867.4591	0.7331	867.4563	0.0028	CO1A2_HUMAN	Collagen alpha-2(I) chain	COL1A2
2027.831	2026.8231	0.7008	2026.0093	0.8138	CO1A2_HUMAN	Collagen alpha-2(I) chain	COL1A2
1562.765	1561.7571	0.6930	1561.7849	0.0278	CO1A2_HUMAN	Collagen alpha-2(I) chain	COL1A2
1223.417	1222.4091	0.6262	1222.6054	0.1964	CO1A2_HUMAN	Collagen alpha-2(I) chain	COL1A2
700.444	699.4361	0.6388	699.4643	0.0282	RL37A_HUMAN	60S ribosomal protein L37a	RPL37A
1790.797	1789.7891	0.6253	1789.8846	0.0956	ACTB_HUMAN	Actin, cytoplasmic 1	ACTB
1743.691	1742.6831	0.6055	1742.8120	0.1290	H2B1N_HUMAN	Histone H2B type 1-N	HIST1H2BN
1550.764	1549.7561	0.6016	1549.8100	0.0540	ANXA1_HUMAN	Annexin A1	ANXA1
858.566	857.5581	0.3975	857.4607	0.0974	CALD1_HUMAN	Caldesmon	CALD1
1157.708	1156.7001	0.3782	1156.6200	0.0800	APOA1_HUMAN	Apolipoprotein A-I	APOA1
1631.775	1630.7671	0.3682	1630.8236	0.0566	TBB5_HUMAN	Tubulin beta chain	TUBB
1751.792	1750.7841	0.3554	1750.0353	0.7488	H2B1K_HUMAN	Histone H2B type 1-K	HIST1H2BK
1055.394	1054.3861	0.3460	1054.5196	0.1335	4_HUMAN	Histone H4	HIST1H4A
1752.992	1751.9841	0.3159	1751.8551	0.1290	LMNA_HUMAN	Prelamin-A/C	LMNA

**Table 2:** Clinicopathological characteristics of patients. All patients received adjuvant chemotherapy for numbers of cycles as indicated in the table. Follow-ups of patients were performed for at least 5 years, if no relapse occurred, or till development of recurrent disease (RD).

**Patients (−RD)**				
Age	68	60	68	67
FIGO stage	IA	IC	IA	IC
Grade	G3	G2	G3	G3
Presence of ascites	<500 mL	<500 mL	<500 mL	no
Number of cycles	6	6	4	6
Recurrence (months)	NA	NA	NA	NA
**Patients (+RD)**				
Age	44	52	67	57
FIGO stage	IIB	IIA	IA	IIA
Grade	G3	G3	G3	G3
Presence of ascites	>500 mL	no	No	no
Number of cycles	6	9	6	6
Recurrence (months)	13	12	54	16

## Supplementary Materials

The following are available online at https://www.mdpi.com/2072-6694/12/8/2000/s1, Figure S1: Discrimination of molecular signatures for groups of HGSOC patients via probabilistic latent semantic analysis (pLSA), Figure S2: Kaplan–Meier curves displaying the estimated overall survival probability of EOC patients with regard KRT9 expression, Table S1: *m*/*z* values from IMS and the corresponding identification and AUC values, Table S2: LC-MS reference list of ovarian cancer tissue.
