# Supplementary material for: Discovery of Prognostic Markers for Early-Stage High-Grade Serous Ovarian Cancer by Maldi-Imaging
Source: Cancers (Basel). 2020 Jul 22;12(8):2000. doi: 10.3390/cancers12082000 (PMC7463791; doi:10.3390/cancers12082000)
Supplement: Supplementary file 1 [file cancers-12-02000-s001.pdf]

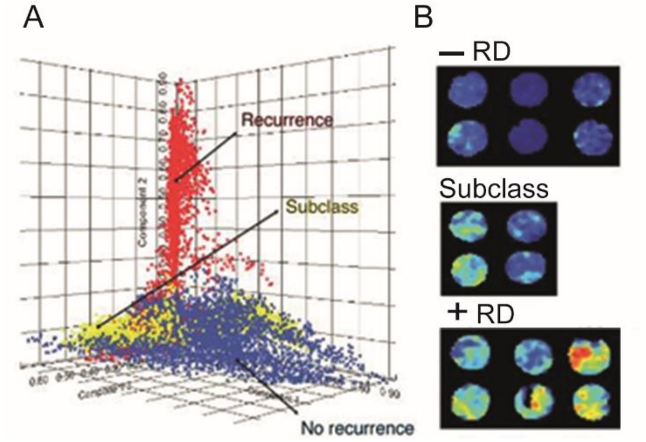

Figure S1. Discrimination of molecular signatures for groups of HGSOC patients via probabilistic latent semantic analysis (pLSA). (**A**) Score plots of the first three components from IMS spectra of primary tumors from patients without (- RD, in blue), a subclass (in yellow) and recurrent disease (+ RD, in red) are shown. (**B**) The intensity distribution of the component for the considered tissue sections, which clearly discriminates groups with high intensity (+RD), with low intensity (-RD) and intermediate subclass in component 2. Relative intensity scale: blue = low, yellow intermediate, red = high.

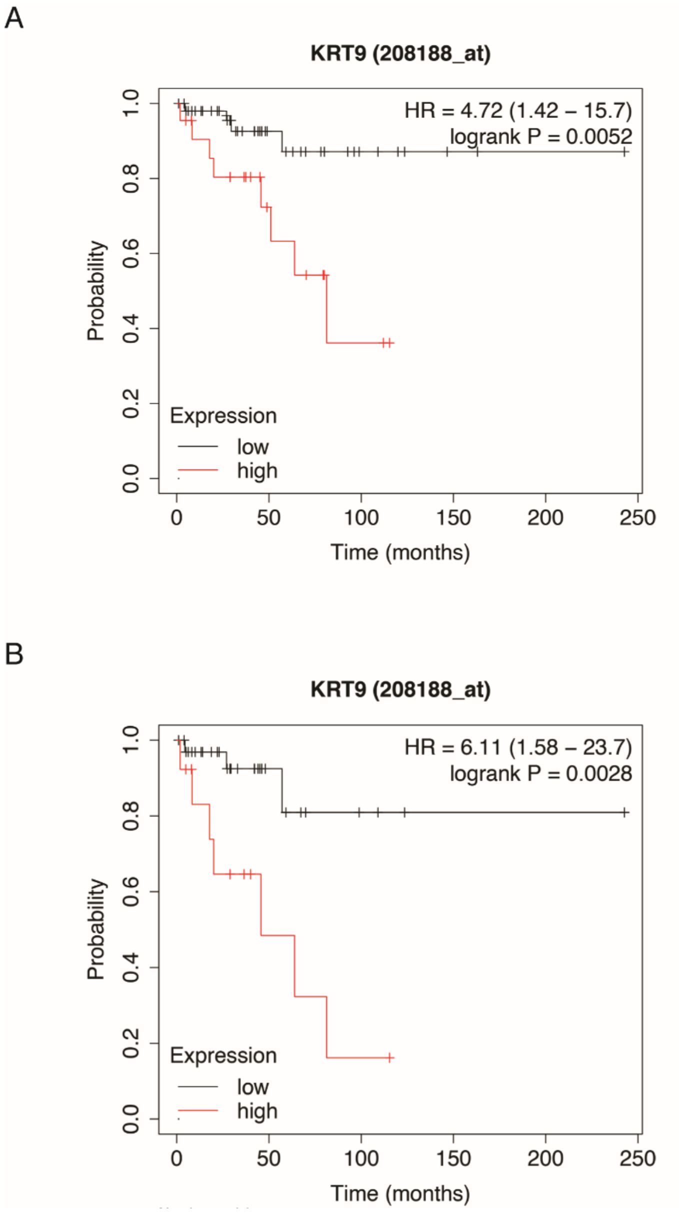

Figure 2. Kaplan-Meier curves displaying the estimated overall survival probability of two groups of either (**A**) early-stage EOC (*n* = 74) and (**B**) HGSOC (*n* = 47) patients alone. All patients were diagnosed at stage I of the disease. Patient groups were separated by https://kmplot.com/analysis/ default selection computing cutoffs between the lower and upper quartile of KRT9 (208188_at) expression.

Table 1. M/z values from IMS and the corresponding identification and AUC values.

Centroid [m/z]	IMS Mr [m/z][Da]	Tumor +RD vs -RD (AUC)	LC-MS Mr [Da]	Δ [Da]	Ascension	Proterin	Ascension
615.232	614.22406	0.555948	N/A	N/A	N/A	N/A	N/A
616.132	615.12406	0.563313	N/A	N/A	N/A	N/A	N/A
617.332	616.32406	0.480532	N/A	N/A	N/A	N/A	N/A
618.232	617.22406	0.616043	N/A	N/A	N/A	N/A	N/A
630.234	629.22606	0.536213	N/A	N/A	N/A	N/A	N/A
644.336	643.3281	0.7470	643.3653	0.0373	ACTB_HUMAN	Actin, cytoplasmic 1	ACTB
700.444	699.4361	0.6388	699.4643	0.0282	RL37A_HUMAN	60S ribosomal protein L37a	RPL37A
701.344	700.33606	0.405656	700.3868	0.05072794	VIME_HUMAN	Vimentin	VIM
702.244	701.23606	0.473686	N/A	N/A	N/A	N/A	N/A
715.446	714.43806	0.442839	N/A	N/A	N/A	N/A	N/A
760.452	759.44406	0.629369	N/A	N/A	N/A	N/A	N/A
785.356	784.34806	0.658677	N/A	N/A	N/A	N/A	N/A
786.556	785.54806	0.520624	N/A	N/A	N/A	N/A	N/A
788.356	787.34806	0.49249	787.4705	0.12239799	H31T_HUMAN	Histone H3.1t	HIST3H3
789.556	788.54806	0.506226	N/A	N/A	N/A	N/A	N/A
793.457	792.44906	0.43822	792.5082	0.05918048	H2A1C_HUMAN	Histone H2A type 1-C	HIST1H2AC
794.357	793.34906	0.448791	N/A	N/A	N/A	N/A	N/A
795.557	794.54906	0.559631	N/A	N/A	N/A	N/A	N/A
802.458	801.45006	0.480588	N/A	N/A	N/A	N/A	N/A
805.459	804.45106	0.539343	804.4494	0.0016718	CO6A3_HUMAN	Collagen alpha-3(VI) chain	COL6A3
815.36	814.35206	0.539004	N/A	N/A	N/A	N/A	N/A
817.46	816.45206	0.451789	N/A	N/A	N/A	N/A	N/A
831.562	830.55406	0.487251	830.4862	0.06789236	H31_HUMAN	Histone H3.1	HIST1H3A

840.564	839.5561	0.7407	839.4613	0.0947	CO1A2_HUMAN	Collagen alpha-2(I) chain	COL1A2
841.464	840.45606	0.695121	N/A	N/A	N/A	N/A	N/A
842.364	841.35606	0.546897	N/A	N/A	N/A	N/A	N/A
843.564	842.55606	0.529709	N/A	N/A	N/A	N/A	N/A
844.464	843.45606	0.507568	N/A	N/A	N/A	N/A	N/A
852.265	851.25706	0.685964	N/A	N/A	N/A	N/A	N/A
856.466	855.45806	0.518809	N/A	N/A	N/A	N/A	N/A
857.366	856.35806	0.352866	N/A	N/A	N/A	N/A	N/A
858.566	857.5581	0.3975	857.4607	0.0974	CALD1_HUMAN	Caldesmon	CALD1
868.467	867.4591	0.7331	867.4563	0.0028	CO1A2_HUMAN	Collagen alpha-2(I) chain	COL1A2
871.468	870.46006	0.449579	870.5035	0.04348903	H31T_HUMAN	Histone H3.1t	HIST3H3
872.368	871.36006	0.489503	871.4036	0.04350022	VIME_HUMAN	Vimentin	VIM
885.57	884.56206	0.451334	N/A	N/A	N/A	N/A	N/A
886.47	885.46206	0.561577	N/A	N/A	N/A	N/A	N/A
899.372	898.36406	0.646156	N/A	N/A	N/A	N/A	N/A
901.472	900.46406	0.472736	900.4930	0.02892435	H2B1K_HUMAN	Histone H2B type 1-K	HIST1H2BK
916.474	915.46606	0.426711	N/A	N/A	N/A	N/A	N/A
928.476	927.46806	0.402365	N/A	N/A	N/A	N/A	N/A
929.376	928.36806	0.397569	N/A	N/A	N/A	N/A	N/A
930.576	929.56806	0.436864	N/A	N/A	N/A	N/A	N/A
945.578	944.57006	0.399574	N/A	N/A	N/A	N/A	N/A
946.478	945.47006	0.432471	N/A	N/A	N/A	N/A	N/A
947.379	946.37106	0.482256	N/A	N/A	N/A	N/A	N/A
955.48	954.47206	0.452269	N/A	N/A	N/A	N/A	N/A
958.48	957.47206	0.438522	N/A	N/A	N/A	N/A	N/A

959.38	958.37206	0.433367	N/A	N/A	N/A	N/A	N/A
967.481	966.47306	0.468854	N/A	N/A	N/A	N/A	N/A
971.382	970.37406	0.506531	N/A	N/A	N/A	N/A	N/A
976.483	975.47506	0.568484	975.4410	0.03405164	ACTA_HUMAN	Actin, aortic smooth muscle	ACTA2
977.383	976.37506	0.569887	N/A	N/A	N/A	N/A	N/A
982.484	981.47606	0.512826	N/A	N/A	N/A	N/A	N/A
983.384	982.37606	0.502247	N/A	N/A	N/A	N/A	N/A
985.484	984.47606	0.496595	N/A	N/A	N/A	N/A	N/A
1020.589	1019.58106	0.601078	N/A	N/A	N/A	N/A	N/A
1027.49	1026.48206	0.444199	N/A	N/A	N/A	N/A	N/A
1028.69	1027.68206	0.435902	1027.5662	0.11585113	LMNA_HUMAN	Prelamin-A/C	LMNA
1029.59	1028.58206	0.453524	N/A	N/A	N/A	N/A	N/A
1032.591	1031.58306	0.587616	1031.5876	0.00455301	H31_HUMAN	Histone H3.1	HIST1H3A
1033.491	1032.48306	0.577186	N/A	N/A	N/A	N/A	N/A
1039.492	1038.48406	0.476467	1038.5862	0.10215603	TBB5_HUMAN	Tubulin beta chain	TUBB
1045.492	1044.48406	0.537965	N/A	N/A	N/A	N/A	N/A
1055.394	1054.3861	0.3460	1054.5196	0.1335	H4_HUMAN	Histone H4	HIST1H4A
1086.598	1085.59006	0.427334	N/A	N/A	N/A	N/A	N/A
1095.599	1094.59106	0.759168	N/A	N/A	N/A	N/A	N/A
1096.5	1095.49206	0.692059	N/A	N/A	N/A	N/A	N/A
1104.601	1103.59306	0.495827	N/A	N/A	N/A	N/A	N/A
1106.401	1105.39306	0.831044	N/A	N/A	N/A	N/A	N/A
1111.502	1110.49406	0.710924	N/A	N/A	N/A	N/A	N/A
1138.505	1137.49706	0.778966	N/A	N/A	N/A	N/A	N/A
1139.406	1138.39806	0.703354	N/A	N/A	N/A	N/A	N/A
1144.506	1143.49806	0.42887	N/A	N/A	N/A	N/A	N/A
1156.508	1155.50006	0.448723	N/A	N/A	N/A	N/A	N/A

1157.708	1156.7001	0.3782	1156.6200	0.0800	APOA1_HUMAN	Apolipoprotein A-I	APOA1
1162.509	1161.50106	0.55845	N/A	N/A	N/A	N/A	N/A
1167.61	1166.60206	0.442152	N/A	N/A	N/A	N/A	N/A
1180.511	1179.50306	0.503613	1179.6136	0.11049299	H4_HUMAN	Histone H4	HIST1H4A
1183.812	1182.80406	0.578471	N/A	N/A	N/A	N/A	N/A
1185.012	1184.00406	0.607598	N/A	N/A	N/A	N/A	N/A
1185.612	1184.60406	0.423457	N/A	N/A	N/A	N/A	N/A
1186.512	1185.50406	0.442117	1185.6394	0.13531381	IGHG1_HUMAN	Ig gamma-1 chain C region	IGHG1
1197.614	1196.60606	0.498442	N/A	N/A	N/A	N/A	N/A
1198.514	1197.50606	0.582634	N/A	N/A	N/A	N/A	N/A
1200.614	1199.60606	0.574695	N/A	N/A	N/A	N/A	N/A
1201.514	1200.50606	0.526725	N/A	N/A	N/A	N/A	N/A
1204.515	1203.50706	0.4772	N/A	N/A	N/A	N/A	N/A
1209.615	1208.60706	0.574205	N/A	N/A	N/A	N/A	N/A
1210.516	1209.50806	0.576054	N/A	N/A	N/A	N/A	N/A
1211.716	1210.70806	0.534348	N/A	N/A	N/A	N/A	N/A
1221.617	1220.60906	0.478172	N/A	N/A	N/A	N/A	N/A
1223.417	1222.4091	0.6262	1222.6054	0.1964	CO1A2_HUMAN	Collagen alpha-2(I) chain	COL1A2
1224.618	1223.61006	0.584374	N/A	N/A	N/A	N/A	N/A
1231.519	1230.51106	0.517803	N/A	N/A	N/A	N/A	N/A
1239.62	1238.61206	0.522224	N/A	N/A	N/A	N/A	N/A
1240.52	1239.51206	0.670499	N/A	N/A	N/A	N/A	N/A
1241.72	1240.71206	0.618918	N/A	N/A	N/A	N/A	N/A
1243.52	1242.51206	0.512113	N/A	N/A	N/A	N/A	N/A
1244.72	1243.71206	0.485334	N/A	N/A	N/A	N/A	N/A
1258.522	1257.51406	0.476703	N/A	N/A	N/A	N/A	N/A
1267.524	1266.51606	0.78371	N/A	N/A	N/A	N/A	N/A

1269.624	1268.61606	0.515776	N/A	N/A	N/A	N/A	N/A
1270.524	1269.51606	0.468029	N/A	N/A	N/A	N/A	N/A
1271.724	1270.71606	0.489336	N/A	N/A	N/A	N/A	N/A
1275.625	1274.61706	0.411428	N/A	N/A	N/A	N/A	N/A
1276.525	1275.51706	0.428988	N/A	N/A	N/A	N/A	N/A
1286.726	1285.71806	0.489848	1285.6667	0.0514088	IGHG1_HUMAN	Ig gamma-1 chain C region	IGHG1
1287.626	1286.61806	0.477354	N/A	N/A	N/A	N/A	N/A
1288.527	1287.51906	0.492842	N/A	N/A	N/A	N/A	N/A
1299.628	1298.62006	0.445224	N/A	N/A	N/A	N/A	N/A
1300.528	1299.52006	0.451664	1299.6783	0.15821852	H2A1C_HUMAN	Histone H2A type 1-C	HIST1H2AC
1301.729	1300.72106	0.478628	1300.6412	0.07989527	APOA1_HUMAN	Apolipoprotein A-I	APOA1
1305.629	1304.62106	0.556321	N/A	N/A	N/A	N/A	N/A
1313.73	1312.72206	0.444254	N/A	N/A	N/A	N/A	N/A
1320.631	1319.62306	0.617871	N/A	N/A	N/A	N/A	N/A
1325.732	1324.72406	0.458951	1324.7463	0.0222385	H4_HUMAN	Histone H4	HIST1H4A
1326.632	1325.62406	0.452922	1325.6901	0.06602913	H4_HUMAN	Histone H4	HIST1H4A
1327.532	1326.52406	0.466405	N/A	N/A	N/A	N/A	N/A
1328.732	1327.72406	0.476765	N/A	N/A	N/A	N/A	N/A
1329.632	1328.62406	0.469183	N/A	N/A	N/A	N/A	N/A
1334.733	1333.72506	0.452167	N/A	N/A	N/A	N/A	N/A
1335.633	1334.62506	0.432721	1334.6830	0.05796954	H31_HUMAN	Histone H3.1	HIST1H3A
1336.533	1335.52506	0.427734	N/A	N/A	N/A	N/A	N/A
1337.734	1336.72606	0.449143	N/A	N/A	N/A	N/A	N/A
1340.734	1339.72606	0.477358	1339.6044	0.12161513	ANXA5_HUMAN	Annexin A5	ANXA5

1341.634	1340.62606	0.456413	1340.6684	0.0423821<br>1	CALD1_HUMA<br>N	Caldesmon	CALD1
1342.534	1341.52606	0.454704	N/A	N/A	N/A	N/A	N/A
1347.635	1346.62706	0.397426	N/A	N/A	N/A	N/A	N/A
1348.535	1347.52706	0.465611	N/A	N/A	N/A	N/A	N/A
1349.735	1348.72706	0.465767	N/A	N/A	N/A	N/A	N/A
1350.635	1349.62706	0.492155	N/A	N/A	N/A	N/A	N/A
1353.636	1352.62806	0.503086	N/A	N/A	N/A	N/A	N/A
1355.736	1354.72806	0.440151	N/A	N/A	N/A	N/A	N/A
1361.737	1360.72906	0.481501	N/A	N/A	N/A	N/A	N/A
1362.637	1361.62906	0.491967	N/A	N/A	N/A	N/A	N/A
1363.537	1362.52906	0.490218	N/A	N/A	N/A	N/A	N/A
1364.737	1363.72906	0.519464	N/A	N/A	N/A	N/A	N/A
1378.539	1377.53106	0.488645	N/A	N/A	N/A	N/A	N/A
1383.64	1382.63206	0.488409	N/A	N/A	N/A	N/A	N/A
1384.54	1383.53206	0.473738	N/A	N/A	N/A	N/A	N/A
1391.741	1390.73306	0.404032	N/A	N/A	N/A	N/A	N/A
1393.541	1392.53306	0.535245	N/A	N/A	N/A	N/A	N/A
1394.742	1393.73406	0.521229	N/A	N/A	N/A	N/A	N/A
1398.642	1397.63406	0.482538	N/A	N/A	N/A	N/A	N/A
1399.842	1398.83406	0.482364	N/A	N/A	N/A	N/A	N/A
1406.743	1405.73506	0.399466	N/A	N/A	N/A	N/A	N/A
1407.643	1406.63506	0.424319	N/A	N/A	N/A	N/A	N/A
1408.544	1407.53606	0.573047	N/A	N/A	N/A	N/A	N/A
1409.744	1408.73606	0.546097	N/A	N/A	N/A	N/A	N/A
1410.644	1409.63606	0.528096	N/A	N/A	N/A	N/A	N/A
1412.744	1411.73606	0.501238	N/A	N/A	N/A	N/A	N/A
1413.644	1412.63606	0.478465	N/A	N/A	N/A	N/A	N/A
1424.746	1423.73806	0.507934	N/A	N/A	N/A	N/A	N/A
1427.746	1426.73806	0.472735	N/A	N/A	N/A	N/A	N/A
1429.547	1428.53906	0.476134	N/A	N/A	N/A	N/A	N/A

1430.747	1429.73906	0.502981	N/A	N/A	N/A	N/A	N/A
1431.647	1430.63906	0.538465	N/A	N/A	N/A	N/A	N/A
1459.551	1458.54306	0.834278	N/A	N/A	N/A	N/A	N/A
1461.651	1460.64306	0.686601	N/A	N/A	N/A	N/A	N/A
1465.552	1464.54406	0.733587	N/A	N/A	N/A	N/A	N/A
1467.652	1466.64406	0.586251	1465.7963	0.84777484	H4_HUMAN	Histone H4	HIST1H4A
1475.753	1474.74506	0.541114	N/A	N/A	N/A	N/A	N/A
1476.653	1475.64506	0.544077	N/A	N/A	N/A	N/A	N/A
1477.553	1476.54506	0.783307	N/A	N/A	N/A	N/A	N/A
1478.753	1477.74506	0.728788	N/A	N/A	N/A	N/A	N/A
1479.654	1478.64606	0.670581	N/A	N/A	N/A	N/A	N/A
1483.854	1482.84606	0.435276	N/A	N/A	N/A	N/A	N/A
1489.855	1488.84706	0.589803	N/A	N/A	N/A	N/A	N/A
1490.755	1489.74706	0.55922	N/A	N/A	N/A	N/A	N/A
1491.655	1490.64706	0.526352	N/A	N/A	N/A	N/A	N/A
1493.755	1492.74706	0.530883	N/A	N/A	N/A	N/A	N/A
1494.656	1493.64806	0.502819	N/A	N/A	N/A	N/A	N/A
1495.556	1494.54806	0.498475	N/A	N/A	N/A	N/A	N/A
1496.756	1495.74806	0.474126	N/A	N/A	N/A	N/A	N/A
1497.656	1496.64806	0.499308	N/A	N/A	N/A	N/A	N/A
1501.557	1500.54906	0.570786	N/A	N/A	N/A	N/A	N/A
1504.857	1503.84906	0.52838	N/A	N/A	N/A	N/A	N/A
1505.757	1504.74906	0.515521	N/A	N/A	N/A	N/A	N/A
1506.657	1505.64906	0.492473	N/A	N/A	N/A	N/A	N/A
1508.758	1507.75006	0.510348	1507.8358	0.08578179	H2B1K_HUMAN	Histone H2B type 1-K	HIST1H2BK
1515.659	1514.65106	0.619808	N/A	N/A	N/A	N/A	N/A
1516.559	1515.55106	0.621006	N/A	N/A	N/A	N/A	N/A
1517.759	1516.75106	0.517315	N/A	N/A	N/A	N/A	N/A
1523.76	1522.75206	0.443386	N/A	N/A	N/A	N/A	N/A

1524.66	1523.65206	0.433341	N/A	N/A	N/A	N/A	N/A
1530.661	1529.65306	0.676566	N/A	N/A	N/A	N/A	N/A
1531.561	1530.55306	0.655896	N/A	N/A	N/A	N/A	N/A
1533.661	1532.65306	0.580134	N/A	N/A	N/A	N/A	N/A
1534.561	1533.55306	0.610978	N/A	N/A	N/A	N/A	N/A
1550.764	1549.7561	0.6016	1549.8100	0.0540	ANXA1_HUMAN	Annexin A1	ANXA1
1551.664	1550.65606	0.60171	N/A	N/A	N/A	N/A	N/A
1552.864	1551.85606	0.547196	N/A	N/A	N/A	N/A	N/A
1553.764	1552.75606	0.574308	N/A	N/A	N/A	N/A	N/A
1554.664	1553.65606	0.518175	N/A	N/A	N/A	N/A	N/A
1556.764	1555.75606	0.445137	N/A	N/A	N/A	N/A	N/A
1562.765	1561.7571	0.6930	1561.7849	0.0278	CO1A2_HUMAN	Collagen alpha-2(I) chain	COL1A2
1563.665	1562.65706	0.684835	N/A	N/A	N/A	N/A	N/A
1565.766	1564.75806	0.503933	N/A	N/A	N/A	N/A	N/A
1566.666	1565.65806	0.476319	1565.7434	0.08533797	LMNA_HUMAN	Prelamin-A/C	LMNA
1567.866	1566.85806	0.468125	N/A	N/A	N/A	N/A	N/A
1571.767	1570.75906	0.544188	N/A	N/A	N/A	N/A	N/A
1579.868	1578.86006	0.472989	N/A	N/A	N/A	N/A	N/A
1585.568	1584.56006	0.733703	N/A	N/A	N/A	N/A	N/A
1586.769	1585.76106	0.645064	N/A	N/A	N/A	N/A	N/A
1587.669	1586.66106	0.616882	N/A	N/A	N/A	N/A	N/A
1589.769	1588.76106	0.471025	N/A	N/A	N/A	N/A	N/A
1590.669	1589.66106	0.461943	N/A	N/A	N/A	N/A	N/A
1591.869	1590.86106	0.465484	N/A	N/A	N/A	N/A	N/A
1601.771	1600.76306	0.454773	N/A	N/A	N/A	N/A	N/A
1608.672	1607.66406	0.571252	N/A	N/A	N/A	N/A	N/A
1612.872	1611.86406	0.409474	N/A	N/A	N/A	N/A	N/A
1613.772	1612.76406	0.414679	N/A	N/A	N/A	N/A	N/A

1614.673	1613.66506	0.456722	N/A	N/A	N/A	N/A	N/A
1615.873	1614.86506	0.469952	N/A	N/A	N/A	N/A	N/A
1616.773	1615.76506	0.492324	N/A	N/A	N/A	N/A	N/A
1620.673	1619.66506	0.558872	N/A	N/A	N/A	N/A	N/A
1621.874	1620.86606	0.524601	N/A	N/A	N/A	N/A	N/A
1622.774	1621.76606	0.497823	N/A	N/A	N/A	N/A	N/A
1623.674	1622.66606	0.50507	N/A	N/A	N/A	N/A	N/A
1625.774	1624.76606	0.498656	N/A	N/A	N/A	N/A	N/A
1626.674	1625.66606	0.524907	N/A	N/A	N/A	N/A	N/A
1627.874	1626.86606	0.462659	N/A	N/A	N/A	N/A	N/A
1628.775	1627.76706	0.283754	N/A	N/A	N/A	N/A	N/A
1629.675	1628.66706	0.277512	N/A	N/A	N/A	N/A	N/A
1630.575	1629.56706	0.286143	N/A	N/A	N/A	N/A	N/A
1631.775	1630.7671	0.3682	1630.8236	0.0566	TBB5_HUMAN	Tubulin beta chain	TUBB
1632.675	1631.66706	0.422339	N/A	N/A	N/A	N/A	N/A
1636.876	1635.86806	0.495126	N/A	N/A	N/A	N/A	N/A
1637.776	1636.76806	0.527445	N/A	N/A	N/A	N/A	N/A
1644.677	1643.66906	0.476463	N/A	N/A	N/A	N/A	N/A
1649.778	1648.77006	0.528122	N/A	N/A	N/A	N/A	N/A
1650.678	1649.67006	0.515837	N/A	N/A	N/A	N/A	N/A
1652.778	1651.77006	0.507684	N/A	N/A	N/A	N/A	N/A
1654.878	1653.87006	0.410452	N/A	N/A	N/A	N/A	N/A
1655.778	1654.77006	0.629892	N/A	N/A	N/A	N/A	N/A
1656.678	1655.67006	0.696229	N/A	N/A	N/A	N/A	N/A
1657.879	1656.87106	0.535207	N/A	N/A	N/A	N/A	N/A
1670.781	1669.77306	0.534009	N/A	N/A	N/A	N/A	N/A
1676.781	1675.77306	0.49671	N/A	N/A	N/A	N/A	N/A
1682.782	1681.77406	0.484703	N/A	N/A	N/A	N/A	N/A
1685.783	1684.77506	0.58198	N/A	N/A	N/A	N/A	N/A
1686.683	1685.67506	0.606985	N/A	N/A	N/A	N/A	N/A
1691.783	1690.77506	0.500221	N/A	N/A	N/A	N/A	N/A

1692.684	1691.67606	0.510632	N/A	N/A	N/A	N/A	N/A
1693.884	1692.87606	0.504309	N/A	N/A	N/A	N/A	N/A
1694.784	1693.77606	0.508391	N/A	N/A	N/A	N/A	N/A
1696.884	1695.87606	0.452522	N/A	N/A	N/A	N/A	N/A
1701.685	1700.67706	0.523435	N/A	N/A	N/A	N/A	N/A
1703.785	1702.77706	0.477129	N/A	N/A	N/A	N/A	N/A
1704.685	1703.67706	0.513518	1703.8941	0.21708453	ANXA5_HUMAN	Annexin A5	ANXA5
1706.786	1705.77806	0.557157	N/A	N/A	N/A	N/A	N/A
1707.686	1706.67806	0.591912	N/A	N/A	N/A	N/A	N/A
1708.886	1707.87806	0.507967	N/A	N/A	N/A	N/A	N/A
1710.686	1709.67806	0.567988	N/A	N/A	N/A	N/A	N/A
1711.886	1710.87806	0.502941	N/A	N/A	N/A	N/A	N/A
1715.787	1714.77906	0.452244	N/A	N/A	N/A	N/A	N/A
1718.787	1717.77906	0.337904	N/A	N/A	N/A	N/A	N/A
1719.987	1718.97906	0.352569	N/A	N/A	N/A	N/A	N/A
1720.888	1719.88006	0.403971	N/A	N/A	N/A	N/A	N/A
1721.788	1720.78006	0.45965	N/A	N/A	N/A	N/A	N/A
1727.789	1726.78106	0.501965	N/A	N/A	N/A	N/A	N/A
1743.691	1742.6831	0.6055	1742.8120	0.1290	H2B1N_HUMAN	Histone H2B type 1-N	HIST1H2BN
1744.591	1743.58306	0.604124	N/A	#VALUE!	N/A	N/A	N/A
1745.791	1744.78306	0.581076	N/A	#VALUE!	N/A	N/A	N/A
1746.691	1745.68306	0.573036	N/A	#VALUE!	N/A	N/A	N/A
1748.792	1747.78406	0.49163	N/A	#VALUE!	N/A	N/A	N/A
1749.692	1748.68406	0.577907	N/A	#VALUE!	N/A	N/A	N/A
1750.892	1749.88406	0.308303	N/A	N/A	N/A	N/A	N/A
1751.792	1750.7841	0.3554	1750.0353	0.7488	H2B1K_HUMAN	Histone H2B type 1-K	HIST1H2BK
1752.992	1751.9841	0.3159	1751.8551	0.1290	LMNA_HUMAN	Prelamin-A/C	LMNA

1753.892	1752.88406	0.390873	N/A	N/A	N/A	N/A	N/A
1762.893	1761.88506	0.485372	N/A	N/A	N/A	N/A	N/A
1763.794	1762.78606	0.497095	N/A	N/A	N/A	N/A	N/A
1764.694	1763.68606	0.5225	N/A	N/A	N/A	N/A	N/A
1772.795	1771.78706	0.544523	N/A	N/A	N/A	N/A	N/A
1773.695	1772.68706	0.550449	N/A	N/A	N/A	N/A	N/A
1774.895	1773.88706	0.520941	N/A	N/A	N/A	N/A	N/A
1775.795	1774.78706	0.574886	1774.8018	0.01478158	H2B1C_HUMAN	Histone H2B type 1-C/E/F/G/I	HIST1H2BC
1776.695	1775.68706	0.572284	N/A	#VALUE!	N/A	N/A	N/A
1778.796	1777.78806	0.51423	N/A	#VALUE!	N/A	N/A	N/A
1790.797	1789.7891	0.6253	1789.8846	0.0956	ACTB_HUMAN	Actin, cytoplasmic 1	ACTB
1791.698	1790.6901	0.6250	1790.7204	0.0304	K1C9_HUMAN	Keratin, type I cytoskeletal 9	KRT9
1792.898	1791.89006	0.577407	N/A	#VALUE!	N/A	N/A	N/A
1793.798	1792.79006	0.560971	1792.9319	0.14186702	H2B1C_HUMAN	Histone H2B type 1-C/E/F/G/I	HIST1H2BC
1808.8	1807.79206	0.442793	N/A	N/A	N/A	N/A	N/A
1817.801	1816.79306	0.53392	N/A	N/A	N/A	N/A	N/A
1818.701	1817.69306	0.541914	N/A	N/A	N/A	N/A	N/A
1819.901	1818.89306	0.542296	N/A	N/A	N/A	N/A	N/A
1820.802	1819.79406	0.593348	N/A	N/A	N/A	N/A	N/A
1821.702	1820.69406	0.57802	N/A	N/A	N/A	N/A	N/A
1822.902	1821.89406	0.473182	N/A	N/A	N/A	N/A	N/A
1823.802	1822.79406	0.490128	N/A	N/A	N/A	N/A	N/A
1828.903	1827.89506	0.497071	N/A	N/A	N/A	N/A	N/A
1829.803	1828.79506	0.493628	N/A	N/A	N/A	N/A	N/A
1834.904	1833.89606	0.549378	N/A	N/A	N/A	N/A	N/A
1843.905	1842.89706	0.464476	N/A	N/A	N/A	N/A	N/A
1844.805	1843.79706	0.457172	N/A	N/A	N/A	N/A	N/A
1846.905	1845.89706	0.424585	N/A	N/A	N/A	N/A	N/A

1847.805	1846.79706	0.471293	N/A	N/A	N/A	N/A	N/A
1849.906	1848.89806	0.452563	N/A	N/A	N/A	N/A	N/A
1851.106	1850.09806	0.470681	N/A	N/A	N/A	N/A	N/A
1852.006	1850.99806	0.454471	N/A	N/A	N/A	N/A	N/A
1867.908	1866.90006	0.455126	N/A	N/A	N/A	N/A	N/A
1888.911	1887.90306	0.557962	N/A	N/A	N/A	N/A	N/A
1889.811	1888.80306	0.599149	N/A	N/A	N/A	N/A	N/A
1905.714	1904.70606	0.547187	N/A	N/A	N/A	N/A	N/A
1906.914	1905.90606	0.518219	N/A	N/A	N/A	N/A	N/A
1907.814	1906.80606	0.546609	N/A	N/A	N/A	N/A	N/A
1911.714	1910.70606	0.420728	N/A	N/A	N/A	N/A	N/A
1923.716	1922.70806	0.612843	N/A	N/A	N/A	N/A	N/A
1924.916	1923.90806	0.539657	N/A	N/A	N/A	N/A	N/A
1925.816	1924.80806	0.606697	N/A	N/A	N/A	N/A	N/A
1927.017	1926.00906	0.522037	N/A	N/A	N/A	N/A	N/A
1927.917	1926.90906	0.540851	N/A	N/A	N/A	N/A	N/A
1945.919	1944.91106	0.506751	N/A	N/A	N/A	N/A	N/A
1946.819	1945.81106	0.530527	N/A	N/A	N/A	N/A	N/A
1948.02	1947.01206	0.57796	N/A	N/A	N/A	N/A	N/A
1954.02	1953.01206	0.461566	N/A	N/A	N/A	N/A	N/A
1954.921	1953.91306	0.493088	1953.0571	0.8559324<br>7	ACTB_HUMAN	Actin, cytoplasmic 1	ACTB
1955.821	1954.81306	0.529218	N/A	N/A	N/A	N/A	N/A
1957.021	1956.01306	0.509865	N/A	N/A	N/A	N/A	N/A
1960.921	1959.91306	0.481032	N/A	N/A	N/A	N/A	N/A
1961.822	1960.81406	0.774316	N/A	N/A	N/A	N/A	N/A
1962.722	1961.71406	0.747956	N/A	N/A	N/A	N/A	N/A
1963.922	1962.91406	0.689502	N/A	N/A	N/A	N/A	N/A
1964.822	1963.81406	0.645173	N/A	N/A	N/A	N/A	N/A
1966.922	1965.91406	0.496503	N/A	N/A	N/A	N/A	N/A
1968.722	1967.71406	0.461815	N/A	N/A	N/A	N/A	N/A

1969.923	1968.91506	0.480778	N/A	N/A	N/A	N/A	N/A
1970.823	1969.81506	0.468167	N/A	N/A	N/A	N/A	N/A
1992.726	1991.71806	0.769079	N/A	N/A	N/A	N/A	N/A
1994.826	1993.81806	0.643603	N/A	N/A	N/A	N/A	N/A
1997.827	1996.81906	0.381092	N/A	N/A	N/A	N/A	N/A
1998.727	1997.71906	0.331067	N/A	N/A	N/A	N/A	N/A
1999.927	1998.91906	0.447166	N/A	N/A	N/A	N/A	N/A
2026.931	2025.92306	0.654135	N/A	N/A	N/A	N/A	N/A
2027.831	2026.8231	0.7008	2026.0093	0.8138	CO1A2_HUMAN	Collagen alpha-2(I) chain	COL1A2
2041.033	2040.02506	0.625153	N/A	N/A	N/A	N/A	N/A
2041.933	2040.92506	0.614802	N/A	N/A	N/A	N/A	N/A
2042.833	2041.82506	0.563426	N/A	N/A	N/A	N/A	N/A
2048.834	2047.82606	0.486737	N/A	N/A	N/A	N/A	N/A
2050.034	2049.02606	0.36238	N/A	N/A	N/A	N/A	N/A
2050.934	2049.92606	0.405206	N/A	N/A	N/A	N/A	N/A
2056.035	2055.02706	0.675156	N/A	N/A	N/A	N/A	N/A
2056.935	2055.92706	0.771859	N/A	N/A	N/A	N/A	N/A
2057.835	2056.82706	0.780259	N/A	N/A	N/A	N/A	N/A
2059.035	2058.02706	0.655057	N/A	N/A	N/A	N/A	N/A
2071.937	2070.92906	0.705197	N/A	N/A	N/A	N/A	N/A
2072.837	2071.82906	0.717666	N/A	N/A	N/A	N/A	N/A
2074.037	2073.02906	0.627961	N/A	N/A	N/A	N/A	N/A
2081.838	2080.83006	0.517692	N/A	N/A	N/A	N/A	N/A
2089.939	2088.93106	0.59346	N/A	N/A	N/A	N/A	N/A
2104.942	2103.93406	0.572789	N/A	N/A	N/A	N/A	N/A
2106.142	2105.13406	0.535522	N/A	N/A	N/A	N/A	N/A
2107.942	2106.93406	0.58095	N/A	N/A	N/A	N/A	N/A
2110.042	2109.03406	0.524806	N/A	N/A	N/A	N/A	N/A
2110.942	2109.93406	0.495352	N/A	N/A	N/A	N/A	N/A
2111.843	2110.83506	0.485072	N/A	N/A	N/A	N/A	N/A

2114.843	2113.83506	0.716855	N/A	N/A	N/A	N/A	N/A
2116.043	2115.03506	0.723549	N/A	N/A	N/A	N/A	N/A
2116.943	2115.93506	0.667872	N/A	N/A	N/A	N/A	N/A
2117.844	2116.83606	0.596979	N/A	N/A	N/A	N/A	N/A
2123.844	2122.83606	0.683351	N/A	N/A	N/A	N/A	N/A
2133.146	2132.13806	0.479645	N/A	N/A	N/A	N/A	N/A
2189.854	2188.84606	0.37258	N/A	N/A	N/A	N/A	N/A
2190.754	2189.74606	0.308271	N/A	N/A	N/A	N/A	N/A
2191.954	2190.94606	0.458379	2191.1637	0.21765787	CO6A3_HUMAN	Collagen alpha-3(VI) chain	COL6A3
2192.854	2191.84606	0.410341	N/A	N/A	N/A	N/A	N/A
2215.957	2214.94906	0.611124	N/A	N/A	N/A	N/A	N/A
2248.962	2247.95406	0.427922	N/A	N/A	N/A	N/A	N/A
2249.862	2248.85406	0.488021	N/A	N/A	N/A	N/A	N/A
2251.062	2250.05406	0.429955	N/A	N/A	N/A	N/A	N/A
2275.066	2274.05806	0.577622	N/A	N/A	N/A	N/A	N/A
2278.066	2277.05806	0.672096	N/A	N/A	N/A	N/A	N/A
2278.966	2277.95806	0.733161	N/A	N/A	N/A	N/A	N/A
2284.067	2283.05906	0.546475	N/A	N/A	N/A	N/A	N/A
2296.069	2295.06106	0.528781	N/A	N/A	N/A	N/A	N/A
2296.969	2295.96106	0.527833	N/A	N/A	N/A	N/A	N/A
2298.169	2297.16106	0.49048	N/A	N/A	N/A	N/A	N/A
2299.069	2298.06106	0.481686	N/A	N/A	N/A	N/A	N/A
2305.07	2304.06206	0.527063	N/A	N/A	N/A	N/A	N/A
2305.97	2304.96206	0.562465	N/A	N/A	N/A	N/A	N/A
2311.971	2310.96306	0.519066	N/A	N/A	N/A	N/A	N/A
2352.177	2351.16906	0.45606	2351.1717	0.00265637	ANXA1_HUMAN	Annexin A1	ANXA1
2353.077	2352.06906	0.458765	N/A	N/A	N/A	N/A	N/A
2368.979	2367.97106	0.56497	N/A	N/A	N/A	N/A	N/A
2370.179	2369.17106	0.539177	N/A	N/A	N/A	N/A	N/A

2410.085	2409.07706	0.603919	N/A	N/A	N/A	N/A	N/A
2410.985	2409.97706	0.651598	N/A	N/A	N/A	N/A	N/A
2416.085	2415.07706	0.504829	N/A	N/A	N/A	N/A	N/A
2416.986	2415.97806	0.519172	N/A	N/A	N/A	N/A	N/A
2420.886	2419.87806	0.733413	N/A	N/A	N/A	N/A	N/A
2422.987	2421.97906	0.630086	N/A	N/A	N/A	N/A	N/A
2423.886	2422.87806	0.58612	N/A	N/A	N/A	N/A	N/A
2430.188	2429.18006	0.423961	N/A	N/A	N/A	N/A	N/A
2431.088	2430.08006	0.480449	N/A	N/A	N/A	N/A	N/A
2431.988	2430.98006	0.555707	N/A	N/A	N/A	N/A	N/A
2437.088	2436.08006	0.593262	N/A	N/A	N/A	N/A	N/A
2437.989	2436.98106	0.58775	N/A	N/A	N/A	N/A	N/A
2438.889	2437.88106	0.547483	N/A	N/A	N/A	N/A	N/A
2440.089	2439.08106	0.541751	N/A	N/A	N/A	N/A	N/A
2440.989	2439.98106	0.559572	N/A	N/A	N/A	N/A	N/A
2445.19	2444.18206	0.457032	N/A	N/A	N/A	N/A	N/A
2451.19	2450.18206	0.526681	N/A	N/A	N/A	N/A	N/A
2452.091	2451.08306	0.546273	N/A	N/A	N/A	N/A	N/A
2452.991	2451.98306	0.556948	N/A	N/A	N/A	N/A	N/A
2454.191	2453.18306	0.563614	N/A	N/A	N/A	N/A	N/A
2455.091	2454.08306	0.632583	N/A	N/A	N/A	N/A	N/A
2455.991	2454.98306	0.656723	N/A	N/A	N/A	N/A	N/A
2460.192	2459.18406	0.435204	N/A	N/A	N/A	N/A	N/A
2461.092	2460.08406	0.47501	N/A	N/A	N/A	N/A	N/A
2466.193	2465.18506	0.558653	N/A	N/A	N/A	N/A	N/A
2467.093	2466.08506	0.574174	N/A	N/A	N/A	N/A	N/A
2467.993	2466.98506	0.59541	N/A	N/A	N/A	N/A	N/A
2469.193	2468.18506	0.553993	N/A	N/A	N/A	N/A	N/A
2470.093	2469.08506	0.655557	N/A	N/A	N/A	N/A	N/A
2470.993	2469.98506	0.687806	N/A	N/A	N/A	N/A	N/A
2472.193	2471.18506	0.426667	N/A	N/A	N/A	N/A	N/A

2473.094	2472.08606	0.494472	N/A	N/A	N/A	N/A	N/A
2474.294	2473.28606	0.361975	N/A	N/A	N/A	N/A	N/A
2475.194	2474.18606	0.3809	N/A	N/A	N/A	N/A	N/A
2476.094	2475.08606	0.400567	N/A	N/A	N/A	N/A	N/A
2477.294	2476.28606	0.396393	N/A	N/A	N/A	N/A	N/A
2478.194	2477.18606	0.431141	N/A	N/A	N/A	N/A	N/A
2482.995	2481.98706	0.531816	N/A	N/A	N/A	N/A	N/A
2487.196	2486.18806	0.421904	N/A	N/A	N/A	N/A	N/A
2488.096	2487.08806	0.53019	N/A	N/A	N/A	N/A	N/A
2489.296	2488.28806	0.432581	N/A	N/A	N/A	N/A	N/A
2490.196	2489.18806	0.423894	N/A	N/A	N/A	N/A	N/A
2491.396	2490.38806	0.445354	N/A	N/A	N/A	N/A	N/A
2492.296	2491.28806	0.421881	N/A	N/A	N/A	N/A	N/A
2493.196	2492.18806	0.45196	N/A	N/A	N/A	N/A	N/A
2494.096	2493.08806	0.637949	N/A	N/A	N/A	N/A	N/A
2494.997	2493.98906	0.728917	N/A	N/A	N/A	N/A	N/A
2497.997	2496.98906	0.582674	N/A	N/A	N/A	N/A	N/A
2500.097	2499.08906	0.48609	N/A	N/A	N/A	N/A	N/A
2503.398	2502.39006	0.522115	N/A	N/A	N/A	N/A	N/A
2504.298	2503.29006	0.530458	N/A	N/A	N/A	N/A	N/A
2505.198	2504.19006	0.517478	N/A	N/A	N/A	N/A	N/A
2506.398	2505.39006	0.503099	N/A	N/A	N/A	N/A	N/A
2507.298	2506.29006	0.488336	N/A	N/A	N/A	N/A	N/A
2508.198	2507.19006	0.540616	N/A	N/A	N/A	N/A	N/A
2509.099	2508.09106	0.646163	N/A	N/A	N/A	N/A	N/A
2509.999	2508.99106	0.673824	N/A	N/A	N/A	N/A	N/A
2517.2	2516.19206	0.536534	N/A	N/A	N/A	N/A	N/A
2518.4	2517.39206	0.56977	N/A	N/A	N/A	N/A	N/A
2519.3	2518.29206	0.547794	N/A	N/A	N/A	N/A	N/A
2520.2	2519.19206	0.560324	N/A	N/A	N/A	N/A	N/A
2521.1	2520.09206	0.651143	N/A	N/A	N/A	N/A	N/A

2522	2520.99206	0.685812	N/A	N/A	N/A	N/A	N/A
2523.201	2522.19306	0.537974	N/A	N/A	N/A	N/A	N/A
2534.302	2533.29406	0.492638	N/A	N/A	N/A	N/A	N/A
2548.104	2547.09606	0.456482	N/A	N/A	N/A	N/A	N/A
2584.109	2583.10106	0.526624	N/A	N/A	N/A	N/A	N/A
2647.118	2646.11006	0.574277	N/A	N/A	N/A	N/A	N/A
2648.018	2647.01006	0.597823	N/A	N/A	N/A	N/A	N/A
2649.218	2648.21006	0.578427	N/A	N/A	N/A	N/A	N/A
2650.118	2649.11006	0.595554	N/A	N/A	N/A	N/A	N/A
2663.02	2662.01206	0.602989	N/A	N/A	N/A	N/A	N/A
2663.92	2662.91206	0.599584	N/A	N/A	N/A	N/A	N/A
2665.121	2664.11306	0.586028	N/A	N/A	N/A	N/A	N/A
2666.021	2665.01306	0.601954	N/A	N/A	N/A	N/A	N/A
2666.921	2665.91306	0.590095	N/A	N/A	N/A	N/A	N/A
2668.121	2667.11306	0.578737	N/A	N/A	N/A	N/A	N/A
2677.122	2676.11406	0.616413	N/A	N/A	N/A	N/A	N/A
2678.022	2677.01406	0.610255	N/A	N/A	N/A	N/A	N/A
2678.923	2677.91506	0.601293	N/A	N/A	N/A	N/A	N/A
2680.123	2679.11506	0.573332	N/A	N/A	N/A	N/A	N/A
2692.124	2691.11606	0.655263	N/A	N/A	N/A	N/A	N/A
2693.024	2692.01606	0.636943	N/A	N/A	N/A	N/A	N/A
2695.125	2694.11706	0.566875	N/A	N/A	N/A	N/A	N/A
2704.126	2703.11806	0.707427	N/A	N/A	N/A	N/A	N/A
2705.026	2704.0181	0.7547	2704.1538	0.1358	K1C9_HUMAN	Keratin, type I cytoskeletal 9	KRT9
2705.926	2704.91806	0.740633	N/A	N/A	N/A	N/A	N/A
2707.126	2706.11806	0.679127	N/A	N/A	N/A	N/A	N/A
2720.028	2719.02006	0.772081	N/A	N/A	N/A	N/A	N/A
2720.928	2719.92006	0.748598	N/A	N/A	N/A	N/A	N/A
2722.129	2721.12106	0.651528	N/A	N/A	N/A	N/A	N/A
2727.229	2726.22106	0.708513	N/A	N/A	N/A	N/A	N/A

2728.129	2727.12106	0.786871	N/A	N/A	N/A	N/A	N/A
2729.03	2728.02206	0.778054	N/A	N/A	N/A	N/A	N/A
2749.132	2748.12406	0.624317	N/A	N/A	N/A	N/A	N/A
2750.032	2749.02406	0.630551	N/A	N/A	N/A	N/A	N/A
2798.039	2797.03106	0.604828	N/A	N/A	N/A	N/A	N/A
2799.24	2798.23206	0.561481	N/A	N/A	N/A	N/A	N/A
2800.14	2799.13206	0.586362	N/A	N/A	N/A	N/A	N/A
2801.04	2800.03206	0.577501	N/A	N/A	N/A	N/A	N/A
2867.049	2866.04106	0.65522	N/A	N/A	N/A	N/A	N/A
2868.249	2867.24106	0.609073	N/A	N/A	N/A	N/A	N/A
2869.149	2868.14106	0.835731	N/A	N/A	N/A	N/A	N/A
2870.05	2869.04206	0.857464	N/A	N/A	N/A	N/A	N/A
2871.25	2870.24206	0.821276	N/A	N/A	N/A	N/A	N/A
2872.15	2871.14206	0.807266	N/A	N/A	N/A	N/A	N/A
2881.151	2880.14306	0.79132	N/A	N/A	N/A	N/A	N/A
2882.051	2881.04306	0.769977	N/A	N/A	N/A	N/A	N/A
2883.251	2882.24306	0.699567	N/A	N/A	N/A	N/A	N/A
2884.151	2883.14306	0.70892	N/A	N/A	N/A	N/A	N/A
2885.052	2884.04406	0.783945	N/A	N/A	N/A	N/A	N/A
2886.252	2885.24406	0.762184	N/A	N/A	N/A	N/A	N/A
2887.152	2886.14406	0.790514	N/A	N/A	N/A	N/A	N/A
2916.256	2915.24806	0.597427	N/A	N/A	N/A	N/A	N/A
2959.162	2958.15406	0.760483	N/A	N/A	N/A	N/A	N/A
2960.062	2959.05406	0.797869	N/A	N/A	N/A	N/A	N/A
2961.262	2960.25406	0.706534	N/A	N/A	N/A	N/A	N/A
2962.162	2961.15406	0.7502	N/A	N/A	N/A	N/A	N/A

Table 2. LC-MS reference list of ovarian cancer tissue.

Row	Cmpd.	m/z meas.	Mr calc.	z	Δ m/z [ppm]	RMS90 [ppm]	Rt [min]	Scores	Accession	Protein
1	20787	303.1922	604.3697	2	0.17	4.87	25.15	25.1 (M.expect:0.0,M.score:25.1)	RL13_HUMAN	60S ribosomal protein L13 OS = Homo sapiens GN = RPL13 PE = 1 SV = 4
2	19813	307.7213	613.4275	2	0.71	1.95	12.32	42.0 (M.expect:0.0,M.score:42.0)	RL6_HUMAN	60S ribosomal protein L6 OS = Homo sapiens GN = RPL6 PE = 1 SV = 3
3	19579	312.1796	622.3439	2	1.39	36.57	18.06	31.8 (M.expect:0.0,M.score:31.8)	HV307_HUMAN	Immunoglobulin heavy variable 3-7 OS = Homo sapiens GN = IGHV3-7 PE = 1 SV = 2
4	19175	322.6897	643.3653	2	-0.77	3.88	18.12	25.0 (M.expect:0.1,M.score:25.0)	ACTA_HUMAN	Actin, aortic smooth muscle OS = Homo sapiens GN = ACTA2 PE = 1 SV = 1
5	19175	322.6897	643.3653	2	-0.77	3.88	18.12	25.0 (M.expect:0.1,M.score:25.0)	ACTBL_HUMAN	Beta-actin-like protein 2 OS = Homo sapiens GN = ACTBL2 PE = 1 SV = 2
6	19175	322.6897	643.3653	2	-0.77	3.88	18.12	25.0 (M.expect:0.1,M.score:25.0)	ACTB_HUMAN	Actin, cytoplasmic 1 OS = Homo sapiens GN = ACTB PE = 1 SV = 1
7	21005	322.7080	643.4017	2	-0.28	46.55	11.44	35.6 (M.expect:0.0,M.score:35.6)	WIZ_HUMAN	Protein Wiz OS = Homo sapiens GN = WIZ PE = 1 SV = 2
8	19398	323.1818	644.3493	2	-0.61	53.67	17.75	26.4 (M.expect:0.0,M.score:26.4)	ALBU_HUMAN	Serum albumin OS = Homo sapiens GN = ALB PE = 1 SV = 2
9	21178	328.2289	654.4428	2	0.52	3.88	11.25	26.2 (M.expect:0.0,M.score:26.2)	RL29_HUMAN	60S ribosomal protein L29 OS = Homo sapiens GN = RPL29 PE = 1 SV = 2

10	19206	329.7165	657.4174	2	1.62	54.54	12.98	36.5 (M.expect:0.0,M.score:36.5)	CTRO_HUMAN	Citron Rho-interacting kinase OS = Homo sapiens GN = CIT PE = 1 SV = 2
11	18961	330.6954	659.3755	2	1.02	21.19	19.56	38.1 (M.expect:0.0,M.score:38.1)	H31T_HUMAN	Histone H3.1t OS = Homo sapiens GN = HIST3H3 PE = 1 SV = 3
12	18961	330.6954	659.3755	2	1.02	21.19	19.56	38.1 (M.expect:0.0,M.score:38.1)	H31_HUMAN	Histone H3.1 OS = Homo sapiens GN = HIST1H3A PE = 1 SV = 2
13	18965	332.6920	663.3704	2	-1.48	2.96	18.94	26.9 (M.expect:0.0,M.score:26.9)	RL13_HUMAN	60S ribosomal protein L13 OS = Homo sapiens GN = RPL13 PE = 1 SV = 4
14	18764	333.6824	665.3497	2	0.83	46.77	15.48	37.6 (M.expect:0.0,M.score:37.6)	RL37A_HUMAN	60S ribosomal protein L37a OS = Homo sapiens GN = RPL37A PE = 1 SV = 2
15	18589	336.2443	670.4741	2	-0.21	19.28	17.53	36.4 (M.expect:0.0,M.score:36.4)	H12_HUMAN	Histone H1.2 OS = Homo sapiens GN = HIST1H1C PE = 1 SV = 2
16	18589	336.2443	670.4741	2	-0.21	19.28	17.53	36.4 (M.expect:0.0,M.score:36.4)	H15_HUMAN	Histone H1.5 OS = Homo sapiens GN = HIST1H1B PE = 1 SV = 3
17	18812	339.7012	677.3861	2	2.48	3.23	12.58	36.0 (M.expect:0.0,M.score:36.0)	HYEP_HUMAN	Epoxide hydrolase 1 OS = Homo sapiens GN = EPHX1 PE = 1 SV = 1
18	19366	342.6951	683.3755	2	0.19	3.86	13.08	35.0 (M.expect:0.0,M.score:35.0)	RL5_HUMAN	60S ribosomal protein L5 OS = Homo sapiens GN = RPL5 PE = 1 SV = 3
19	19307	344.2057	686.3963	2	0.78	25.62	14.22	34.3 (M.expect:0.0,M.score:34.3)	HS71A_HUMAN	Heat shock 70 kDa protein 1A OS = Homo sapiens GN = HSPA1A PE = 1 SV = 1

20	193 07	344.2 057	686.3 963	2	0.7 8	25.6 2	14. 22	34.3 (M.expect:0.0,M.scor e:34.3)	HSP7C_HU MAN	Heat shock cognate 71 kDa protein OS = Homo sapiens GN = HSPA8 PE = 1 SV = 1
21	193 07	344.2 057	686.3 963	2	0.7 8	25.6 2	14. 22	34.3 (M.expect:0.0,M.scor e:34.3)	GRP78_HU MAN	78 kDa glucose-regulated protein OS = Homo sapiens GN = HSPA5 PE = 1 SV = 2
22	181 95	347.1 889	692.3 606	2	3.8 2	64.6 5	16. 11	39.8 (M.expect:0.0,M.scor e:39.8)	RL8_HUM AN	60S ribosomal protein L8 OS = Homo sapiens GN = RPL8 PE = 1 SV = 2
23	184 58	350.2 290	698.4 439	2	- 0.7 7	14.9 3	19. 29	49.5 (M.expect:0.0,M.scor e:49.5)	RL8_HUM AN	60S ribosomal protein L8 OS = Homo sapiens GN = RPL8 PE = 1 SV = 2
24	190 36	350.7 400	699.4 643	2	1.6 5	12.7 9	12. 83	30.7 (M.expect:0.0,M.scor e:30.7)	RL37A_HU MAN	60S ribosomal protein L37a OS = Homo sapiens GN = RPL37A PE = 1 SV = 2
25	165 20	351.2 008	700.3 868	2	0.4 5	56.3 8	14. 80	35.2 (M.expect:0.0,M.scor e:35.2)	VIME_HU MAN	Vimentin OS = Homo sapiens GN = VIM PE = 1 SV = 4
26	169 58	352.2 035	702.3 912	2	1.7 9	19.2 2	14. 07	28.0 (M.expect:0.0,M.scor e:28.0)	LAMA2_H UMAN	Laminin subunit alpha-2 OS = Homo sapiens GN = LAMA2 PE = 1 SV = 4
27	196 21	354.7 060	707.3 966	2	1.0 5	3.00	11. 55	37.0 (M.expect:0.0,M.scor e:37.0)	RL13_HU MAN	60S ribosomal protein L13 OS = Homo sapiens GN = RPL13 PE = 1 SV = 4
28	170 81	355.7 212	709.4 275	2	0.5 3	38.9 9	20. 54	35.6 (M.expect:0.0,M.scor e:35.6)	TENS1_HU MAN	Tensin-1 OS = Homo sapiens GN = TNS1 PE = 1 SV = 2
29	404 8	714.3 470	713.3 384	1	1.8 2	2.93	32. 85	43.6 (M.expect:0.0,M.scor e:43.6)	H4_HUMA N	Histone H4 OS = Homo sapiens GN = HIST1H4A PE = 1 SV = 2

30	16648	363.7291	725.4436	2	0.18	1.43	21.63	31.8 (M.expect:0.0,M.score:31.8)	A1BG_HUMAN	Alpha-1B-glycoprotein OS = Homo sapiens GN = A1BG PE = 1 SV = 4
31	18597	364.2577	726.5004	2	0.78	25.56	23.76	31.0 (M.expect:0.0,M.score:31.0)	PARK7_HUMAN	Protein deglycase DJ-1 OS = Homo sapiens GN = PARK7 PE = 1 SV = 2
32	17395	365.2350	728.4545	2	1.25	19.89	12.67	41.8 (M.expect:0.0,M.score:41.8)	RL31_HUMAN	60S ribosomal protein L31 OS = Homo sapiens GN = RPL31 PE = 1 SV = 1
33	18030	365.7270	729.4385	2	1.20	2.50	21.10	36.2 (M.expect:0.0,M.score:36.2)	HS90A_HUMAN	Heat shock protein HSP 90-alpha OS = Homo sapiens GN = HSP90AA1 PE = 1 SV = 5
34	18333	371.7327	741.4497	2	1.57	30.29	17.64	44.8 (M.expect:0.0,M.score:44.8)	RL32_HUMAN	60S ribosomal protein L32 OS = Homo sapiens GN = RPL32 PE = 1 SV = 2
35	16557	373.7242	745.4334	2	0.57	18.09	13.39	35.8 (M.expect:0.0,M.score:35.8)	H12_HUMAN	Histone H1.2 OS = Homo sapiens GN = HIST1H1C PE = 1 SV = 2
36	16557	373.7242	745.4334	2	0.57	18.09	13.39	35.8 (M.expect:0.0,M.score:35.8)	H15_HUMAN	Histone H1.5 OS = Homo sapiens GN = HIST1H1B PE = 1 SV = 3
37	17422	374.2288	746.4439	2	-1.29	5.51	21.67	35.0 (M.expect:0.0,M.score:35.0)	CO6A3_HUMAN	Collagen alpha-3(VI) chain OS = Homo sapiens GN = COL6A3 PE = 1 SV = 5
38	18233	375.2194	748.4232	2	1.48	20.80	20.27	25.2 (M.expect:0.0,M.score:25.2)	RL3_HUMAN	60S ribosomal protein L3 OS = Homo sapiens GN = RPL3 PE = 1 SV = 2
39	15919	376.2169	750.4177	2	2.06	12.92	19.61	31.4 (M.expect:0.0,M.score:31.4)	RL10L_HUMAN	60S ribosomal protein L10-like OS = Homo sapiens GN = RPL10L PE = 1 SV = 3

40	143 62	378.7 041	755.3 926	2	1.4 6	28.6 8	13. 91	45.9 (M.expect:0.0,M.score:45.9)	FLNA_HUMAN	Filamin-A OS = Homo sapiens GN = FLNA PE = 1 SV = 4
41	197 13	378.7 405	755.4 654	2	1.5 0	23.4 9	11. 25	29.0 (M.expect:0.0,M.score:29.0)	HNRPC_HUMAN	Heterogeneous nuclear ribonucleoproteins C1/C2 OS = Homo sapiens GN = HNRNPC PE = 1 SV = 4
42	154 98	379.2 220	756.4 283	2	1.6 2	5.99	15. 94	26.3 (M.expect:0.0,M.score:26.3)	RL31_HUMAN	60S ribosomal protein L31 OS = Homo sapiens GN = RPL31 PE = 1 SV = 1
43	177 63	379.7 009	757.3 871	2	0.1 6	55.9 1	12. 78	31.6 (M.expect:0.0,M.score:31.6)	RL21_HUMAN	60S ribosomal protein L21 OS = Homo sapiens GN = RPL21 PE = 1 SV = 2
44	189 64	379.7 497	757.4 850	2	-0.2 6	1.84	29. 16	37.3 (M.expect:0.0,M.score:37.3)	RL13_HUMAN	60S ribosomal protein L13 OS = Homo sapiens GN = RPL13 PE = 1 SV = 4
45	166 27	381.2 336	760.4 517	2	1.2 5	12.9 8	20. 08	36.1 (M.expect:0.0,M.score:36.1)	ANXA2_HUMAN	Annexin A2 OS = Homo sapiens GN = ANXA2 PE = 1 SV = 2
46	180 49	381.7 092	761.4 032	2	0.8 7	59.0 2	13. 13	27.5 (M.expect:0.0,M.score:27.5)	K1C19_HUMAN	Keratin, type I cytoskeletal 19 OS = Homo sapiens GN = KRT19 PE = 1 SV = 4
47	182 01	383.2 162	764.4 181	2	-0.3 4	5.41	15. 99	49.6 (M.expect:0.0,M.score:49.6)	RL21_HUMAN	60S ribosomal protein L21 OS = Homo sapiens GN = RPL21 PE = 1 SV = 2
48	163 60	383.6 960	765.3 770	2	0.7 0	26.7 8	13. 98	55.1 (M.expect:0.0,M.score:55.1)	K2C8_HUMAN	Keratin, type II cytoskeletal 8 OS = Homo sapiens GN = KRT8 PE = 1 SV = 7
49	174 21	386.2 384	770.4 650	2	-3.7 2	7.91	27. 21	28.2 (M.expect:0.0,M.score:28.2)	CAPG_HUMAN	Macrophage-capping protein OS = Homo sapiens GN = CAPG PE = 1 SV = 2

50	18386	386.7376	771.4603	2	0.44	3.12	17.59	54.6 (M.expect:0.0,M.score:54.6)	RS4X_HUMAN	40S ribosomal protein S4, X isoform OS = Homo sapiens GN = RPS4X PE = 1 SV = 2
51	16869	387.6988	773.3820	2	1.20	14.16	14.17	36.6 (M.expect:0.0,M.score:36.6)	FETUA_HUMAN	Alpha-2-HS-glycoprotein OS = Homo sapiens GN = AHSG PE = 1 SV = 1
52	17944	388.7552	775.4956	2	0.20	32.69	15.99	31.9 (M.expect:0.0,M.score:31.9)	RL5_HUMAN	60S ribosomal protein L5 OS = Homo sapiens GN = RPL5 PE = 1 SV = 3
53	18827	390.7401	779.4654	2	0.35	15.89	19.76	32.3 (M.expect:0.0,M.score:32.3)	AN32A_HUMAN	Acidic leucine-rich nuclear phosphoprotein 32 family member A OS = Homo sapiens GN = ANP32A PE = 1 SV = 1
54	14791	391.2198	780.4242	2	0.93	3.10	12.83	41.4 (M.expect:0.0,M.score:41.4)	APOA1_HUMAN	Apolipoprotein A-I OS = Homo sapiens GN = APOA1 PE = 1 SV = 1
55	16196	394.2149	786.4137	2	2.07	3.87	12.41	42.6 (M.expect:0.0,M.score:42.6)	KPYM_HUMAN	Pyruvate kinase PKM OS = Homo sapiens GN = PKM PE = 1 SV = 4
56	17237	394.2323	786.4501	2	-0.09	15.49	10.66	31.1 (M.expect:0.0,M.score:31.1)	RL13A_HUMAN	60S ribosomal protein L13a OS = Homo sapiens GN = RPL13A PE = 1 SV = 2
57	1522	394.7429	787.4705	2	1.05	13.70	18.57	36.9 (M.expect:0.0,M.score:36.9)	H31T_HUMAN	Histone H3.1t OS = Homo sapiens GN = HIST3H3 PE = 1 SV = 3
58	1522	394.7429	787.4705	2	1.05	13.70	18.57	36.9 (M.expect:0.0,M.score:36.9)	H31_HUMAN	Histone H3.1 OS = Homo sapiens GN = HIST1H3A PE = 1 SV = 2
59	19050	396.7277	791.4402	2	0.76	24.09	11.15	40.1 (M.expect:0.0,M.score:40.1)	PA1B3_HUMAN	Platelet-activating factor acetylhydrolase IB subunit gamma OS = Homo sapiens GN = PAFAH1B3 PE = 1 SV = 1

60	18667	396.7593	791.5018	2	2.78	14.84	11.29	35.6 (M.expect:0.0,M.score:35.6)	RL32_HUMAN	60S ribosomal protein L32 OS = Homo sapiens GN = RPL32 PE = 1 SV = 2
61	34	397.2616	792.5082	2	0.59	2.18	12.31	30.9 (M.expect:0.0,M.score:30.9)	H2A1C_HUMAN	Histone H2A type 1-C OS = Homo sapiens GN = HIST1H2AC PE = 1 SV = 3
62	34	397.2616	792.5082	2	0.59	2.18	12.31	30.9 (M.expect:0.0,M.score:30.9)	H2A1H_HUMAN	Histone H2A type 1-H OS = Homo sapiens GN = HIST1H2AH PE = 1 SV = 3
63	34	397.2616	792.5082	2	0.59	2.18	12.31	30.9 (M.expect:0.0,M.score:30.9)	H2A2B_HUMAN	Histone H2A type 2-B OS = Homo sapiens GN = HIST2H2AB PE = 1 SV = 3
64	13405	400.7360	799.4552	2	2.80	5.85	17.39	37.9 (M.expect:0.0,M.score:37.9)	WDR1_HUMAN	WD repeat-containing protein 1 OS = Homo sapiens GN = WDR1 PE = 1 SV = 4
65	16817	401.2400	800.4644	2	1.44	16.68	26.17	25.9 (M.expect:0.0,M.score:25.9)	ENOA_HUMAN	Alpha-enolase OS = Homo sapiens GN = ENO1 PE = 1 SV = 2
66	5038	402.2535	802.4926	2	-0.08	3.81	10.30	30.0 (M.expect:0.0,M.score:30.0)	H2AV_HUMAN	Histone H2A.V OS = Homo sapiens GN = H2AFV PE = 1 SV = 3
67	13105	403.2199	804.4242	2	1.13	14.70	19.76	56.7 (M.expect:0.0,M.score:56.7)	G3P_HUMAN	Glyceraldehyde-3-phosphate dehydrogenase OS = Homo sapiens GN = GAPDH PE = 1 SV = 3
68	17424	403.2318	804.4494	2	-0.47	15.89	23.18	31.1 (M.expect:0.0,M.score:31.1)	CO6A3_HUMAN	Collagen alpha-3(VI) chain OS = Homo sapiens GN = COL6A3 PE = 1 SV = 5
69	18102	403.7299	805.4446	2	0.83	39.21	19.13	41.9 (M.expect:0.0,M.score:41.9)	ENOA_HUMAN	Alpha-enolase OS = Homo sapiens GN = ENO1 PE = 1 SV = 2

70	170 10	404.2 048	806.3 923	2	3.4 4	16.2 3	20. 33	46.6 (M.expect:0.0,M.scor e:46.6)	K1C19_HU MAN	Keratin, type I cytoskeletal 19 OS = Homo sapiens GN = KRT19 PE = 1 SV = 4
71	170 10	404.2 048	806.3 923	2	3.4 4	16.2 3	20. 33	46.6 (M.expect:0.0,M.scor e:46.6)	K1C10_HU MAN	Keratin, type I cytoskeletal 10 OS = Homo sapiens GN = KRT10 PE = 1 SV = 6
72	170 10	404.2 048	806.3 923	2	3.4 4	16.2 3	20. 33	46.6 (M.expect:0.0,M.scor e:46.6)	K1C18_HU MAN	Keratin, type I cytoskeletal 18 OS = Homo sapiens GN = KRT18 PE = 1 SV = 2
73	183 71	406.2 554	810.4 963	2	- 0.0 9	15.7 0	12. 11	32.6 (M.expect:0.0,M.scor e:32.6)	HMGN1_H UMAN	Non-histone chromosomal protein HMG-14 OS = Homo sapiens GN = HMGN1 PE = 1 SV = 3
74	131 75	406.7 352	811.4 552	2	0.6 9	9.99	19. 14	26.7 (M.expect:0.0,M.scor e:26.7)	NUCL_HU MAN	Nucleolin OS = Homo sapiens GN = NCL PE = 1 SV = 3
75	57	408.7 324	815.4 501	2	0.2 3	55.1 5	14. 79	47.0 (M.expect:0.0,M.scor e:47.0)	H2B1N_H UMAN	Histone H2B type 1-N OS = Homo sapiens GN = HIST1H2BN PE = 1 SV = 3
76	57	408.7 324	815.4 501	2	0.2 3	55.1 5	14. 79	47.0 (M.expect:0.0,M.scor e:47.0)	H2B1K_HU MAN	Histone H2B type 1-K OS = Homo sapiens GN = HIST1H2BK PE = 1 SV = 3
77	57	408.7 324	815.4 501	2	0.2 3	55.1 5	14. 79	47.0 (M.expect:0.0,M.scor e:47.0)	H2B1C_HU MAN	Histone H2B type 1-C/E/F/G/I OS = Homo sapiens GN = HIST1H2BC PE = 1 SV = 4
78	57	408.7 324	815.4 501	2	0.2 3	55.1 5	14. 79	47.0 (M.expect:0.0,M.scor e:47.0)	H2B1H_H UMAN	Histone H2B type 1-H OS = Homo sapiens GN = HIST1H2BH PE = 1 SV = 3
79	183 98	408.7 443	815.4 753	2	- 1.4 0	36.4 8	18. 21	27.5 (M.expect:0.0,M.scor e:27.5)	RS11_HUM AN	40S ribosomal protein S11 OS = Homo sapiens GN = RPS11 PE = 1 SV = 3

80	16161	409.7405	817.4658	2	0.93	8.87	11.65	25.8 (M.expect:0.0,M.score:25.8)	IGHG1_HUMAN	Ig gamma-1 chain C region OS = Homo sapiens GN = IGHG1 PE = 1 SV = 1
81	16567	411.2349	820.4555	2	-0.45	31.56	13.40	40.2 (M.expect:0.0,M.score:40.2)	RL8_HUMAN	60S ribosomal protein L8 OS = Homo sapiens GN = RPL8 PE = 1 SV = 2
82	15361	413.7737	825.5324	2	0.55	5.88	15.73	27.2 (M.expect:0.0,M.score:27.2)	RL8_HUMAN	60S ribosomal protein L8 OS = Homo sapiens GN = RPL8 PE = 1 SV = 2
83	10802	414.7505	827.4865	2	0.03	7.23	13.19	47.6 (M.expect:0.0,M.score:47.6)	FINC_HUMAN	Fibronectin OS = Homo sapiens GN = FN1 PE = 1 SV = 4
84	9875	416.2219	830.4287	2	0.74	17.50	12.11	26.4 (M.expect:0.0,M.score:26.4)	APOA1_HUMAN	Apolipoprotein A-I OS = Homo sapiens GN = APOA1 PE = 1 SV = 1
85	17762	416.2330	830.4498	2	2.07	21.01	17.89	30.7 (M.expect:0.0,M.score:30.7)	RS4X_HUMAN	40S ribosomal protein S4, X isoform OS = Homo sapiens GN = RPS4X PE = 1 SV = 2
86	7536	416.2507	830.4862	2	0.74	18.77	22.35	68.9 (M.expect:0.0,M.score:68.9)	H31_HUMAN	Histone H3.1 OS = Homo sapiens GN = HIST1H3A PE = 1 SV = 2
87	7536	416.2507	830.4862	2	0.74	18.77	22.35	68.9 (M.expect:0.0,M.score:68.9)	H31T_HUMAN	Histone H3.1t OS = Homo sapiens GN = HIST3H3 PE = 1 SV = 3
88	16417	416.7483	831.4814	2	0.68	41.84	12.01	27.1 (M.expect:0.0,M.score:27.1)	FLNA_HUMAN	Filamin-A OS = Homo sapiens GN = FLNA PE = 1 SV = 4
89	16877	418.7227	835.4300	2	0.98	11.31	12.41	43.8 (M.expect:0.0,M.score:43.8)	CO1A1_HUMAN	Collagen alpha-1(I) chain OS = Homo sapiens GN = COL1A1 PE = 1 SV = 5

90	9314	419.2147	836.4141	2	0.86	6.14	15.83	54.9 (M.expect:0.0,M.score:54.9)	H10_HUMAN	Histone H1.0 OS = Homo sapiens GN = H1F0 PE = 1 SV = 3
91	14108	419.7304	837.4457	2	0.60	28.97	12.67	25.0 (M.expect:0.0,M.score:25.0)	VINC_HUMAN	Vinculin OS = Homo sapiens GN = VCL PE = 1 SV = 4
92	5955	420.7382	839.4613	2	0.57	16.60	13.98	67.4 (M.expect:0.0,M.score:67.4)	CO1A2_HUMAN	Collagen alpha-2(I) chain OS = Homo sapiens GN = COL1A2 PE = 1 SV = 7
93	17265	423.2586	844.5018	2	0.98	4.94	23.49	32.6 (M.expect:0.0,M.score:32.6)	H12_HUMAN	Histone H1.2 OS = Homo sapiens GN = HIST1H1C PE = 1 SV = 2
94	8265	424.7356	847.4552	2	1.72	39.44	17.14	26.4 (M.expect:0.0,M.score:26.4)	CO6A2_HUMAN	Collagen alpha-2(VI) chain OS = Homo sapiens GN = COL6A2 PE = 1 SV = 4
95	9140	425.7324	849.4497	2	0.50	32.26	25.24	49.2 (M.expect:0.0,M.score:49.2)	K1C19_HUMAN	Keratin, type I cytoskeletal 19 OS = Homo sapiens GN = KRT19 PE = 1 SV = 4
96	2716	425.7668	849.5185	2	0.70	12.38	19.39	36.2 (M.expect:0.0,M.score:36.2)	H2A1C_HUMAN	Histone H2A type 1-C OS = Homo sapiens GN = HIST1H2AC PE = 1 SV = 3
97	2716	425.7668	849.5185	2	0.70	12.38	19.39	36.2 (M.expect:0.0,M.score:36.2)	H2A1H_HUMAN	Histone H2A type 1-H OS = Homo sapiens GN = HIST1H2AH PE = 1 SV = 3
98	2716	425.7668	849.5185	2	0.70	12.38	19.39	36.2 (M.expect:0.0,M.score:36.2)	H2AV_HUMAN	Histone H2A.V OS = Homo sapiens GN = H2AFV PE = 1 SV = 3
99	15451	426.2715	850.5276	2	1.00	6.40	16.86	30.3 (M.expect:0.0,M.score:30.3)	RL7A_HUMAN	60S ribosomal protein L7a OS = Homo sapiens GN = RPL7A PE = 1 SV = 2

100	16712	429.2821	856.5494	2	0.27	19.63	10.92	39.8 (M.expect:0.0,M.score:39.8)	CASP_HUMAN	Protein CASP OS = Homo sapiens GN = CUX1 PE = 1 SV = 2
101	11197	429.7403	857.4607	2	6.22	25.88	16.61	36.8 (M.expect:0.0,M.score:36.8)	CALD1_HUMAN	Caldesmon OS = Homo sapiens GN = CALD1 PE = 1 SV = 3
102	15980	434.7357	867.4563	2	0.56	20.81	14.75	60.2 (M.expect:0.0,M.score:60.2)	CO1A2_HUMAN	Collagen alpha-2(I) chain OS = Homo sapiens GN = COL1A2 PE = 1 SV = 7
103	13240	435.2587	868.5018	2	1.14	59.77	20.74	26.3 (M.expect:0.0,M.score:26.3)	G3P_HUMAN	Glyceraldehyde-3-phosphate dehydrogenase OS = Homo sapiens GN = GAPDH PE = 1 SV = 3
104	4903	435.7198	869.4243	2	0.82	13.46	16.66	54.3 (M.expect:0.0,M.score:54.3)	K2C8_HUMAN	Keratin, type II cytoskeletal 8 OS = Homo sapiens GN = KRT8 PE = 1 SV = 7
105	5902	436.2590	870.5035	2	-0.16	31.23	15.78	31.1 (M.expect:0.0,M.score:31.1)	H31T_HUMAN	Histone H3.1t OS = Homo sapiens GN = HIST3H3 PE = 1 SV = 3
106	5902	436.2590	870.5035	2	-0.16	31.23	15.78	31.1 (M.expect:0.0,M.score:31.1)	H31_HUMAN	Histone H3.1 OS = Homo sapiens GN = HIST1H3A PE = 1 SV = 2
107	11741	436.7095	871.4036	2	0.99	5.54	12.77	57.3 (M.expect:0.0,M.score:57.3)	VIME_HUMAN	Vimentin OS = Homo sapiens GN = VIM PE = 1 SV = 4
108	4956	438.2585	874.5025	2	0.00	24.59	14.59	27.9 (M.expect:0.0,M.score:27.9)	ALBU_HUMAN	Serum albumin OS = Homo sapiens GN = ALB PE = 1 SV = 2
109	12824	438.7329	875.4501	2	1.24	29.27	20.95	47.8 (M.expect:0.0,M.score:47.8)	CATB_HUMAN	Cathepsin B OS = Homo sapiens GN = CTSB PE = 1 SV = 3

110	9750	439.7367	877.4579	2	1.16	16.67	16.40	28.0 (M.expect:0.0,M.score:28.0)	RL26_HUMAN	60S ribosomal protein L26 OS = Homo sapiens GN = RPL26 PE = 1 SV = 1
111	13703	440.7246	879.4338	2	0.85	42.33	17.43	41.2 (M.expect:0.0,M.score:41.2)	ALBU_HUMAN	Serum albumin OS = Homo sapiens GN = ALB PE = 1 SV = 2
112	11516	441.2509	880.4879	2	-0.67	31.29	14.75	26.6 (M.expect:0.0,M.score:26.6)	RL15_HUMAN	60S ribosomal protein L15 OS = Homo sapiens GN = RPL15 PE = 1 SV = 2
113	12876	442.7817	883.5491	2	-0.29	6.79	13.55	29.6 (M.expect:0.0,M.score:29.6)	NUCL_HUMAN	Nucleolin OS = Homo sapiens GN = NCL PE = 1 SV = 3
114	14985	449.7433	897.4709	2	1.28	59.29	21.62	35.9 (M.expect:0.0,M.score:35.9)	RL15_HUMAN	60S ribosomal protein L15 OS = Homo sapiens GN = RPL15 PE = 1 SV = 2
115	2320	451.2539	900.4930	2	0.25	7.39	10.40	42.5 (M.expect:0.0,M.score:42.5)	H2B1K_HUMAN	Histone H2B type 1-K OS = Homo sapiens GN = HIST1H2BK PE = 1 SV = 3
116	2320	451.2539	900.4930	2	0.25	7.39	10.40	42.5 (M.expect:0.0,M.score:42.5)	H2B1N_HUMAN	Histone H2B type 1-N OS = Homo sapiens GN = HIST1H2BN PE = 1 SV = 3
117	2320	451.2539	900.4930	2	0.25	7.39	10.40	42.5 (M.expect:0.0,M.score:42.5)	H2B1C_HUMAN	Histone H2B type 1-C/E/F/G/I OS = Homo sapiens GN = HIST1H2BC PE = 1 SV = 4
118	2320	451.2539	900.4930	2	0.25	7.39	10.40	42.5 (M.expect:0.0,M.score:42.5)	H2B1H_HUMAN	Histone H2B type 1-H OS = Homo sapiens GN = HIST1H2BH PE = 1 SV = 3
119	6241	451.7681	901.5233	2	-1.79	6.19	18.63	26.0 (M.expect:0.0,M.score:26.0)	RL7A_HUMAN	60S ribosomal protein L7a OS = Homo sapiens GN = RPL7A PE = 1 SV = 2

120	15813	452.2487	902.4821	2	0.73	6.96	11.70	34.2 (M.expect:0.0,M.score:34.2)	ANXA5_HUMAN	Annexin A5 OS = Homo sapiens GN = ANXA5 PE = 1 SV = 2
121	9865	452.7355	903.4549	2	1.59	7.55	15.94	40.0 (M.expect:0.0,M.score:40.0)	ENOA_HUMAN	Alpha-enolase OS = Homo sapiens GN = ENO1 PE = 1 SV = 2
122	14506	454.2142	906.4117	2	2.40	50.46	16.76	29.0 (M.expect:0.0,M.score:29.0)	S10A6_HUMAN	Protein S100-A6 OS = Homo sapiens GN = S100A6 PE = 1 SV = 1
123	18522	454.2555	906.4963	2	0.01	5.79	33.01	31.6 (M.expect:0.0,M.score:31.6)	CH10_HUMAN	10 kDa heat shock protein, mitochondrial OS = Homo sapiens GN = HSPE1 PE = 1 SV = 2
124	12299	456.2153	910.4145	2	1.83	13.33	12.83	29.4 (M.expect:0.0,M.score:29.4)	K2C8_HUMAN	Keratin, type II cytoskeletal 8 OS = Homo sapiens GN = KRT8 PE = 1 SV = 7
125	14555	456.2302	910.4443	2	1.76	14.29	11.39	40.5 (M.expect:0.0,M.score:40.5)	UBA1_HUMAN	Ubiquitin-like modifier-activating enzyme 1 OS = Homo sapiens GN = UBA1 PE = 1 SV = 3
126	9054	456.7688	911.5229	2	0.20	10.11	15.88	36.1 (M.expect:0.0,M.score:36.1)	RL29_HUMAN	60S ribosomal protein L29 OS = Homo sapiens GN = RPL29 PE = 1 SV = 2
127	7042	456.7797	911.5440	2	0.95	4.77	23.39	38.4 (M.expect:0.0,M.score:38.4)	RSSA_HUMAN	40S ribosomal protein SA OS = Homo sapiens GN = RPSA PE = 1 SV = 4
128	15611	457.2709	912.5280	2	-0.84	14.34	19.29	35.4 (M.expect:0.0,M.score:35.4)	RRBP1_HUMAN	Ribosome-binding protein 1 OS = Homo sapiens GN = RRBP1 PE = 1 SV = 4
129	8891	457.7330	913.4505	2	1.05	22.84	14.54	29.3 (M.expect:0.0,M.score:29.3)	VIME_HUMAN	Vimentin OS = Homo sapiens GN = VIM PE = 1 SV = 4

130	9457	457.7878	913.5597	2	1.46	9.81	20.84	51.0 (M.expect:0.0,M.score:51.0)	EF1A1_HUMAN	Elongation factor 1-alpha 1 OS = Homo sapiens GN = EEF1A1 PE = 1 SV = 1
131	8529	458.2488	914.4821	2	0.96	14.74	16.86	36.9 (M.expect:0.0,M.score:36.9)	S10A6_HUMAN	Protein S100-A6 OS = Homo sapiens GN = S100A6 PE = 1 SV = 1
132	12115	458.2739	914.5338	2	-0.65	15.79	26.78	47.6 (M.expect:0.0,M.score:47.6)	H2AY_HUMAN	Core histone macro-H2A.1 OS = Homo sapiens GN = H2AFY PE = 1 SV = 4
133	12989	460.2233	918.4308	2	1.34	23.12	11.30	51.4 (M.expect:0.0,M.score:51.4)	LMNA_HUMAN	Prelamin-A/C OS = Homo sapiens GN = LMNA PE = 1 SV = 1
134	13564	462.2699	922.5236	2	1.79	8.53	16.66	27.2 (M.expect:0.0,M.score:27.2)	HPT_HUMAN	Haptoglobin OS = Homo sapiens GN = HP PE = 1 SV = 1
135	14628	466.2768	930.5386	2	0.57	9.31	20.68	33.9 (M.expect:0.0,M.score:33.9)	IGHA1_HUMAN	Ig alpha-1 chain C region OS = Homo sapiens GN = IGHA1 PE = 1 SV = 2
136	13914	470.3214	938.6277	2	0.66	12.36	11.23	35.8 (M.expect:0.0,M.score:35.8)	RL24_HUMAN	60S ribosomal protein L24 OS = Homo sapiens GN = RPL24 PE = 1 SV = 1
137	13929	470.7457	939.4774	2	-0.58	10.63	15.99	31.5 (M.expect:0.0,M.score:31.5)	ALDOA_HUMAN	Fructose-bisphosphate aldolase A OS = Homo sapiens GN = ALDOA PE = 1 SV = 2
138	8730	471.2564	940.4978	2	0.37	37.89	14.33	32.9 (M.expect:0.0,M.score:32.9)	HSPB1_HUMAN	Heat shock protein beta-1 OS = Homo sapiens GN = HSPB1 PE = 1 SV = 2
139	14309	471.2802	940.5454	2	0.42	7.89	18.47	39.9 (M.expect:0.0,M.score:39.9)	RL8_HUMAN	60S ribosomal protein L8 OS = Homo sapiens GN = RPL8 PE = 1 SV = 2

140	11721	472.2901	942.5651	2	0.49	16.25	19.86	53.6 (M.expect:0.0,M.score:53.6)	HNRPC_HUMAN	Heterogeneous nuclear ribonucleoproteins C1/C2 OS = Homo sapiens GN = HNRNPC PE = 1 SV = 4
141	8356	472.3008	942.5862	2	0.94	18.82	11.80	27.7 (M.expect:0.0,M.score:27.7)	RL8_HUMAN	60S ribosomal protein L8 OS = Homo sapiens GN = RPL8 PE = 1 SV = 2
142	18630	472.7698	943.5240	2	1.14	18.81	50.88	64.5 (M.expect:0.0,M.score:64.5)	H2A1H_HUMAN	Histone H2A type 1-H OS = Homo sapiens GN = HIST1H2AH PE = 1 SV = 3
143	18630	472.7698	943.5240	2	1.14	18.81	50.88	64.5 (M.expect:0.0,M.score:64.5)	H2A1C_HUMAN	Histone H2A type 1-C OS = Homo sapiens GN = HIST1H2AC PE = 1 SV = 3
144	18630	472.7698	943.5240	2	1.14	18.81	50.88	64.5 (M.expect:0.0,M.score:64.5)	H2A2B_HUMAN	Histone H2A type 2-B OS = Homo sapiens GN = HIST2H2AB PE = 1 SV = 3
145	18630	472.7698	943.5240	2	1.14	18.81	50.88	64.5 (M.expect:0.0,M.score:64.5)	H2AV_HUMAN	Histone H2A.V OS = Homo sapiens GN = H2AFV PE = 1 SV = 3
146	19436	317.5240	949.5498	3	0.33	34.33	17.24	44.0 (M.expect:0.0,M.score:44.0)	RL10L_HUMAN	60S ribosomal protein L10-like OS = Homo sapiens GN = RPL10L PE = 1 SV = 3
147	8125	476.7585	951.5025	2	-0.02	3.22	14.02	51.7 (M.expect:0.0,M.score:51.7)	HBB_HUMAN	Hemoglobin subunit beta OS = Homo sapiens GN = HBB PE = 1 SV = 2
148	16890	477.3053	952.5957	2	0.43	6.09	44.14	49.0 (M.expect:0.0,M.score:49.0)	H2B1H_HUMAN	Histone H2B type 1-H OS = Homo sapiens GN = HIST1H2BH PE = 1 SV = 3
149	16890	477.3053	952.5957	2	0.43	6.09	44.14	49.0 (M.expect:0.0,M.score:49.0)	H2B1C_HUMAN	Histone H2B type 1-C/E/F/G/I OS = Homo sapiens GN = HIST1H2BC PE = 1 SV = 4

150	16890	477.3053	952.5957	2	0.43	6.09	44.14	49.0 (M.expect:0.0,M.score:49.0)	H2B1N_HUMAN	Histone H2B type 1-N OS = Homo sapiens GN = HIST1H2BN PE = 1 SV = 3
151	16890	477.3053	952.5957	2	0.43	6.09	44.14	49.0 (M.expect:0.0,M.score:49.0)	H2B1K_HUMAN	Histone H2B type 1-K OS = Homo sapiens GN = HIST1H2BK PE = 1 SV = 3
152	11877	478.7802	955.5451	2	0.76	14.32	16.35	68.6 (M.expect:0.0,M.score:68.6)	RL4_HUMAN	60S ribosomal protein L4 OS = Homo sapiens GN = RPL4 PE = 1 SV = 5
153	9414	479.2726	956.5291	2	1.60	38.87	23.70	43.3 (M.expect:0.0,M.score:43.3)	RL13_HUMAN	60S ribosomal protein L13 OS = Homo sapiens GN = RPL13 PE = 1 SV = 4
154	8460	480.7596	959.5036	2	1.12	14.66	16.76	47.5 (M.expect:0.0,M.score:47.5)	CH60_HUMAN	60 kDa heat shock protein, mitochondrial OS = Homo sapiens GN = HSPD1 PE = 1 SV = 2
155	10190	480.7830	959.5553	2	-3.96	7.98	24.93	34.9 (M.expect:0.0,M.score:34.9)	ALBU_HUMAN	Serum albumin OS = Homo sapiens GN = ALB PE = 1 SV = 2
156	10394	484.7746	967.5338	2	0.81	7.27	11.75	55.6 (M.expect:0.0,M.score:55.6)	H2B1K_HUMAN	Histone H2B type 1-K OS = Homo sapiens GN = HIST1H2BK PE = 1 SV = 3
157	7763	486.7598	971.5036	2	1.54	15.29	13.96	50.3 (M.expect:0.0,M.score:50.3)	LMNA_HUMAN	Prelamin-A/C OS = Homo sapiens GN = LMNA PE = 1 SV = 1
158	9255	486.7780	971.5400	2	1.57	15.61	15.99	36.2 (M.expect:0.0,M.score:36.2)	K2C7_HUMAN	Keratin, type II cytoskeletal 7 OS = Homo sapiens GN = KRT7 PE = 1 SV = 5
159	10410	486.7900	971.5651	2	0.33	12.23	27.51	30.0 (M.expect:0.0,M.score:30.0)	RS25_HUMAN	40S ribosomal protein S25 OS = Homo sapiens GN = RPS25 PE = 1 SV = 1

160	2441	487.3049	972.5968	2	-1.66	6.95	19.18	72.9 (M.expect:0.0,M.score:72.9)	H12_HUMAN	Histone H1.2 OS = Homo sapiens GN = HIST1H1C PE = 1 SV = 2
161	15244	487.3130	972.6120	2	-0.63	16.64	24.62	31.0 (M.expect:0.0,M.score:31.0)	RL13_HUMAN	60S ribosomal protein L13 OS = Homo sapiens GN = RPL13 PE = 1 SV = 4
162	6815	487.7444	973.4716	2	2.65	21.80	16.82	34.9 (M.expect:0.0,M.score:34.9)	LMNA_HUMAN	Prelamin-A/C OS = Homo sapiens GN = LMNA PE = 1 SV = 1
163	6815	487.7444	973.4716	2	2.65	21.80	16.82	34.9 (M.expect:0.0,M.score:34.9)	LMNB2_HUMAN	Lamin-B2 OS = Homo sapiens GN = LMNB2 PE = 1 SV = 4
164	8768	488.7283	975.4410	2	1.06	15.15	17.12	71.3 (M.expect:0.0,M.score:71.3)	ACTA_HUMAN	Actin, aortic smooth muscle OS = Homo sapiens GN = ACTA2 PE = 1 SV = 1
165	8768	488.7283	975.4410	2	1.06	15.15	17.12	71.3 (M.expect:0.0,M.score:71.3)	ACTB_HUMAN	Actin, cytoplasmic 1 OS = Homo sapiens GN = ACTB PE = 1 SV = 1
166	8204	490.7262	979.4359	2	1.94	30.72	18.82	49.7 (M.expect:0.0,M.score:49.7)	FIBB_HUMAN	Fibrinogen beta chain OS = Homo sapiens GN = FGB PE = 1 SV = 2
167	18584	327.5150	979.5240	3	-0.70	10.25	15.80	38.4 (M.expect:0.0,M.score:38.4)	PRDX1_HUMAN	Peroxiredoxin-1 OS = Homo sapiens GN = PRDX1 PE = 1 SV = 1
168	15575	492.7724	983.5288	2	1.41	32.27	11.30	66.1 (M.expect:0.0,M.score:66.1)	H2B1C_HUMAN	Histone H2B type 1-C/E/F/G/I OS = Homo sapiens GN = HIST1H2BC PE = 1 SV = 4
169	15575	492.7724	983.5288	2	1.41	32.27	11.30	66.1 (M.expect:0.0,M.score:66.1)	H2B1N_HUMAN	Histone H2B type 1-N OS = Homo sapiens GN = HIST1H2BN PE = 1 SV = 3

170	15575	492.7724	983.5288	2	1.41	32.27	11.30	66.1 (M.expect:0.0,M.score:66.1)	H2B1H_HUMAN	Histone H2B type 1-H OS = Homo sapiens GN = HIST1H2BH PE = 1 SV = 3
171	13353	493.7621	985.5080	2	1.60	19.66	20.22	31.4 (M.expect:0.0,M.score:31.4)	GRP78_HUMAN	78 kDa glucose-regulated protein OS = Homo sapiens GN = HSPA5 PE = 1 SV = 2
172	15322	495.7839	989.5546	2	-1.37	27.61	26.11	38.6 (M.expect:0.0,M.score:38.6)	CAYP1_HUMAN	Calcyphosin OS = Homo sapiens GN = CAPS PE = 1 SV = 1
173	14843	497.7729	993.5317	2	-0.55	14.39	25.75	51.8 (M.expect:0.0,M.score:51.8)	RL35A_HUMAN	60S ribosomal protein L35a OS = Homo sapiens GN = RPL35A PE = 1 SV = 2
174	8407	500.2592	998.5033	2	0.49	5.14	22.03	34.9 (M.expect:0.0,M.score:34.9)	CO6A3_HUMAN	Collagen alpha-3(VI) chain OS = Homo sapiens GN = COL6A3 PE = 1 SV = 5
175	12106	500.8056	999.5964	2	0.14	21.12	26.32	76.7 (M.expect:0.0,M.score:76.7)	ALBU_HUMAN	Serum albumin OS = Homo sapiens GN = ALB PE = 1 SV = 2
176	6474	1003.5673	1002.5597	1	0.29	5.25	29.62	37.8 (M.expect:0.0,M.score:37.8)	CAP1_HUMAN	Adenylyl cyclase-associated protein 1 OS = Homo sapiens GN = CAP1 PE = 1 SV = 5
177	18456	336.8619	1007.5625	3	1.31	27.34	12.47	37.1 (M.expect:0.0,M.score:37.1)	APOA1_HUMAN	Apolipoprotein A-I OS = Homo sapiens GN = APOA1 PE = 1 SV = 1
178	12937	507.2262	1012.4363	2	1.63	30.08	22.30	25.7 (M.expect:0.0,M.score:25.7)	ROA2_HUMAN	Heterogeneous nuclear ribonucleoproteins A2/B1 OS = Homo sapiens GN = HNRNPA2B1 PE = 1 SV = 2
179	8401	507.3034	1012.5917	2	0.54	3.79	32.65	45.7 (M.expect:0.0,M.score:45.7)	ALBU_HUMAN	Serum albumin OS = Homo sapiens GN = ALB PE = 1 SV = 2

180	8483	507.8190	1013.6233	2	0.12	10.76	21.72	72.5 (M.expect:0.0,M.score:72.5)	H15_HUMAN	Histone H1.5 OS = Homo sapiens GN = HIST1H1B PE = 1 SV = 3
181	15847	508.2919	1014.5710	2	-1.78	20.00	25.70	25.1 (M.expect:0.0,M.score:25.1)	TBA1A_HUMAN	Tubulin alpha-1A chain OS = Homo sapiens GN = TUBA1A PE = 1 SV = 1
182	11513	509.7620	1017.5091	2	0.43	34.67	18.36	51.6 (M.expect:0.0,M.score:51.6)	RL15_HUMAN	60S ribosomal protein L15 OS = Homo sapiens GN = RPL15 PE = 1 SV = 2
183	18430	340.2035	1017.5872	3	1.36	2.82	18.11	28.1 (M.expect:0.0,M.score:28.1)	RL10L_HUMAN	60S ribosomal protein L10-like OS = Homo sapiens GN = RPL10L PE = 1 SV = 3
184	18539	340.5313	1018.5712	3	0.88	4.00	21.25	56.0 (M.expect:0.0,M.score:56.0)	ALBU_HUMAN	Serum albumin OS = Homo sapiens GN = ALB PE = 1 SV = 2
185	14239	512.2590	1022.5033	2	0.23	10.64	14.49	29.8 (M.expect:0.0,M.score:29.8)	VIME_HUMAN	Vimentin OS = Homo sapiens GN = VIM PE = 1 SV = 4
186	15068	513.3032	1024.5917	2	0.21	34.73	21.36	49.8 (M.expect:0.0,M.score:49.8)	RS2_HUMAN	40S ribosomal protein S2 OS = Homo sapiens GN = RPS2 PE = 1 SV = 2
187	12144	513.3083	1024.6029	2	-0.82	5.97	23.70	55.3 (M.expect:0.0,M.score:55.3)	EF1A1_HUMAN	Elongation factor 1-alpha 1 OS = Homo sapiens GN = EEF1A1 PE = 1 SV = 1
188	514	514.7597	1027.5047	2	0.25	4.61	11.60	59.6 (M.expect:0.0,M.score:59.6)	VIME_HUMAN	Vimentin OS = Homo sapiens GN = VIM PE = 1 SV = 4
189	16156	514.7909	1027.5662	2	0.91	2.87	26.22	47.6 (M.expect:0.0,M.score:47.6)	LMNA_HUMAN	Prelamin-A/C OS = Homo sapiens GN = LMNA PE = 1 SV = 1

190	13920	516.7701	1031.5247	2	0.94	26.02	11.34	44.1 (M.expect:0.0,M.score:44.1)	TYB10_HUMAN	Thymosin beta-10 OS = Homo sapiens GN = TMSB10 PE = 1 SV = 2
191	414	516.8011	1031.5876	2	0.09	4.38	17.38	44.1 (M.expect:0.0,M.score:44.1)	H31_HUMAN	Histone H3.1 OS = Homo sapiens GN = HIST1H3A PE = 1 SV = 2
192	414	516.8011	1031.5876	2	0.09	4.38	17.38	44.1 (M.expect:0.0,M.score:44.1)	H31T_HUMAN	Histone H3.1t OS = Homo sapiens GN = HIST3H3 PE = 1 SV = 3
193	1086	519.2726	1036.5302	2	0.41	28.25	17.90	39.4 (M.expect:0.0,M.score:39.4)	CO6A3_HUMAN	Collagen alpha-3(VI) chain OS = Homo sapiens GN = COL6A3 PE = 1 SV = 5
194	11209	520.3009	1038.5862	2	0.89	6.85	31.95	27.8 (M.expect:0.0,M.score:27.8)	TBB5_HUMAN	Tubulin beta chain OS = Homo sapiens GN = TUBB PE = 1 SV = 2
195	8207	523.8020	1045.5920	2	-2.54	4.67	24.11	35.5 (M.expect:0.0,M.score:35.5)	S10A4_HUMAN	Protein S100-A4 OS = Homo sapiens GN = S100A4 PE = 1 SV = 1
196	13773	524.2617	1046.5066	2	2.08	4.20	19.44	30.5 (M.expect:0.0,M.score:30.5)	APOA1_HUMAN	Apolipoprotein A-I OS = Homo sapiens GN = APOA1 PE = 1 SV = 1
197	18016	526.7965	1051.5774	2	0.92	8.85	10.56	25.3 (M.expect:0.0,M.score:25.3)	RL3_HUMAN	60S ribosomal protein L3 OS = Homo sapiens GN = RPL3 PE = 1 SV = 2
198	11959	527.3060	1052.5978	2	-0.30	7.84	12.16	35.8 (M.expect:0.0,M.score:35.8)	RL37A_HUMAN	60S ribosomal protein L37a OS = Homo sapiens GN = RPL37A PE = 1 SV = 2
199	11015	527.7861	1053.5567	2	0.83	14.39	16.92	51.5 (M.expect:0.0,M.score:51.5)	RS14_HUMAN	40S ribosomal protein S14 OS = Homo sapiens GN = RPS14 PE = 1 SV = 3

200	9878	528.2673	1054.5196	2	0.43	12.69	25.14	29.2 (M.expect:0.0,M.score:29.2)	H4_HUMAN	Histone H4 OS = Homo sapiens GN = HIST1H4A PE = 1 SV = 2
201	14117	528.7701	1055.5247	2	0.82	6.95	16.19	33.0 (M.expect:0.0,M.score:33.0)	SH3L3_HUMAN	SH3 domain-binding glutamic acid-rich-like protein 3 OS = Homo sapiens GN = SH3BGRL3 PE = 1 SV = 1
202	11357	533.7622	1065.5091	2	0.67	7.33	19.60	33.4 (M.expect:0.0,M.score:33.4)	PDIA1_HUMAN	Protein disulfide-isomerase OS = Homo sapiens GN = P4HB PE = 1 SV = 3
203	6673	534.3138	1066.6135	2	-0.49	4.84	23.44	49.1 (M.expect:0.0,M.score:49.1)	RS27A_HUMAN	Ubiquitin-40S ribosomal protein S27a OS = Homo sapiens GN = RPS27A PE = 1 SV = 2
204	15453	536.2809	1070.5471	2	0.11	22.33	31.78	34.7 (M.expect:0.0,M.score:34.7)	HBA_HUMAN	Hemoglobin subunit alpha OS = Homo sapiens GN = HBA1 PE = 1 SV = 2
205	12637	537.7748	1073.5353	2	-0.22	7.45	14.80	35.7 (M.expect:0.0,M.score:35.7)	ALBU_HUMAN	Serum albumin OS = Homo sapiens GN = ALB PE = 1 SV = 2
206	11781	539.7979	1077.5819	2	-0.55	15.13	12.42	36.3 (M.expect:0.0,M.score:36.3)	H2B1H_HUMAN	Histone H2B type 1-H OS = Homo sapiens GN = HIST1H2BH PE = 1 SV = 3
207	7219	541.2853	1080.5564	2	-0.30	11.75	13.98	46.2 (M.expect:0.0,M.score:46.2)	K2C8_HUMAN	Keratin, type II cytoskeletal 8 OS = Homo sapiens GN = KRT8 PE = 1 SV = 7
208	18054	361.8896	1082.6448	3	1.98	15.97	17.02	50.1 (M.expect:0.0,M.score:50.1)	RL4_HUMAN	60S ribosomal protein L4 OS = Homo sapiens GN = RPL4 PE = 1 SV = 5
209	15111	543.2956	1084.5764	2	0.17	8.57	27.04	31.3 (M.expect:0.0,M.score:31.3)	ENPL_HUMAN	Endoplasmin OS = Homo sapiens GN = HSP90B1 PE = 1 SV = 1

210	18834	362.5377	1084.5890	3	2.11	9.68	11.30	48.2 (M.expect:0.0,M.score:48.2)	RL3_HUMAN	60S ribosomal protein L3 OS = Homo sapiens GN = RPL3 PE = 1 SV = 2
211	7728	544.2786	1086.5420	2	0.59	3.90	29.31	46.6 (M.expect:0.0,M.score:46.6)	HBA_HUMAN	Hemoglobin subunit alpha OS = Homo sapiens GN = HBA1 PE = 1 SV = 2
212	5870	546.2925	1090.5771	2	-6.19	18.47	26.42	26.2 (M.expect:0.0,M.score:26.2)	EF2_HUMAN	Elongation factor 2 OS = Homo sapiens GN = EEF2 PE = 1 SV = 4
213	14736	546.8063	1091.5975	2	0.53	3.95	11.49	46.5 (M.expect:0.0,M.score:46.5)	H2B1C_HUMAN	Histone H2B type 1-C/E/F/G/I OS = Homo sapiens GN = HIST1H2BC PE = 1 SV = 4
214	14736	546.8063	1091.5975	2	0.53	3.95	11.49	46.5 (M.expect:0.0,M.score:46.5)	H2B1K_HUMAN	Histone H2B type 1-K OS = Homo sapiens GN = HIST1H2BK PE = 1 SV = 3
215	11289	547.2806	1092.5451	2	1.40	37.24	20.43	34.0 (M.expect:0.0,M.score:34.0)	H15_HUMAN	Histone H1.5 OS = Homo sapiens GN = HIST1H1B PE = 1 SV = 3
216	12423	547.8632	1093.7111	2	0.63	10.01	36.18	31.8 (M.expect:0.0,M.score:31.8)	RS16_HUMAN	40S ribosomal protein S16 OS = Homo sapiens GN = RPS16 PE = 1 SV = 2
217	9917	552.3258	1102.6386	2	-1.47	6.04	31.28	51.0 (M.expect:0.0,M.score:51.0)	ARGI1_HUMAN	Arginase-1 OS = Homo sapiens GN = ARG1 PE = 1 SV = 2
218	5685	554.2880	1106.5608	2	0.54	3.05	21.15	48.4 (M.expect:0.0,M.score:48.4)	H12_HUMAN	Histone H1.2 OS = Homo sapiens GN = HIST1H1C PE = 1 SV = 2
219	12602	554.3116	1106.6124	2	-3.45	28.94	30.03	25.3 (M.expect:0.0,M.score:25.3)	RS30_HUMAN	40S ribosomal protein S30 OS = Homo sapiens GN = FAU PE = 1 SV = 1

220	3520	554.8040	1107.5924	2	0.85	7.83	12.63	65.9 (M.expect:0.0,M.score:65.9)	RL23A_HUMAN	60S ribosomal protein L23a OS = Homo sapiens GN = RPL23A PE = 1 SV = 1
221	8188	554.8088	1107.6037	2	-0.53	14.98	13.88	41.2 (M.expect:0.0,M.score:41.2)	FLNA_HUMAN	Filamin-A OS = Homo sapiens GN = FLNA PE = 1 SV = 4
222	11716	555.7756	1109.5353	2	1.16	25.55	21.46	31.0 (M.expect:0.0,M.score:31.0)	FINC_HUMAN	Fibronectin OS = Homo sapiens GN = FN1 PE = 1 SV = 4
223	5520	556.3265	1110.6397	2	-1.06	27.55	26.99	43.2 (M.expect:0.0,M.score:43.2)	RL27A_HUMAN	60S ribosomal protein L27a OS = Homo sapiens GN = RPL27A PE = 1 SV = 2
224	2829	557.2985	1112.5826	2	-0.14	5.31	21.47	25.7 (M.expect:0.0,M.score:25.7)	ARGI1_HUMAN	Arginase-1 OS = Homo sapiens GN = ARG1 PE = 1 SV = 2
225	12380	560.7854	1119.5560	2	0.26	13.16	29.00	47.1 (M.expect:0.0,M.score:47.1)	EZRI_HUMAN	Ezrin OS = Homo sapiens GN = EZR PE = 1 SV = 4
226	17995	374.2457	1119.7128	3	2.16	21.41	23.70	52.2 (M.expect:0.0,M.score:52.2)	ATPA_HUMAN	ATP synthase subunit alpha, mitochondrial OS = Homo sapiens GN = ATP5A1 PE = 1 SV = 1
227	18308	375.5769	1123.7077	3	0.96	9.03	11.65	33.0 (M.expect:0.0,M.score:33.0)	RL4_HUMAN	60S ribosomal protein L4 OS = Homo sapiens GN = RPL4 PE = 1 SV = 5
228	15415	377.1986	1128.5676	3	5.57	10.46	12.63	33.0 (M.expect:0.0,M.score:33.0)	RL5_HUMAN	60S ribosomal protein L5 OS = Homo sapiens GN = RPL5 PE = 1 SV = 3
229	8542	566.7674	1131.5197	2	0.49	16.20	21.57	47.2 (M.expect:0.0,M.score:47.2)	ACTB_HUMAN	Actin, cytoplasmic 1 OS = Homo sapiens GN = ACTB PE = 1 SV = 1

230	11305	567.2879	1132.5612	2	-0.03	16.49	19.55	34.5 (M.expect:0.0,M.score:34.5)	PDIA4_HUMAN	Protein disulfide-isomerase A4 OS = Homo sapiens GN = PDIA4 PE = 1 SV = 2
231	9431	567.7750	1133.5353	2	0.04	5.57	14.64	56.5 (M.expect:0.0,M.score:56.5)	H4_HUMAN	Histone H4 OS = Homo sapiens GN = HIST1H4A PE = 1 SV = 2
232	9915	567.8283	1133.6445	2	-2.11	4.68	33.89	40.7 (M.expect:0.0,M.score:40.7)	ERP29_HUMAN	Endoplasmic reticulum resident protein 29 OS = Homo sapiens GN = ERP29 PE = 1 SV = 4
233	709	570.7804	1139.5459	2	0.33	4.75	19.92	31.1 (M.expect:0.0,M.score:31.1)	FIBA_HUMAN	Fibrinogen alpha chain OS = Homo sapiens GN = FGA PE = 1 SV = 2
234	14892	380.8885	1139.6411	3	2.11	8.63	18.05	25.8 (M.expect:0.0,M.score:25.8)	RL18_HUMAN	60S ribosomal protein L18 OS = Homo sapiens GN = RPL18 PE = 1 SV = 2
235	12919	571.3499	1140.6867	2	-1.32	27.45	27.40	46.3 (M.expect:0.0,M.score:46.3)	ALBU_HUMAN	Serum albumin OS = Homo sapiens GN = ALB PE = 1 SV = 2
236	13059	572.3010	1142.5866	2	0.70	21.37	10.62	50.0 (M.expect:0.0,M.score:50.0)	EZRI_HUMAN	Ezrin OS = Homo sapiens GN = EZR PE = 1 SV = 4
237	10414	382.9212	1145.7397	3	1.86	6.47	12.69	43.0 (M.expect:0.0,M.score:43.0)	RL23_HUMAN	60S ribosomal protein L23 OS = Homo sapiens GN = RPL23 PE = 1 SV = 1
238	3288	575.3113	1148.6077	2	0.30	8.30	25.40	45.6 (M.expect:0.0,M.score:45.6)	ALBU_HUMAN	Serum albumin OS = Homo sapiens GN = ALB PE = 1 SV = 2
239	2195	575.3396	1148.6666	2	-1.76	6.71	19.60	25.2 (M.expect:0.0,M.score:25.2)	HBB_HUMAN	Hemoglobin subunit beta OS = Homo sapiens GN = HBB PE = 1 SV = 2

240	5988	576.8225	1151.6299	2	0.46	4.93	12.26	48.4 (M.expect:0.0,M.score:48.4)	APOA1_HUMAN	Apolipoprotein A-I OS = Homo sapiens GN = APOA1 PE = 1 SV = 1
241	16082	384.9002	1151.6775	3	1.08	4.95	22.62	41.7 (M.expect:0.0,M.score:41.7)	NUCL_HUMAN	Nucleolin OS = Homo sapiens GN = NCL PE = 1 SV = 3
242	8039	578.3046	1154.5932	2	1.26	20.19	29.37	50.7 (M.expect:0.0,M.score:50.7)	RUVB2_HUMAN	RuvB-like 2 OS = Homo sapiens GN = RUVBL2 PE = 1 SV = 3
243	10185	578.3347	1154.6560	2	-1.11	3.48	25.96	42.4 (M.expect:0.0,M.score:42.4)	MYH9_HUMAN	Myosin-9 OS = Homo sapiens GN = MYH9 PE = 1 SV = 4
244	9183	579.3173	1156.6200	2	-0.04	6.90	14.38	46.4 (M.expect:0.0,M.score:46.4)	APOA1_HUMAN	Apolipoprotein A-I OS = Homo sapiens GN = APOA1 PE = 1 SV = 1
245	10604	579.8345	1157.6557	2	-1.06	29.57	29.98	39.6 (M.expect:0.0,M.score:39.6)	RL6_HUMAN	60S ribosomal protein L6 OS = Homo sapiens GN = RPL6 PE = 1 SV = 3
246	8132	582.3133	1162.6135	2	-1.24	17.11	33.47	26.8 (M.expect:0.0,M.score:26.8)	HSPB1_HUMAN	Heat shock protein beta-1 OS = Homo sapiens GN = HSPB1 PE = 1 SV = 2
247	3189	584.8014	1167.5884	2	-0.06	4.80	13.34	56.4 (M.expect:0.0,M.score:56.4)	H2B1C_HUMAN	Histone H2B type 1-C/E/F/G/I OS = Homo sapiens GN = HIST1H2BC PE = 1 SV = 4
248	3189	584.8014	1167.5884	2	-0.06	4.80	13.34	56.4 (M.expect:0.0,M.score:56.4)	H2B1K_HUMAN	Histone H2B type 1-K OS = Homo sapiens GN = HIST1H2BK PE = 1 SV = 3
249	3189	584.8014	1167.5884	2	-0.06	4.80	13.34	56.4 (M.expect:0.0,M.score:56.4)	H2B1H_HUMAN	Histone H2B type 1-H OS = Homo sapiens GN = HIST1H2BH PE = 1 SV = 3

250	3189	584.8014	1167.5884	2	-0.06	4.80	13.34	56.4 (M.expect:0.0,M.score:56.4)	H2B1N_HUMAN	Histone H2B type 1-N OS = Homo sapiens GN = HIST1H2BN PE = 1 SV = 3
251	14828	585.8452	1169.6768	2	-0.92	14.11	28.53	46.2 (M.expect:0.0,M.score:46.2)	RL7_HUMAN	60S ribosomal protein L7 OS = Homo sapiens GN = RPL7 PE = 1 SV = 1
252	5816	586.3365	1170.6608	2	-2.09	10.63	14.59	45.7 (M.expect:0.0,M.score:45.7)	HBA_HUMAN	Hemoglobin subunit alpha OS = Homo sapiens GN = HBA1 PE = 1 SV = 2
253	7671	587.3206	1172.6302	2	-3.06	8.56	16.09	38.8 (M.expect:0.0,M.score:38.8)	RL31_HUMAN	60S ribosomal protein L31 OS = Homo sapiens GN = RPL31 PE = 1 SV = 1
254	4830	589.3103	1176.6060	2	-0.07	22.95	22.30	36.3 (M.expect:0.0,M.score:36.3)	ACTA_HUMAN	Actin, aortic smooth muscle OS = Homo sapiens GN = ACTA2 PE = 1 SV = 1
255	4830	589.3103	1176.6060	2	-0.07	22.95	22.30	36.3 (M.expect:0.0,M.score:36.3)	ACTB_HUMAN	Actin, cytoplasmic 1 OS = Homo sapiens GN = ACTB PE = 1 SV = 1
256	13315	590.3042	1178.5932	2	0.57	3.29	24.32	34.3 (M.expect:0.0,M.score:34.3)	K2C1_HUMAN	Keratin, type II cytoskeletal 1 OS = Homo sapiens GN = KRT1 PE = 1 SV = 6
257	9960	590.8141	1179.6136	2	0.06	19.03	25.40	65.7 (M.expect:0.0,M.score:65.7)	H4_HUMAN	Histone H4 OS = Homo sapiens GN = HIST1H4A PE = 1 SV = 2
258	12805	593.8270	1185.6394	2	0.04	5.01	27.56	46.3 (M.expect:0.0,M.score:46.3)	IGHG1_HUMAN	Ig gamma-1 chain C region OS = Homo sapiens GN = IGHG1 PE = 1 SV = 1
259	5522	594.3717	1186.7285	2	0.22	34.65	17.94	59.6 (M.expect:0.0,M.score:59.6)	H10_HUMAN	Histone H1.0 OS = Homo sapiens GN = H1F0 PE = 1 SV = 3

260	13870	594.8275	1187.6411	2	-0.52	35.39	15.21	38.4 (M.expect:0.0,M.score:38.4)	RL10L_HUMAN	60S ribosomal protein L10-like OS = Homo sapiens GN = RPL10L PE = 1 SV = 3
261	6936	595.8220	1189.6303	2	-0.74	8.34	24.06	49.9 (M.expect:0.0,M.score:49.9)	PDIA4_HUMAN	Protein disulfide-isomerase A4 OS = Homo sapiens GN = PDIA4 PE = 1 SV = 2
262	6123	597.8040	1193.5928	2	0.59	6.50	29.37	31.9 (M.expect:0.0,M.score:31.9)	THRB_HUMAN	Prothrombin OS = Homo sapiens GN = F2 PE = 1 SV = 2
263	10888	599.7651	1197.5150	2	0.49	43.98	15.83	34.3 (M.expect:0.0,M.score:34.3)	ACTA_HUMAN	Actin, aortic smooth muscle OS = Homo sapiens GN = ACTA2 PE = 1 SV = 1
264	10888	599.7651	1197.5150	2	0.49	43.98	15.83	34.3 (M.expect:0.0,M.score:34.3)	ACTB_HUMAN	Actin, cytoplasmic 1 OS = Homo sapiens GN = ACTB PE = 1 SV = 1
265	23	599.8562	1197.6982	2	-0.28	15.94	24.26	106.6 (M.expect:0.0,M.score:106.6)	ACTA_HUMAN	Actin, aortic smooth muscle OS = Homo sapiens GN = ACTA2 PE = 1 SV = 1
266	23	599.8562	1197.6982	2	-0.28	15.94	24.26	106.6 (M.expect:0.0,M.score:106.6)	ACTB_HUMAN	Actin, cytoplasmic 1 OS = Homo sapiens GN = ACTB PE = 1 SV = 1
267	14099	600.3414	1198.6670	2	1.02	5.75	32.24	25.6 (M.expect:0.0,M.score:25.6)	HSP7C_HUMAN	Heat shock cognate 71 kDa protein OS = Homo sapiens GN = HSPA8 PE = 1 SV = 1
268	4957	602.2843	1202.5536	2	0.31	8.72	11.90	31.9 (M.expect:0.0,M.score:31.9)	ACTB_HUMAN	Actin, cytoplasmic 1 OS = Homo sapiens GN = ACTB PE = 1 SV = 1
269	4957	602.2843	1202.5536	2	0.31	8.72	11.90	31.9 (M.expect:0.0,M.score:31.9)	ACTA_HUMAN	Actin, aortic smooth muscle OS = Homo sapiens GN = ACTA2 PE = 1 SV = 1

270	4957	602.2843	1202.5536	2	0.31	8.72	11.90	31.9 (M.expect:0.0,M.score:31.9)	ACTBL_HUMAN	Beta-actin-like protein 2 OS = Homo sapiens GN = ACTBL2 PE = 1 SV = 2
271	11422	602.2941	1202.5667	2	5.82	12.31	18.31	38.7 (M.expect:0.0,M.score:38.7)	LMNA_HUMAN	Prelamin-A/C OS = Homo sapiens GN = LMNA PE = 1 SV = 1
272	18166	604.3489	1206.6833	2	0.02	7.43	10.78	61.3 (M.expect:0.0,M.score:61.3)	RS19_HUMAN	40S ribosomal protein S19 OS = Homo sapiens GN = RPS19 PE = 1 SV = 2
273	12355	606.8446	1211.6762	2	-1.26	20.54	26.27	32.5 (M.expect:0.0,M.score:32.5)	H15_HUMAN	Histone H1.5 OS = Homo sapiens GN = HIST1H1B PE = 1 SV = 3
274	8528	607.2706	1212.5259	2	0.63	2.74	23.49	26.8 (M.expect:0.0,M.score:26.8)	ANXA1_HUMAN	Annexin A1 OS = Homo sapiens GN = ANXA1 PE = 1 SV = 2
275	7037	608.3145	1214.6143	2	0.20	7.42	15.47	82.0 (M.expect:0.0,M.score:82.0)	APOA1_HUMAN	Apolipoprotein A-I OS = Homo sapiens GN = APOA1 PE = 1 SV = 1
276	4411	608.3322	1214.6520	2	-1.81	13.08	16.87	27.7 (M.expect:0.0,M.score:27.7)	RS4X_HUMAN	40S ribosomal protein S4, X isoform OS = Homo sapiens GN = RPS4X PE = 1 SV = 2
277	8086	609.3043	1216.5935	2	0.43	20.20	11.55	28.4 (M.expect:0.0,M.score:28.4)	RL13_HUMAN	60S ribosomal protein L13 OS = Homo sapiens GN = RPL13 PE = 1 SV = 4
278	16353	407.2356	1218.6833	3	1.31	8.45	12.88	49.3 (M.expect:0.0,M.score:49.3)	H2A1C_HUMAN	Histone H2A type 1-C OS = Homo sapiens GN = HIST1H2AC PE = 1 SV = 3
279	16353	407.2356	1218.6833	3	1.31	8.45	12.88	49.3 (M.expect:0.0,M.score:49.3)	H2A1H_HUMAN	Histone H2A type 1-H OS = Homo sapiens GN = HIST1H2AH PE = 1 SV = 3

280	16353	407.2356	1218.6833	3	1.31	8.45	12.88	49.3 (M.expect:0.0,M.score:49.3)	H2A2B_HUMAN	Histone H2A type 2-B OS = Homo sapiens GN = HIST2H2AB PE = 1 SV = 3
281	17992	408.5432	1222.6054	3	1.85	14.23	11.90	64.4 (M.expect:0.0,M.score:64.4)	CO1A2_HUMAN	Collagen alpha-2(I) chain OS = Homo sapiens GN = COL1A2 PE = 1 SV = 7
282	13335	613.2891	1224.5622	2	1.13	8.17	21.98	60.1 (M.expect:0.0,M.score:60.1)	FLNA_HUMAN	Filamin-A OS = Homo sapiens GN = FLNA PE = 1 SV = 4
283	14109	614.3383	1226.6619	2	0.15	4.87	10.61	78.5 (M.expect:0.0,M.score:78.5)	H2B1K_HUMAN	Histone H2B type 1-K OS = Homo sapiens GN = HIST1H2BK PE = 1 SV = 3
284	14109	614.3383	1226.6619	2	0.15	4.87	10.61	78.5 (M.expect:0.0,M.score:78.5)	H2B1N_HUMAN	Histone H2B type 1-N OS = Homo sapiens GN = HIST1H2BN PE = 1 SV = 3
285	14109	614.3383	1226.6619	2	0.15	4.87	10.61	78.5 (M.expect:0.0,M.score:78.5)	H2B1H_HUMAN	Histone H2B type 1-H OS = Homo sapiens GN = HIST1H2BH PE = 1 SV = 3
286	14109	614.3383	1226.6619	2	0.15	4.87	10.61	78.5 (M.expect:0.0,M.score:78.5)	H2B1C_HUMAN	Histone H2B type 1-C/E/F/G/I OS = Homo sapiens GN = HIST1H2BC PE = 1 SV = 4
287	9469	614.8249	1227.6360	2	-0.58	15.59	16.50	41.4 (M.expect:0.0,M.score:41.4)	NONO_HUMAN	Non-POU domain-containing octamer-binding protein OS = Homo sapiens GN = NONO PE = 1 SV = 4
288	8097	617.8058	1233.5949	2	1.65	21.04	17.84	49.7 (M.expect:0.0,M.score:49.7)	TPIS_HUMAN	Triosephosphate isomerase OS = Homo sapiens GN = TPI1 PE = 1 SV = 3
289	11737	617.8294	1233.6452	2	-0.83	9.25	30.24	41.5 (M.expect:0.0,M.score:41.5)	CKAP4_HUMAN	Cytoskeleton-associated protein 4 OS = Homo sapiens GN = CKAP4 PE = 1 SV = 2

290	6307	618.3057	1234.5942	2	2.13	10.18	21.93	27.9 (M.expect:0.0,M.score:27.9)	HS90A_HUMAN	Heat shock protein HSP 90-alpha OS = Homo sapiens GN = HSP90AA1 PE = 1 SV = 5
291	15517	618.3480	1234.6809	2	0.36	5.24	37.45	38.9 (M.expect:0.0,M.score:38.9)	APOA1_HUMAN	Apolipoprotein A-I OS = Homo sapiens GN = APOA1 PE = 1 SV = 1
292	17238	412.9041	1235.6874	3	2.37	5.63	11.14	43.2 (M.expect:0.0,M.score:43.2)	RL23A_HUMAN	60S ribosomal protein L23a OS = Homo sapiens GN = RPL23A PE = 1 SV = 1
293	16521	619.2775	1236.5405	2	-0.06	5.73	11.49	49.4 (M.expect:0.0,M.score:49.4)	ROA2_HUMAN	Heterogeneous nuclear ribonucleoproteins A2/B1 OS = Homo sapiens GN = HNRNPA2B1 PE = 1 SV = 2
294	11417	621.8431	1241.6728	2	-0.97	6.42	23.95	60.3 (M.expect:0.0,M.score:60.3)	RL22_HUMAN	60S ribosomal protein L22 OS = Homo sapiens GN = RPL22 PE = 1 SV = 2
295	6683	621.8549	1241.6980	2	-2.24	18.44	29.93	30.1 (M.expect:0.0,M.score:30.1)	HS90A_HUMAN	Heat shock protein HSP 90-alpha OS = Homo sapiens GN = HSP90AA1 PE = 1 SV = 5
296	6011	625.8592	1249.7030	2	0.58	11.63	20.22	57.3 (M.expect:0.0,M.score:57.3)	H31_HUMAN	Histone H3.1 OS = Homo sapiens GN = HIST1H3A PE = 1 SV = 2
297	6011	625.8592	1249.7030	2	0.58	11.63	20.22	57.3 (M.expect:0.0,M.score:57.3)	H31T_HUMAN	Histone H3.1t OS = Homo sapiens GN = HIST3H3 PE = 1 SV = 3
298	12343	627.8259	1253.6364	2	0.73	18.74	13.70	34.3 (M.expect:0.0,M.score:34.3)	RL34_HUMAN	60S ribosomal protein L34 OS = Homo sapiens GN = RPL34 PE = 1 SV = 3
299	15174	629.3426	1256.6725	2	-1.49	9.30	31.22	59.2 (M.expect:0.0,M.score:59.2)	RRBP1_HUMAN	Ribosome-binding protein 1 OS = Homo sapiens GN = RRBP1 PE = 1 SV = 4

300	5460	629.8616	1257.7081	2	0.42	13.12	29.73	37.0 (M.expect:0.0,M.score:37.0)	MUC5B_HUMAN	Mucin-5B OS = Homo sapiens GN = MUC5B PE = 1 SV = 3
301	2825	630.8797	1259.7449	2	0.02	2.62	18.21	70.6 (M.expect:0.0,M.score:70.6)	H15_HUMAN	Histone H1.5 OS = Homo sapiens GN = HIST1H1B PE = 1 SV = 3
302	2825	630.8797	1259.7449	2	0.02	2.62	18.21	70.6 (M.expect:0.0,M.score:70.6)	H12_HUMAN	Histone H1.2 OS = Homo sapiens GN = HIST1H1C PE = 1 SV = 2
303	5510	634.8076	1267.6004	2	0.13	16.73	13.82	41.5 (M.expect:0.0,M.score:41.5)	LMNB2_HUMAN	Lamin-B2 OS = Homo sapiens GN = LMNB2 PE = 1 SV = 4
304	16614	634.8647	1267.7136	2	1.01	6.80	11.85	33.2 (M.expect:0.0,M.score:33.2)	CRYAB_HUMAN	Alpha-crystallin B chain OS = Homo sapiens GN = CRYAB PE = 1 SV = 2
305	9148	636.8433	1271.6721	2	-0.03	6.81	25.35	65.9 (M.expect:0.0,M.score:65.9)	H2A1H_HUMAN	Histone H2A type 1-H OS = Homo sapiens GN = HIST1H2AH PE = 1 SV = 3
306	10277	636.8565	1271.6973	2	0.84	18.83	37.86	30.3 (M.expect:0.0,M.score:30.3)	K2C7_HUMAN	Keratin, type II cytoskeletal 7 OS = Homo sapiens GN = KRT7 PE = 1 SV = 5
307	15724	425.5711	1273.6891	3	1.84	9.65	21.83	30.2 (M.expect:0.0,M.score:30.2)	H2A2B_HUMAN	Histone H2A type 2-B OS = Homo sapiens GN = HIST2H2AB PE = 1 SV = 3
308	15724	425.5711	1273.6891	3	1.84	9.65	21.83	30.2 (M.expect:0.0,M.score:30.2)	H2A1H_HUMAN	Histone H2A type 1-H OS = Homo sapiens GN = HIST1H2AH PE = 1 SV = 3
309	15724	425.5711	1273.6891	3	1.84	9.65	21.83	30.2 (M.expect:0.0,M.score:30.2)	H2A1C_HUMAN	Histone H2A type 1-C OS = Homo sapiens GN = HIST1H2AC PE = 1 SV = 3

310	15332	639.3422	1276.6697	2	0.06	7.38	26.37	34.4 (M.expect:0.0,M.score:34.4)	RL24_HUMAN	60S ribosomal protein L24 OS = Homo sapiens GN = RPL24 PE = 1 SV = 1
311	12002	642.8425	1283.6721	2	-1.29	33.96	29.47	38.9 (M.expect:0.0,M.score:38.9)	RS6_HUMAN	40S ribosomal protein S6 OS = Homo sapiens GN = RPS6 PE = 1 SV = 1
312	13474	642.8508	1283.6921	2	-3.90	5.67	11.24	27.3 (M.expect:0.0,M.score:27.3)	RL27A_HUMAN	60S ribosomal protein L27a OS = Homo sapiens GN = RPL27A PE = 1 SV = 2
313	10817	643.8404	1285.6667	2	-0.39	4.38	23.80	58.7 (M.expect:0.0,M.score:58.7)	IGHG1_HUMAN	Ig gamma-1 chain C region OS = Homo sapiens GN = IGHG1 PE = 1 SV = 1
314	11294	647.8590	1293.7041	2	-0.55	9.85	13.29	71.2 (M.expect:0.0,M.score:71.2)	H12_HUMAN	Histone H1.2 OS = Homo sapiens GN = HIST1H1C PE = 1 SV = 2
315	11294	647.8590	1293.7041	2	-0.55	9.85	13.29	71.2 (M.expect:0.0,M.score:71.2)	H15_HUMAN	Histone H1.5 OS = Homo sapiens GN = HIST1H1B PE = 1 SV = 3
316	2696	648.3104	1294.6041	2	1.71	4.32	20.79	36.3 (M.expect:0.0,M.score:36.3)	TAGL_HUMAN	Transgelin OS = Homo sapiens GN = TAGLN PE = 1 SV = 4
317	9743	649.3098	1296.6058	2	-0.67	43.08	14.95	40.2 (M.expect:0.0,M.score:40.2)	CO1A1_HUMAN	Collagen alpha-1(I) chain OS = Homo sapiens GN = COL1A1 PE = 1 SV = 5
318	2904	650.8460	1299.6783	2	-0.62	8.09	26.63	38.9 (M.expect:0.0,M.score:38.9)	H2A1C_HUMAN	Histone H2A type 1-C OS = Homo sapiens GN = HIST1H2AC PE = 1 SV = 3
319	11468	651.3281	1300.6412	2	0.43	12.65	20.63	31.1 (M.expect:0.0,M.score:31.1)	APOA1_HUMAN	Apolipoprotein A-I OS = Homo sapiens GN = APOA1 PE = 1 SV = 1

320	12294	651.8302	1301.6463	2	-0.43	4.18	12.42	48.0 (M.expect:0.0,M.score:48.0)	HMGN1_HUMAN	Non-histone chromosomal protein HMG-14 OS = Homo sapiens GN = HMGN1 PE = 1 SV = 3
321	11120	652.3035	1302.5914	2	0.76	8.29	26.11	39.7 (M.expect:0.0,M.score:39.7)	HS71A_HUMAN	Heat shock 70 kDa protein 1A OS = Homo sapiens GN = HSPA1A PE = 1 SV = 1
322	17571	439.2339	1314.6779	3	1.42	11.12	11.09	44.7 (M.expect:0.0,M.score:44.7)	GLU2B_HUMAN	Glucosidase 2 subunit beta OS = Homo sapiens GN = PRKCSH PE = 1 SV = 2
323	5999	662.3129	1322.6103	2	0.70	9.27	23.75	37.5 (M.expect:0.0,M.score:37.5)	VIME_HUMAN	Vimentin OS = Homo sapiens GN = VIM PE = 1 SV = 4
324	4861	662.8366	1323.6644	2	-4.29	25.60	18.47	33.8 (M.expect:0.0,M.score:33.8)	MATR3_HUMAN	Matrin-3 OS = Homo sapiens GN = MATR3 PE = 1 SV = 2
325	10039	663.3802	1324.7463	2	-0.40	8.35	20.84	79.5 (M.expect:0.0,M.score:79.5)	H4_HUMAN	Histone H4 OS = Homo sapiens GN = HIST1H4A PE = 1 SV = 2
326	14070	663.8536	1325.6901	2	1.91	10.06	30.45	46.2 (M.expect:0.0,M.score:46.2)	H4_HUMAN	Histone H4 OS = Homo sapiens GN = HIST1H4A PE = 1 SV = 2
327	8372	666.8545	1331.6933	2	0.95	5.69	23.59	33.3 (M.expect:0.0,M.score:33.3)	ALDOA_HUMAN	Fructose-bisphosphate aldolase A OS = Homo sapiens GN = ALDOA PE = 1 SV = 2
328	7455	668.3491	1334.6830	2	0.48	5.72	22.92	68.6 (M.expect:0.0,M.score:68.6)	H31_HUMAN	Histone H3.1 OS = Homo sapiens GN = HIST1H3A PE = 1 SV = 2
329	7455	668.3491	1334.6830	2	0.48	5.72	22.92	68.6 (M.expect:0.0,M.score:68.6)	H31T_HUMAN	Histone H3.1t OS = Homo sapiens GN = HIST3H3 PE = 1 SV = 3

330	7228	668.3513	1334.6904	2	-1.85	29.76	26.99	46.2 (M.expect:0.0,M.score:46.2)	TBB5_HUMAN	Tubulin beta chain OS = Homo sapiens GN = TUBB PE = 1 SV = 2
331	7118	669.8543	1337.6939	2	0.03	7.39	11.97	39.3 (M.expect:0.0,M.score:39.3)	ROA2_HUMAN	Heterogeneous nuclear ribonucleoproteins A2/B1 OS = Homo sapiens GN = HNRNPA2B1 PE = 1 SV = 2
332	8197	670.8105	1339.6044	2	1.45	2.57	29.36	45.5 (M.expect:0.0,M.score:45.5)	ANXA5_HUMAN	Annexin A5 OS = Homo sapiens GN = ANXA5 PE = 1 SV = 2
333	10411	670.8925	1339.7711	2	-0.53	32.02	21.93	25.1 (M.expect:0.0,M.score:25.1)	H15_HUMAN	Histone H1.5 OS = Homo sapiens GN = HIST1H1B PE = 1 SV = 3
334	15824	671.3408	1340.6684	2	-0.99	6.25	14.02	30.3 (M.expect:0.0,M.score:30.3)	CALD1_HUMAN	Caldesmon OS = Homo sapiens GN = CALD1 PE = 1 SV = 3
335	12566	671.3456	1340.6765	2	0.12	6.57	32.65	55.3 (M.expect:0.0,M.score:55.3)	PDIA3_HUMAN	Protein disulfide-isomerase A3 OS = Homo sapiens GN = PDIA3 PE = 1 SV = 4
336	11661	673.3692	1344.7249	2	-0.75	8.09	26.89	53.5 (M.expect:0.0,M.score:53.5)	RL18_HUMAN	60S ribosomal protein L18 OS = Homo sapiens GN = RPL18 PE = 1 SV = 2
337	1986	677.8152	1353.6161	2	-0.14	4.69	14.54	80.9 (M.expect:0.0,M.score:80.9)	ACTA_HUMAN	Actin, aortic smooth muscle OS = Homo sapiens GN = ACTA2 PE = 1 SV = 1
338	1986	677.8152	1353.6161	2	-0.14	4.69	14.54	80.9 (M.expect:0.0,M.score:80.9)	ACTB_HUMAN	Actin, cytoplasmic 1 OS = Homo sapiens GN = ACTB PE = 1 SV = 1
339	12401	677.8705	1353.7252	2	0.87	4.98	21.93	30.8 (M.expect:0.0,M.score:30.8)	MYL6B_HUMAN	Myosin light chain 6B OS = Homo sapiens GN = MYL6B PE = 1 SV = 1

340	13987	679.3745	1356.7361	2	-1.22	18.24	25.91	54.0 (M.expect:0.0,M.score:54.0)	CO6A3_HUMAN	Collagen alpha-3(VI) chain OS = Homo sapiens GN = COL6A3 PE = 1 SV = 5
341	6403	680.3465	1358.6790	2	-0.37	8.72	17.43	71.4 (M.expect:0.0,M.score:71.4)	LMNA_HUMAN	Prelamin-A/C OS = Homo sapiens GN = LMNA PE = 1 SV = 1
342	12728	680.8517	1359.6882	2	0.45	17.88	35.67	59.3 (M.expect:0.0,M.score:59.3)	RS28_HUMAN	40S ribosomal protein S28 OS = Homo sapiens GN = RPS28 PE = 1 SV = 1
343	13223	685.8505	1369.6878	2	-0.91	6.34	38.31	35.6 (M.expect:0.0,M.score:35.6)	PDIA3_HUMAN	Protein disulfide-isomerase A3 OS = Homo sapiens GN = PDIA3 PE = 1 SV = 4
344	7851	689.3189	1376.6222	2	0.82	10.43	22.19	44.2 (M.expect:0.0,M.score:44.2)	ROA2_HUMAN	Heterogeneous nuclear ribonucleoproteins A2/B1 OS = Homo sapiens GN = HNRNPA2B1 PE = 1 SV = 2
345	12673	689.3520	1376.6896	2	-0.08	15.73	16.40	40.7 (M.expect:0.0,M.score:40.7)	MAP4_HUMAN	Microtubule-associated protein 4 OS = Homo sapiens GN = MAP4 PE = 1 SV = 3
346	10061	689.3780	1376.7412	2	0.11	8.28	18.42	34.4 (M.expect:0.0,M.score:34.4)	RL29_HUMAN	60S ribosomal protein L29 OS = Homo sapiens GN = RPL29 PE = 1 SV = 2
347	5221	691.3285	1380.6409	2	1.07	8.66	22.25	35.0 (M.expect:0.0,M.score:35.0)	K1C10_HUMAN	Keratin, type I cytoskeletal 10 OS = Homo sapiens GN = KRT10 PE = 1 SV = 6
348	10635	695.3779	1388.7412	2	0.00	11.01	32.60	40.9 (M.expect:0.0,M.score:40.9)	CAPG_HUMAN	Macrophage-capping protein OS = Homo sapiens GN = CAPG PE = 1 SV = 2
349	1147	699.8912	1397.7701	2	-1.56	12.02	25.06	26.8 (M.expect:0.0,M.score:26.8)	RL13_HUMAN	60S ribosomal protein L13 OS = Homo sapiens GN = RPL13 PE = 1 SV = 4

350	10840	702.3993	1402.7820	2	1.40	4.36	35.51	30.9 (M.expect:0.0,M.score:30.9)	CO6A3_HUMAN	Collagen alpha-3(VI) chain OS = Homo sapiens GN = COL6A3 PE = 1 SV = 5
351	533	706.3985	1410.7831	2	-0.43	8.21	27.20	41.1 (M.expect:0.0,M.score:41.1)	G3P_HUMAN	Glyceraldehyde-3-phosphate dehydrogenase OS = Homo sapiens GN = GAPDH PE = 1 SV = 3
352	70	714.8602	1427.7045	2	0.93	4.84	23.70	73.7 (M.expect:0.0,M.score:73.7)	VIME_HUMAN	Vimentin OS = Homo sapiens GN = VIM PE = 1 SV = 4
353	12249	717.3447	1432.6735	2	0.86	22.21	30.24	33.7 (M.expect:0.0,M.score:33.7)	CSRP1_HUMAN	Cysteine and glycine-rich protein 1 OS = Homo sapiens GN = CSRP1 PE = 1 SV = 3
354	14098	721.3412	1440.6634	2	3.07	12.57	25.29	63.9 (M.expect:0.0,M.score:63.9)	RS10L_HUMAN	Putative 40S ribosomal protein S10-like OS = Homo sapiens GN = RPS10P5 PE = 5 SV = 1
355	15689	721.8529	1441.6910	2	0.22	8.29	15.52	52.2 (M.expect:0.0,M.score:52.2)	FLNA_HUMAN	Filamin-A OS = Homo sapiens GN = FLNA PE = 1 SV = 4
356	9914	722.8872	1443.7609	2	-0.76	9.22	43.78	29.9 (M.expect:0.0,M.score:29.9)	EF2_HUMAN	Elongation factor 2 OS = Homo sapiens GN = EEF2 PE = 1 SV = 4
357	12471	723.4003	1444.7886	2	-1.79	12.68	30.34	32.0 (M.expect:0.0,M.score:32.0)	EZRI_HUMAN	Ezrin OS = Homo sapiens GN = EZR PE = 1 SV = 4
358	14616	725.4008	1448.7888	2	-1.30	7.22	20.79	47.9 (M.expect:0.0,M.score:47.9)	HBB_HUMAN	Hemoglobin subunit beta OS = Homo sapiens GN = HBB PE = 1 SV = 2
359	10330	729.8939	1457.7726	2	0.42	17.00	30.76	51.9 (M.expect:0.0,M.score:51.9)	ALDOB_HUMAN	Fructose-bisphosphate aldolase B OS = Homo sapiens GN = ALDOB PE = 1 SV = 2

360	9808	730.8580	1459.7015	2	0.00	13.82	12.63	38.6 (M.expect:0.0,M.score:38.6)	PAIRB_HUMAN	Plasminogen activator inhibitor 1 RNA-binding protein OS = Homo sapiens GN = SERBP1 PE = 1 SV = 2
361	4247	730.9030	1459.7922	2	-0.56	12.01	35.62	66.5 (M.expect:0.0,M.score:66.5)	RL18_HUMAN	60S ribosomal protein L18 OS = Homo sapiens GN = RPL18 PE = 1 SV = 2
362	11433	731.8850	1461.7576	2	-1.41	7.58	31.78	31.5 (M.expect:0.0,M.score:31.5)	CO6A3_HUMAN	Collagen alpha-3(VI) chain OS = Homo sapiens GN = COL6A3 PE = 1 SV = 5
363	15456	733.8653	1465.7161	2	-0.04	6.40	13.19	31.0 (M.expect:0.0,M.score:31.0)	TPIS_HUMAN	Triosephosphate isomerase OS = Homo sapiens GN = TPI1 PE = 1 SV = 3
364	9577	733.9053	1465.7963	2	-0.19	8.05	31.53	74.9 (M.expect:0.0,M.score:74.9)	H4_HUMAN	Histone H4 OS = Homo sapiens GN = HIST1H4A PE = 1 SV = 2
365	4029	737.3938	1472.7722	2	0.48	6.02	22.97	60.8 (M.expect:0.0,M.score:60.8)	K2C8_HUMAN	Keratin, type II cytoskeletal 8 OS = Homo sapiens GN = KRT8 PE = 1 SV = 7
366	16659	737.8842	1473.7536	2	0.20	5.46	15.21	53.5 (M.expect:0.0,M.score:53.5)	RL13_HUMAN	60S ribosomal protein L13 OS = Homo sapiens GN = RPL13 PE = 1 SV = 4
367	55	494.6237	1480.8474	3	1.27	3.57	18.47	54.2 (M.expect:0.0,M.score:54.2)	H4_HUMAN	Histone H4 OS = Homo sapiens GN = HIST1H4A PE = 1 SV = 2
368	9486	741.9030	1481.7912	2	0.19	7.71	27.51	50.7 (M.expect:0.0,M.score:50.7)	H4_HUMAN	Histone H4 OS = Homo sapiens GN = HIST1H4A PE = 1 SV = 2
369	13174	744.3549	1486.6940	2	0.90	21.23	26.37	43.1 (M.expect:0.0,M.score:43.1)	HSP7C_HUMAN	Heat shock cognate 71 kDa protein OS = Homo sapiens GN = HSPA8 PE = 1 SV = 1

370	13174	744.3549	1486.6940	2	0.90	21.23	26.37	43.1 (M.expect:0.0,M.score:43.1)	HS71A_HUMAN	Heat shock 70 kDa protein 1A OS = Homo sapiens GN = HSPA1A PE = 1 SV = 1
371	8580	744.8838	1487.7508	2	1.46	21.39	27.87	42.9 (M.expect:0.0,M.score:42.9)	HNRPD_HUMAN	Heterogeneous nuclear ribonucleoprotein D0 OS = Homo sapiens GN = HNRNPD PE = 1 SV = 1
372	13468	751.8654	1501.7161	2	0.03	5.67	12.47	33.0 (M.expect:0.0,M.score:33.0)	LMNA_HUMAN	Prelamin-A/C OS = Homo sapiens GN = LMNA PE = 1 SV = 1
373	711	751.8825	1501.7512	2	-0.49	7.86	31.58	42.3 (M.expect:0.0,M.score:42.3)	IGKC_HUMAN	Ig kappa chain C region OS = Homo sapiens GN = IGKC PE = 1 SV = 1
374	9434	501.9593	1502.8569	3	-0.64	28.59	30.25	61.7 (M.expect:0.0,M.score:61.7)	1433Z_HUMAN	14-3-3 protein zeta/delta OS = Homo sapiens GN = YWHAZ PE = 1 SV = 1
375	9434	501.9593	1502.8569	3	-0.64	28.59	30.25	61.7 (M.expect:0.0,M.score:61.7)	1433B_HUMAN	14-3-3 protein beta/alpha OS = Homo sapiens GN = YWHAB PE = 1 SV = 3
376	1142	754.9250	1507.8358	2	-0.27	3.39	15.26	50.8 (M.expect:0.0,M.score:50.8)	H2B1K_HUMAN	Histone H2B type 1-K OS = Homo sapiens GN = HIST1H2BK PE = 1 SV = 3
377	1142	754.9250	1507.8358	2	-0.27	3.39	15.26	50.8 (M.expect:0.0,M.score:50.8)	H2B1H_HUMAN	Histone H2B type 1-H OS = Homo sapiens GN = HIST1H2BH PE = 1 SV = 3
378	1142	754.9250	1507.8358	2	-0.27	3.39	15.26	50.8 (M.expect:0.0,M.score:50.8)	H2B1C_HUMAN	Histone H2B type 1-C/E/F/G/I OS = Homo sapiens GN = HIST1H2BC PE = 1 SV = 4
379	1142	754.9250	1507.8358	2	-0.27	3.39	15.26	50.8 (M.expect:0.0,M.score:50.8)	H2B1N_HUMAN	Histone H2B type 1-N OS = Homo sapiens GN = HIST1H2BN PE = 1 SV = 3

380	1130	504.2411	1509.6994	3	1.25	28.67	15.20	38.1 (M.expect:0.0,M.score:38.1)	VIME_HUMAN	Vimentin OS = Homo sapiens GN = VIM PE = 1 SV = 4
381	12051	756.4253	1510.8355	2	0.29	14.78	28.07	38.4 (M.expect:0.0,M.score:38.4)	ALBU_HUMAN	Serum albumin OS = Homo sapiens GN = ALB PE = 1 SV = 2
382	8337	757.3966	1512.7784	2	0.19	3.12	31.27	54.6 (M.expect:0.0,M.score:54.6)	HS90A_HUMAN	Heat shock protein HSP 90-alpha OS = Homo sapiens GN = HSP90AA1 PE = 1 SV = 5
383	898	765.3712	1528.7270	2	0.58	3.09	19.91	97.4 (M.expect:0.0,M.score:97.4)	HBA_HUMAN	Hemoglobin subunit alpha OS = Homo sapiens GN = HBA1 PE = 1 SV = 2
384	6653	765.4621	1528.9089	2	0.51	17.46	39.45	57.0 (M.expect:0.0,M.score:57.0)	TYPH_HUMAN	Thymidine phosphorylase OS = Homo sapiens GN = TYMP PE = 1 SV = 2
385	11555	771.9283	1541.8413	2	0.46	5.62	38.82	67.0 (M.expect:0.0,M.score:67.0)	ANXA2_HUMAN	Annexin A2 OS = Homo sapiens GN = ANXA2 PE = 1 SV = 2
386	887	773.9259	1545.8403	2	-1.97	12.40	29.73	48.6 (M.expect:0.0,M.score:48.6)	RL11_HUMAN	60S ribosomal protein L11 OS = Homo sapiens GN = RPL11 PE = 1 SV = 2
387	15948	774.4073	1546.8005	2	-0.23	6.35	12.16	30.8 (M.expect:0.0,M.score:30.8)	RL18_HUMAN	60S ribosomal protein L18 OS = Homo sapiens GN = RPL18 PE = 1 SV = 2
388	10150	774.8608	1547.7063	2	0.39	7.08	19.03	40.5 (M.expect:0.0,M.score:40.5)	1433Z_HUMAN	14-3-3 protein zeta/delta OS = Homo sapiens GN = YWHAZ PE = 1 SV = 1
389	14675	775.9111	1549.8100	2	-1.53	16.45	35.97	28.9 (M.expect:0.0,M.score:28.9)	ANXA1_HUMAN	Annexin A1 OS = Homo sapiens GN = ANXA1 PE = 1 SV = 2

390	4274	777.8790	1553.7434	2	0.03	17.34	23.54	81.0 (M.expect:0.0,M.score:81.0)	K1C19_HUMAN	Keratin, type I cytoskeletal 19 OS = Homo sapiens GN = KRT19 PE = 1 SV = 4
391	9572	781.3449	1560.6733	2	1.28	10.78	33.26	58.7 (M.expect:0.0,M.score:58.7)	NUCL_HUMAN	Nucleolin OS = Homo sapiens GN = NCL PE = 1 SV = 3
392	12073	781.3688	1560.7209	2	1.40	17.79	29.47	39.9 (M.expect:0.0,M.score:39.9)	THRB_HUMAN	Prothrombin OS = Homo sapiens GN = F2 PE = 1 SV = 2
393	1348	781.9000	1561.7849	2	0.36	2.52	21.67	106.4 (M.expect:0.0,M.score:106.4)	CO1A2_HUMAN	Collagen alpha-2(I) chain OS = Homo sapiens GN = COL1A2 PE = 1 SV = 7
394	7762	782.8890	1563.7641	2	-0.48	4.41	17.54	52.9 (M.expect:0.0,M.score:52.9)	H15_HUMAN	Histone H1.5 OS = Homo sapiens GN = HIST1H1B PE = 1 SV = 3
395	6269	783.8795	1565.7434	2	0.69	4.72	24.88	119.6 (M.expect:0.0,M.score:119.6)	LMNA_HUMAN	Prelamin-A/C OS = Homo sapiens GN = LMNA PE = 1 SV = 1
396	10030	783.8933	1565.7726	2	-0.35	26.26	28.69	42.1 (M.expect:0.0,M.score:42.1)	GRP78_HUMAN	78 kDa glucose-regulated protein OS = Homo sapiens GN = HSPA5 PE = 1 SV = 2
397	10380	785.9069	1569.7999	2	-0.38	7.12	25.65	34.4 (M.expect:0.0,M.score:34.4)	FLNA_HUMAN	Filamin-A OS = Homo sapiens GN = FLNA PE = 1 SV = 4
398	9037	785.9517	1569.8879	2	0.55	17.33	40.36	57.3 (M.expect:0.0,M.score:57.3)	VIME_HUMAN	Vimentin OS = Homo sapiens GN = VIM PE = 1 SV = 4
399	10069	786.8416	1571.6700	2	-0.80	10.34	15.78	43.8 (M.expect:0.0,M.score:43.8)	FIBA_HUMAN	Fibrinogen alpha chain OS = Homo sapiens GN = FGA PE = 1 SV = 2

400	11701	788.3919	1574.7689	2	0.27	7.06	14.54	37.8 (M.expect:0.0,M.score:37.8)	H10_HUMAN	Histone H1.0 OS = Homo sapiens GN = H1F0 PE = 1 SV = 3
401	12459	788.3976	1574.7788	2	1.22	12.14	28.07	70.3 (M.expect:0.0,M.score:70.3)	ATPA_HUMAN	ATP synthase subunit alpha, mitochondrial OS = Homo sapiens GN = ATP5A1 PE = 1 SV = 1
402	10618	789.8976	1577.7798	2	0.60	14.37	18.21	75.0 (M.expect:0.0,M.score:75.0)	H12_HUMAN	Histone H1.2 OS = Homo sapiens GN = HIST1H1C PE = 1 SV = 2
403	6137	532.3033	1593.8912	3	-2.01	16.15	28.04	49.3 (M.expect:0.0,M.score:49.3)	H4_HUMAN	Histone H4 OS = Homo sapiens GN = HIST1H4A PE = 1 SV = 2
404	16748	799.8741	1597.7332	2	0.22	10.79	15.26	51.8 (M.expect:0.0,M.score:51.8)	1433B_HUMAN	14-3-3 protein beta/alpha OS = Homo sapiens GN = YWHAB PE = 1 SV = 3
405	12163	535.9429	1604.8046	3	1.32	8.38	23.12	64.1 (M.expect:0.0,M.score:64.1)	LMNA_HUMAN	Prelamin-A/C OS = Homo sapiens GN = LMNA PE = 1 SV = 1
406	8387	804.4071	1606.7991	2	0.33	5.56	35.97	59.5 (M.expect:0.0,M.score:59.5)	FRIL_HUMAN	Ferritin light chain OS = Homo sapiens GN = FTL PE = 1 SV = 2
407	12283	540.9506	1619.8283	3	0.98	12.25	36.12	44.6 (M.expect:0.0,M.score:44.6)	TBB5_HUMAN	Tubulin beta chain OS = Homo sapiens GN = TUBB PE = 1 SV = 2
408	7871	812.9489	1623.8832	2	0.09	4.87	35.51	52.8 (M.expect:0.0,M.score:52.8)	ATPA_HUMAN	ATP synthase subunit alpha, mitochondrial OS = Homo sapiens GN = ATP5A1 PE = 1 SV = 1
409	12584	816.4197	1630.8236	2	0.76	5.69	30.96	21.1 (M.expect:0.1,M.score:21.1)	TBB5_HUMAN	Tubulin beta chain OS = Homo sapiens GN = TUBB PE = 1 SV = 2

410	5007	547.3177	1638.9305	3	0.40	13.64	24.88	39.1 (M.expect:0.0,M.score:39.1)	ALBU_HUMAN	Serum albumin OS = Homo sapiens GN = ALB PE = 1 SV = 2
411	270	548.6130	1642.8162	3	0.48	6.46	15.32	70.1 (M.expect:0.0,M.score:70.1)	HSPB1_HUMAN	Heat shock protein beta-1 OS = Homo sapiens GN = HSPB1 PE = 1 SV = 2
412	8447	830.4055	1658.7940	2	1.49	6.25	34.84	64.5 (M.expect:0.0,M.score:64.5)	SERPH_HUMAN	Serpin H1 OS = Homo sapiens GN = SERPINH1 PE = 1 SV = 2
413	5043	840.8808	1679.7461	2	0.57	5.41	25.04	26.5 (M.expect:0.0,M.score:26.5)	PDIA3_HUMAN	Protein disulfide-isomerase A3 OS = Homo sapiens GN = PDIA3 PE = 1 SV = 4
414	244	844.9159	1687.8166	2	0.36	3.15	19.62	68.5 (M.expect:0.0,M.score:68.5)	RL8_HUMAN	60S ribosomal protein L8 OS = Homo sapiens GN = RPL8 PE = 1 SV = 2
415	11999	847.8543	1693.6928	2	0.70	11.21	15.21	73.7 (M.expect:0.0,M.score:73.7)	ROA1_HUMAN	Heterogeneous nuclear ribonucleoprotein A1 OS = Homo sapiens GN = HNRNPA1 PE = 1 SV = 5
416	9532	848.4374	1694.8588	2	0.84	5.68	20.95	38.8 (M.expect:0.0,M.score:38.8)	YBOX1_HUMAN	Nuclease-sensitive element-binding protein 1 OS = Homo sapiens GN = YBX1 PE = 1 SV = 3
417	4407	851.4564	1700.8985	2	-0.16	14.64	37.25	85.2 (M.expect:0.0,M.score:85.2)	TBA1A_HUMAN	Tubulin alpha-1A chain OS = Homo sapiens GN = TUBA1A PE = 1 SV = 1
418	3449	851.9479	1701.8785	2	1.58	5.37	42.06	85.1 (M.expect:0.0,M.score:85.1)	ANXA1_HUMAN	Annexin A1 OS = Homo sapiens GN = ANXA1 PE = 1 SV = 2
419	14670	852.9549	1703.8941	2	0.69	13.07	42.66	68.3 (M.expect:0.0,M.score:68.3)	ANXA5_HUMAN	Annexin A5 OS = Homo sapiens GN = ANXA5 PE = 1 SV = 2

420	13481	853.4600	1704.9060	2	-0.29	9.30	13.50	28.9 (M.expect:0.0,M.score:28.9)	RL15_HUMAN	60S ribosomal protein L15 OS = Homo sapiens GN = RPL15 PE = 1 SV = 2
421	110	857.9599	1713.9050	2	0.10	3.22	33.89	75.2 (M.expect:0.0,M.score:75.2)	HNRPU_HUMAN	Heterogeneous nuclear ribonucleoprotein U OS = Homo sapiens GN = HNRNPU PE = 1 SV = 6
422	6288	872.4140	1742.8120	2	0.78	4.44	41.89	78.3 (M.expect:0.0,M.score:78.3)	H2B1N_HUMAN	Histone H2B type 1-N OS = Homo sapiens GN = HIST1H2BN PE = 1 SV = 3
423	6288	872.4140	1742.8120	2	0.78	4.44	41.89	78.3 (M.expect:0.0,M.score:78.3)	H2B1K_HUMAN	Histone H2B type 1-K OS = Homo sapiens GN = HIST1H2BK PE = 1 SV = 3
424	6288	872.4140	1742.8120	2	0.78	4.44	41.89	78.3 (M.expect:0.0,M.score:78.3)	H2B1C_HUMAN	Histone H2B type 1-C/E/F/G/I OS = Homo sapiens GN = HIST1H2BC PE = 1 SV = 4
425	6288	872.4140	1742.8120	2	0.78	4.44	41.89	78.3 (M.expect:0.0,M.score:78.3)	H2B1H_HUMAN	Histone H2B type 1-H OS = Homo sapiens GN = HIST1H2BH PE = 1 SV = 3
426	72	876.0253	1750.0353	2	0.39	3.65	33.82	80.4 (M.expect:0.0,M.score:80.4)	H2B1K_HUMAN	Histone H2B type 1-K OS = Homo sapiens GN = HIST1H2BK PE = 1 SV = 3
427	72	876.0253	1750.0353	2	0.39	3.65	33.82	80.4 (M.expect:0.0,M.score:80.4)	H2B1N_HUMAN	Histone H2B type 1-N OS = Homo sapiens GN = HIST1H2BN PE = 1 SV = 3
428	72	876.0253	1750.0353	2	0.39	3.65	33.82	80.4 (M.expect:0.0,M.score:80.4)	H2B1H_HUMAN	Histone H2B type 1-H OS = Homo sapiens GN = HIST1H2BH PE = 1 SV = 3
429	72	876.0253	1750.0353	2	0.39	3.65	33.82	80.4 (M.expect:0.0,M.score:80.4)	H2B1C_HUMAN	Histone H2B type 1-C/E/F/G/I OS = Homo sapiens GN = HIST1H2BC PE = 1 SV = 4

430	7270	876.9341	1751.8551	2	-0.85	8.86	21.67	30.0 (M.expect:0.0,M.score:30.0)	LMNA_HUMAN	Prelamin-A/C OS = Homo sapiens GN = LMNA PE = 1 SV = 1
431	7086	586.3265	1755.9560	3	0.96	4.30	32.55	27.9 (M.expect:0.0,M.score:27.9)	TBA1A_HUMAN	Tubulin alpha-1A chain OS = Homo sapiens GN = TUBA1A PE = 1 SV = 1
432	95	880.4114	1758.8069	2	0.74	5.14	39.96	84.4 (M.expect:0.0,M.score:84.4)	H2B1C_HUMAN	Histone H2B type 1-C/E/F/G/I OS = Homo sapiens GN = HIST1H2BC PE = 1 SV = 4
433	95	880.4114	1758.8069	2	0.74	5.14	39.96	84.4 (M.expect:0.0,M.score:84.4)	H2B1N_HUMAN	Histone H2B type 1-N OS = Homo sapiens GN = HIST1H2BN PE = 1 SV = 3
434	95	880.4114	1758.8069	2	0.74	5.14	39.96	84.4 (M.expect:0.0,M.score:84.4)	H2B1K_HUMAN	Histone H2B type 1-K OS = Homo sapiens GN = HIST1H2BK PE = 1 SV = 3
435	95	880.4114	1758.8069	2	0.74	5.14	39.96	84.4 (M.expect:0.0,M.score:84.4)	H2B1H_HUMAN	Histone H2B type 1-H OS = Homo sapiens GN = HIST1H2BH PE = 1 SV = 3
436	1879	590.6671	1768.9795	3	-0.11	6.52	15.42	33.3 (M.expect:0.0,M.score:33.3)	RL6_HUMAN	60S ribosomal protein L6 OS = Homo sapiens GN = RPL6 PE = 1 SV = 3
437	127	888.4088	1774.8018	2	0.67	6.27	36.79	82.2 (M.expect:0.0,M.score:82.2)	H2B1C_HUMAN	Histone H2B type 1-C/E/F/G/I OS = Homo sapiens GN = HIST1H2BC PE = 1 SV = 4
438	127	888.4088	1774.8018	2	0.67	6.27	36.79	82.2 (M.expect:0.0,M.score:82.2)	H2B1K_HUMAN	Histone H2B type 1-K OS = Homo sapiens GN = HIST1H2BK PE = 1 SV = 3
439	127	888.4088	1774.8018	2	0.67	6.27	36.79	82.2 (M.expect:0.0,M.score:82.2)	H2B1N_HUMAN	Histone H2B type 1-N OS = Homo sapiens GN = HIST1H2BN PE = 1 SV = 3

440	127	888.4088	1774.8018	2	0.67	6.27	36.79	82.2 (M.expect:0.0,M.score:82.2)	H2B1H_HUMAN	Histone H2B type 1-H OS = Homo sapiens GN = HIST1H2BH PE = 1 SV = 3
441	9480	890.9222	1779.8275	2	1.33	11.60	28.07	49.9 (M.expect:0.0,M.score:49.9)	PDIA1_HUMAN	Protein disulfide-isomerase OS = Homo sapiens GN = P4HB PE = 1 SV = 3
442	8027	893.4538	1784.8905	2	1.41	16.05	31.17	37.9 (M.expect:0.0,M.score:37.9)	ENPL_HUMAN	Endoplasmin OS = Homo sapiens GN = HSP90B1 PE = 1 SV = 1
443	8310	894.4663	1786.9200	2	-1.09	4.35	30.60	26.1 (M.expect:0.0,M.score:26.1)	RS27A_HUMAN	Ubiquitin-40S ribosomal protein S27a OS = Homo sapiens GN = RPS27A PE = 1 SV = 2
444	665	895.9496	1789.8846	2	0.05	5.59	33.41	89.4 (M.expect:0.0,M.score:89.4)	ACTB_HUMAN	Actin, cytoplasmic 1 OS = Homo sapiens GN = ACTB PE = 1 SV = 1
445	665	895.9496	1789.8846	2	0.05	5.59	33.41	89.4 (M.expect:0.0,M.score:89.4)	ACTA_HUMAN	Actin, aortic smooth muscle OS = Homo sapiens GN = ACTA2 PE = 1 SV = 1
446	665	895.9496	1789.8846	2	0.05	5.59	33.41	89.4 (M.expect:0.0,M.score:89.4)	ACTBL_HUMAN	Beta-actin-like protein 2 OS = Homo sapiens GN = ACTBL2 PE = 1 SV = 2
447	6733	896.3690	1790.7204	2	1.64	11.98	14.85	34.5 (M.expect:0.0,M.score:34.5)	K1C9_HUMAN	Keratin, type I cytoskeletal 9 OS = Homo sapiens GN = KRT9 PE = 1 SV = 3
448	10102	598.6516	1792.9319	3	0.55	8.26	14.95	29.2 (M.expect:0.0,M.score:29.2)	H2B1C_HUMAN	Histone H2B type 1-C/E/F/G/I OS = Homo sapiens GN = HIST1H2BC PE = 1 SV = 4
449	10102	598.6516	1792.9319	3	0.55	8.26	14.95	29.2 (M.expect:0.0,M.score:29.2)	H2B1N_HUMAN	Histone H2B type 1-N OS = Homo sapiens GN = HIST1H2BN PE = 1 SV = 3

450	10102	598.6516	1792.9319	3	0.55	8.26	14.95	29.2 (M.expect:0.0,M.score:29.2)	H2B1H_HUMAN	Histone H2B type 1-H OS = Homo sapiens GN = HIST1H2BH PE = 1 SV = 3
451	4164	898.4167	1794.8160	2	1.64	5.61	30.08	63.9 (M.expect:0.0,M.score:63.9)	YBOX1_HUMAN	Nuclease-sensitive element-binding protein 1 OS = Homo sapiens GN = YBX1 PE = 1 SV = 3
452	182	900.9915	1799.9669	2	0.81	13.24	40.78	32.7 (M.expect:0.0,M.score:32.7)	RL23A_HUMAN	60S ribosomal protein L23a OS = Homo sapiens GN = RPL23A PE = 1 SV = 1
453	4120	902.9760	1803.9367	2	0.47	11.37	34.69	58.2 (M.expect:0.0,M.score:58.2)	ENOA_HUMAN	Alpha-enolase OS = Homo sapiens GN = ENO1 PE = 1 SV = 2
454	15486	604.3436	1810.0101	3	-0.57	19.92	33.41	48.5 (M.expect:0.0,M.score:48.5)	RL7A_HUMAN	60S ribosomal protein L7a OS = Homo sapiens GN = RPL7A PE = 1 SV = 2
455	12176	606.0035	1814.9890	3	-0.14	6.52	29.57	29.2 (M.expect:0.0,M.score:29.2)	GRP78_HUMAN	78 kDa glucose-regulated protein OS = Homo sapiens GN = HSPA5 PE = 1 SV = 2
456	6996	608.9869	1823.9377	3	0.60	10.08	23.95	36.5 (M.expect:0.0,M.score:36.5)	VIME_HUMAN	Vimentin OS = Homo sapiens GN = VIM PE = 1 SV = 4
457	324	611.9686	1832.8846	3	-0.42	10.05	26.80	50.3 (M.expect:0.0,M.score:50.3)	HBA_HUMAN	Hemoglobin subunit alpha OS = Homo sapiens GN = HBA1 PE = 1 SV = 2
458	6061	918.4789	1834.9425	2	0.44	19.85	32.96	30.0 (M.expect:0.0,M.score:30.0)	IGHA1_HUMAN	Ig alpha-1 chain C region OS = Homo sapiens GN = IGHA1 PE = 1 SV = 2
459	1851	918.9039	1835.7922	2	0.54	8.20	19.70	55.8 (M.expect:0.0,M.score:55.8)	VIME_HUMAN	Vimentin OS = Homo sapiens GN = VIM PE = 1 SV = 4

460	7139	929.0205	1856.0255	2	0.49	11.86	38.36	48.1 (M.expect:0.0,M.score:48.1)	RRBP1_HUMAN	Ribosome-binding protein 1 OS = Homo sapiens GN = RRBP1 PE = 1 SV = 4
461	7983	942.4789	1882.9385	2	2.49	6.83	38.41	47.2 (M.expect:0.0,M.score:47.2)	UBA1_HUMAN	Ubiquitin-like modifier-activating enzyme 1 OS = Homo sapiens GN = UBA1 PE = 1 SV = 3
462	2531	942.9651	1883.9153	2	0.22	9.43	28.28	36.3 (M.expect:0.0,M.score:36.3)	TKT_HUMAN	Transketolase OS = Homo sapiens GN = TKT PE = 1 SV = 3
463	10671	952.9643	1903.9123	2	0.88	2.23	27.56	77.1 (M.expect:0.0,M.score:77.1)	CKAP4_HUMAN	Cytoskeleton-associated protein 4 OS = Homo sapiens GN = CKAP4 PE = 1 SV = 2
464	145	953.5000	1904.9844	2	0.59	5.04	33.01	62.4 (M.expect:0.0,M.score:62.4)	HSPB1_HUMAN	Heat shock protein beta-1 OS = Homo sapiens GN = HSPB1 PE = 1 SV = 2
465	914	955.4643	1908.9105	2	1.84	9.78	34.28	30.3 (M.expect:0.0,M.score:30.3)	FLNA_HUMAN	Filamin-A OS = Homo sapiens GN = FLNA PE = 1 SV = 4
466	14034	955.9001	1909.7827	2	1.51	9.05	19.91	93.9 (M.expect:0.0,M.score:93.9)	ROA3_HUMAN	Heterogeneous nuclear ribonucleoprotein A3 OS = Homo sapiens GN = HNRNPA3 PE = 1 SV = 2
467	8588	957.0029	1911.9902	2	0.60	9.48	31.42	51.7 (M.expect:0.0,M.score:51.7)	FINC_HUMAN	Fibronectin OS = Homo sapiens GN = FN1 PE = 1 SV = 4
468	6691	644.3945	1930.1615	3	0.01	4.21	40.16	76.4 (M.expect:0.0,M.score:76.4)	H2A1H_HUMAN	Histone H2A type 1-H OS = Homo sapiens GN = HIST1H2AH PE = 1 SV = 3
469	6691	644.3945	1930.1615	3	0.01	4.21	40.16	76.4 (M.expect:0.0,M.score:76.4)	H2A1C_HUMAN	Histone H2A type 1-C OS = Homo sapiens GN = HIST1H2AC PE = 1 SV = 3

470	683	966.5083	1931.0000	2	1.00	14.38	32.82	31.2 (M.expect:0.0,M.score:31.2)	CO6A1_HUMAN	Collagen alpha-1(VI) chain OS = Homo sapiens GN = COL6A1 PE = 1 SV = 3
471	1230	967.5102	1933.0044	2	0.76	22.85	42.35	74.9 (M.expect:0.0,M.score:74.9)	TENA_HUMAN	Tenascin OS = Homo sapiens GN = TNC PE = 1 SV = 3
472	6845	651.0066	1949.9959	3	1.08	17.39	24.62	51.5 (M.expect:0.0,M.score:51.5)	FIBB_HUMAN	Fibrinogen beta chain OS = Homo sapiens GN = FGB PE = 1 SV = 2
473	25	652.0268	1953.0571	3	0.66	2.63	27.09	91.4 (M.expect:0.0,M.score:91.4)	ACTB_HUMAN	Actin, cytoplasmic 1 OS = Homo sapiens GN = ACTB PE = 1 SV = 1
474	9893	994.5194	1987.0262	2	-1.01	5.27	37.50	25.5 (M.expect:0.0,M.score:25.5)	ATPB_HUMAN	ATP synthase subunit beta, mitochondrial OS = Homo sapiens GN = ATP5B PE = 1 SV = 3
475	8508	665.3328	1992.9694	3	3.62	8.11	38.17	30.2 (M.expect:0.0,M.score:30.2)	K2C1_HUMAN	Keratin, type II cytoskeletal 1 OS = Homo sapiens GN = KRT1 PE = 1 SV = 6
476	11692	507.5100	2026.0093	4	0.85	27.65	17.02	25.9 (M.expect:0.0,M.score:25.9)	CO1A2_HUMAN	Collagen alpha-2(I) chain OS = Homo sapiens GN = COL1A2 PE = 1 SV = 7
477	1623	1016.5294	2031.0425	2	0.85	4.35	34.44	51.9 (M.expect:0.0,M.score:51.9)	GSTP1_HUMAN	Glutathione S-transferase P OS = Homo sapiens GN = GSTP1 PE = 1 SV = 2
478	9103	688.9995	2063.9760	3	0.35	28.95	29.47	29.6 (M.expect:0.0,M.score:29.6)	ANXA2_HUMAN	Annexin A2 OS = Homo sapiens GN = ANXA2 PE = 1 SV = 2
479	8102	1038.5953	2075.1739	2	1.02	9.87	48.03	32.3 (M.expect:0.0,M.score:32.3)	VINC_HUMAN	Vinculin OS = Homo sapiens GN = VCL PE = 1 SV = 4

480	1921	696.3642	2086.0695	3	0.54	4.15	34.64	37.1 (M.expect:0.0,M.score:37.1)	TBB5_HUMAN	Tubulin beta chain OS = Homo sapiens GN = TUBB PE = 1 SV = 2
481	2449	1044.5490	2087.0840	2	-0.31	6.30	40.26	40.2 (M.expect:0.0,M.score:40.2)	DESM_HUMAN	Desmin OS = Homo sapiens GN = DES PE = 1 SV = 3
482	9297	1049.5402	2097.0630	2	1.36	4.52	47.17	115.9 (M.expect:0.0,M.score:115.9)	BGH3_HUMAN	Transforming growth factor-beta-induced protein ig-h3 OS = Homo sapiens GN = TGFBI PE = 1 SV = 1
483	12194	526.8027	2103.1800	4	0.82	6.18	28.48	30.9 (M.expect:0.0,M.score:30.9)	H2A1H_HUMAN	Histone H2A type 1-H OS = Homo sapiens GN = HIST1H2AH PE = 1 SV = 3
484	2515	1063.5385	2125.0579	2	2.08	7.78	42.37	76.9 (M.expect:0.0,M.score:76.9)	VIME_HUMAN	Vimentin OS = Homo sapiens GN = VIM PE = 1 SV = 4
485	8397	533.8044	2131.1862	4	1.16	5.41	29.10	66.4 (M.expect:0.0,M.score:66.4)	H2A1C_HUMAN	Histone H2A type 1-C OS = Homo sapiens GN = HIST1H2AC PE = 1 SV = 3
486	14120	1068.4899	2134.9614	2	1.79	13.94	19.39	37.5 (M.expect:0.0,M.score:37.5)	IGKC_HUMAN	Ig kappa chain C region OS = Homo sapiens GN = IGKC PE = 1 SV = 1
487	502	1083.0017	2163.9848	2	1.82	4.57	37.87	45.7 (M.expect:0.0,M.score:45.7)	GRP78_HUMAN	78 kDa glucose-regulated protein OS = Homo sapiens GN = HSPA5 PE = 1 SV = 2
488	6974	1096.5911	2191.1637	2	1.82	5.60	46.42	62.6 (M.expect:0.0,M.score:62.6)	CO6A3_HUMAN	Collagen alpha-3(VI) chain OS = Homo sapiens GN = COL6A3 PE = 1 SV = 5
489	3981	1100.5201	2199.0179	2	3.49	11.65	29.47	31.5 (M.expect:0.0,M.score:31.5)	NUCL_HUMAN	Nucleolin OS = Homo sapiens GN = NCL PE = 1 SV = 3

490	12605	1103.4587	2204.8930	2	4.50	21.16	21.57	28.5 (M.expect:0.0,M.score:28.5)	ROA2_HUMAN	Heterogeneous nuclear ribonucleoproteins A2/B1 OS = Homo sapiens GN = HNRNPA2B1 PE = 1 SV = 2
491	16088	1104.0992	2206.1786	2	2.31	4.66	50.46	65.0 (M.expect:0.0,M.score:65.0)	CO6A3_HUMAN	Collagen alpha-3(VI) chain OS = Homo sapiens GN = COL6A3 PE = 1 SV = 5
492	71	1116.0386	2230.0576	2	2.28	4.25	34.76	82.1 (M.expect:0.0,M.score:82.1)	ACTB_HUMAN	Actin, cytoplasmic 1 OS = Homo sapiens GN = ACTB PE = 1 SV = 1
493	241	784.4233	2350.2506	3	-1.01	18.54	29.63	41.4 (M.expect:0.0,M.score:41.4)	IF4H_HUMAN	Eukaryotic translation initiation factor 4H OS = Homo sapiens GN = EIF4H PE = 1 SV = 5
494	2094	784.7307	2351.1717	3	-0.58	11.39	36.94	27.2 (M.expect:0.0,M.score:27.2)	ANXA1_HUMAN	Annexin A1 OS = Homo sapiens GN = ANXA1 PE = 1 SV = 2
495	9402	1192.4822	2382.9446	2	2.18	4.80	18.16	35.2 (M.expect:0.0,M.score:35.2)	K2C1_HUMAN	Keratin, type II cytoskeletal 1 OS = Homo sapiens GN = KRT1 PE = 1 SV = 6
496	12161	810.0643	2427.1675	3	1.46	6.20	46.26	41.2 (M.expect:0.0,M.score:41.2)	H2B1H_HUMAN	Histone H2B type 1-H OS = Homo sapiens GN = HIST1H2BH PE = 1 SV = 3
497	12161	810.0643	2427.1675	3	1.46	6.20	46.26	41.2 (M.expect:0.0,M.score:41.2)	H2B1C_HUMAN	Histone H2B type 1-C/E/F/G/I OS = Homo sapiens GN = HIST1H2BC PE = 1 SV = 4
498	12161	810.0643	2427.1675	3	1.46	6.20	46.26	41.2 (M.expect:0.0,M.score:41.2)	H2B1K_HUMAN	Histone H2B type 1-K OS = Homo sapiens GN = HIST1H2BK PE = 1 SV = 3
499	12161	810.0643	2427.1675	3	1.46	6.20	46.26	41.2 (M.expect:0.0,M.score:41.2)	H2B1N_HUMAN	Histone H2B type 1-N OS = Homo sapiens GN = HIST1H2BN PE = 1 SV = 3

500	16672	815.3952	2443.1624	3	0.57	20.91	44.61	28.1 (M.expect:0.0,M.score:28.1)	H2B1H_HUMAN	Histone H2B type 1-H OS = Homo sapiens GN = HIST1H2BH PE = 1 SV = 3
501	16672	815.3952	2443.1624	3	0.57	20.91	44.61	28.1 (M.expect:0.0,M.score:28.1)	H2B1C_HUMAN	Histone H2B type 1-C/E/F/G/I OS = Homo sapiens GN = HIST1H2BC PE = 1 SV = 4
502	16672	815.3952	2443.1624	3	0.57	20.91	44.61	28.1 (M.expect:0.0,M.score:28.1)	H2B1K_HUMAN	Histone H2B type 1-K OS = Homo sapiens GN = HIST1H2BK PE = 1 SV = 3
503	16672	815.3952	2443.1624	3	0.57	20.91	44.61	28.1 (M.expect:0.0,M.score:28.1)	H2B1N_HUMAN	Histone H2B type 1-N OS = Homo sapiens GN = HIST1H2BN PE = 1 SV = 3
504	310	817.1184	2448.3336	3	-0.08	3.50	36.99	48.2 (M.expect:0.0,M.score:48.2)	K2C7_HUMAN	Keratin, type II cytoskeletal 7 OS = Homo sapiens GN = KRT7 PE = 1 SV = 5
505	8779	821.3839	2461.1205	3	3.78	14.50	13.97	32.7 (M.expect:0.0,M.score:32.7)	CALX_HUMAN	Calnexin OS = Homo sapiens GN = CANX PE = 1 SV = 2
506	11176	1232.0932	2462.1635	2	3.35	7.29	45.52	59.8 (M.expect:0.0,M.score:59.8)	TENA_HUMAN	Tenascin OS = Homo sapiens GN = TNC PE = 1 SV = 3
507	6072	1240.0911	2478.1584	2	3.67	5.64	43.69	74.1 (M.expect:0.0,M.score:74.1)	TENA_HUMAN	Tenascin OS = Homo sapiens GN = TNC PE = 1 SV = 3
508	1874	625.0504	2496.1670	4	2.15	11.25	29.69	25.6 (M.expect:0.0,M.score:25.6)	TBA1A_HUMAN	Tubulin alpha-1A chain OS = Homo sapiens GN = TUBA1A PE = 1 SV = 1
509	6530	839.1329	2514.3767	3	0.01	5.07	34.33	46.9 (M.expect:0.0,M.score:46.9)	EF1A1_HUMAN	Elongation factor 1-alpha 1 OS = Homo sapiens GN = EEF1A1 PE = 1 SV = 1

510	5924	844.4652	2530.3717	3	0.88	13.51	32.75	43.9 (M.expect:0.0,M.score:43.9)	EF1A1_HUMAN	Elongation factor 1-alpha 1 OS = Homo sapiens GN = EEF1A1 PE = 1 SV = 1
511	6415	1272.6335	2543.2504	2	0.80	8.13	33.37	33.8 (M.expect:0.0,M.score:33.8)	FLNA_HUMAN	Filamin-A OS = Homo sapiens GN = FLNA PE = 1 SV = 4
512	7716	1280.6286	2559.2413	2	0.56	9.14	26.58	48.8 (M.expect:0.0,M.score:48.8)	CH60_HUMAN	60 kDa heat shock protein, mitochondrial OS = Homo sapiens GN = HSPD1 PE = 1 SV = 2
513	8427	1300.6043	2599.1827	2	4.34	5.05	45.30	26.4 (M.expect:0.0,M.score:26.4)	UBA1_HUMAN	Ubiquitin-like modifier-activating enzyme 1 OS = Homo sapiens GN = UBA1 PE = 1 SV = 3
514	12341	1318.7186	2635.4109	2	4.49	7.34	51.58	35.9 (M.expect:0.0,M.score:35.9)	CO6A3_HUMAN	Collagen alpha-3(VI) chain OS = Homo sapiens GN = COL6A3 PE = 1 SV = 5
515	10005	897.4796	2689.4174	3	-0.12	10.05	47.60	42.1 (M.expect:0.0,M.score:42.1)	BGH3_HUMAN	Transforming growth factor-beta-induced protein ig-h3 OS = Homo sapiens GN = TGFBI PE = 1 SV = 1
516	9268	1353.0904	2704.1538	2	4.62	7.97	33.27	28.0 (M.expect:0.0,M.score:28.0)	K1C9_HUMAN	Keratin, type I cytoskeletal 9 OS = Homo sapiens GN = KRT9 PE = 1 SV = 3
517	8626	911.1444	2730.4129	3	-0.58	8.69	48.19	46.1 (M.expect:0.0,M.score:46.1)	CO6A3_HUMAN	Collagen alpha-3(VI) chain OS = Homo sapiens GN = COL6A3 PE = 1 SV = 5
518	283	919.1026	2754.2832	3	1.02	9.01	26.32	27.8 (M.expect:0.0,M.score:27.8)	CO3_HUMAN	Complement C3 OS = Homo sapiens GN = C3 PE = 1 SV = 2
519	17333	1412.6959	2823.3643	2	4.55	5.86	63.06	39.6 (M.expect:0.0,M.score:39.6)	TENA_HUMAN	Tenascin OS = Homo sapiens GN = TNC PE = 1 SV = 3

520	16	952.1419	2853.4005	3	1.14	5.06	26.48	126.3 (M.expect:0.0,M.score:126.3)	K1C18_HUMAN	Keratin, type I cytoskeletal 18 OS = Homo sapiens GN = KRT18 PE = 1 SV = 2
521	6086	965.1882	2892.5444	3	-0.53	17.64	45.95	27.8 (M.expect:0.0,M.score:27.8)	FLNA_HUMAN	Filamin-A OS = Homo sapiens GN = FLNA PE = 1 SV = 4
522	15386	1456.2766	2910.5266	2	4.10	22.12	54.47	56.5 (M.expect:0.0,M.score:56.5)	HSP7C_HUMAN	Heat shock cognate 71 kDa protein OS = Homo sapiens GN = HSPA8 PE = 1 SV = 1
523	13300	1006.4996	3016.4724	3	1.48	6.25	50.41	59.2 (M.expect:0.0,M.score:59.2)	MYH9_HUMAN	Myosin-9 OS = Homo sapiens GN = MYH9 PE = 1 SV = 4
524	7107	1038.5915	3112.7496	3	0.99	8.04	44.79	28.2 (M.expect:0.0,M.score:28.2)	H2A2B_HUMAN	Histone H2A type 2-B OS = Homo sapiens GN = HIST2H2AB PE = 1 SV = 3
525	9052	796.9646	3183.8231	4	1.94	12.12	41.53	39.8 (M.expect:0.0,M.score:39.8)	H2A1H_HUMAN	Histone H2A type 1-H OS = Homo sapiens GN = HIST1H2AH PE = 1 SV = 3
526	219	1242.5827	3724.7129	3	3.60	9.39	38.98	84.6 (M.expect:0.0,M.score:84.6)	K2C8_HUMAN	Keratin, type II cytoskeletal 8 OS = Homo sapiens GN = KRT8 PE = 1 SV = 7
527	15283	1481.7251	5922.8375	4	5.72	9.34	57.98	38.9 (M.expect:0.0,M.score:38.9)	FINC_HUMAN	Fibronectin OS = Homo sapiens GN = FN1 PE = 1 SV = 4
